# Prevalence of Hypertension and Albuminuria in Pediatric Type 2 Diabetes: A Systematic Review and Meta-analysis

**DOI:** 10.1001/jamanetworkopen.2021.6069

**Published:** 2021-04-30

**Authors:** Milena Cioana, Jiawen Deng, Maggie Hou, Ajantha Nadarajah, Yuan Qiu, Sondra Song Jie Chen, Angelica Rivas, Laura Banfield, Rahul Chanchlani, Allison Dart, Brandy Wicklow, Haifa Alfaraidi, Ahlam Alotaibi, Lehana Thabane, M. Constantine Samaan

**Affiliations:** 1 Department of Pediatrics, McMaster University, Hamilton, Ontario, Canada; 2 Division of Pediatric Endocrinology, McMaster Children’s Hospital, Hamilton, Ontario, Canada; 3 Health Sciences Library, McMaster University, Hamilton, Ontario, Canada; 4 Division of Pediatric Nephrology, McMaster Children’s Hospital, Hamilton, Ontario, Canada; 5 Department of Pediatrics and Child Health, University of Manitoba, Winnipeg, Manitoba, Canada; 6 Children’s Hospital Research Institute of Manitoba, University of Manitoba, Winnipeg, Manitoba, Canada; 7 College of Medicine, King Saud bin Abdulaziz University for Health Sciences, Division of Endocrinology, Department of Pediatrics, Ministry of the National Guard Health Affairs, Riyadh, Saudi Arabia; 8 Department of Pediatrics, Division of Pediatric Endocrinology, King Abdullah bin Abdulaziz University Hospital, Princess Noura University, Riyadh, Saudi Arabia; 9 Department of Health Research Methods, Evidence, and Impact, McMaster University, Hamilton, Ontario, Canada; 10 Department of Anesthesia, McMaster University, Hamilton, Ontario, Canada; 11 Centre for Evaluation of Medicines, St Joseph’s Health Care, Hamilton, Ontario, Canada; 12 Biostatistics Unit, St Joseph’s Healthcare, Hamilton, Ontario, Canada; 13 Michael G. De Groote School of Medicine, McMaster University, Hamilton, Ontario, Canada

## Abstract

**Importance:**

Hypertension and albuminuria are markers of diabetes-related nephropathy and important factors associated with kidney outcomes in pediatric type 2 diabetes. However, their prevalence in these patients is unknown.

**Objective:**

To measure the prevalence of hypertension and albuminuria in pediatric patients with type 2 diabetes and to evaluate the association of sex and race/ethnicity with these conditions.

**Data Sources:**

MEDLINE, Embase, CINAHL, Cochrane Library, Web of Science, the gray literature, and references of the screened articles were searched for human studies from date of database inception to February 20, 2020.

**Study Selection:**

Observational studies with at least 10 participants reporting the prevalence of hypertension and/or albuminuria in pediatric patients with type 2 diabetes were included. Three teams of 2 independent reviewers screened 7614 papers, of which 60 fulfilled the eligibility criteria.

**Data Extraction and Synthesis:**

Three teams of 2 independent reviewers performed data extraction, risk of bias analysis, and level of evidence analyses. The meta-analysis was conducted using a random-effects model and followed the Meta-analysis of Observational Studies in Epidemiology (MOOSE) guidelines.

**Main Outcomes and Measures:**

The primary outcomes included the pooled prevalence rates (percentages with 95% CI) for hypertension and albuminuria. The secondary outcomes assessed pooled prevalence rates by sex and racial/ethnic group.

**Results:**

Sixty studies were included in the systematic review. Diabetes duration varied from inclusion at diagnosis to 15.0 years after diagnosis, and the reported mean age at diagnosis ranged from 6.5 to 21.0 years. Hypertension prevalence among 3463 participants was 25.33% (95% CI, 19.57%-31.53%). Male participants had higher hypertension risk than female participants (odds ratio [OR], 1.42 [95% CI, 1.10-1.83]), with Pacific Islander and Indigenous youth having the highest prevalence of all racial/ethnic groups (Pacific Islander youth: 26.71% [95% CI, 14.54%-40.72%]; Indigenous youth: 26.48% [95% CI, 17.34%-36.74%]; White youth: 20.95% [95% CI, 12.65%-30.57%]; African American youth: 19.04% [95% CI, 12.01%-27.23%]; Hispanic/Latino youth: 15.11% [95% CI, 6.56%-26.30%]; Asian youth: 18.37% [95% CI, 9.49%-29.23%]). Albuminuria prevalence among 2250 participants was 22.17% (95% CI, 17.34%-27.38%). Pacific Islander youth, Indigenous youth, and Asian youth had higher prevalence rates than White youth (Pacific Islander youth: 31.84% [95% CI, 11.90%-55.47%]; Indigenous youth: 24.27% [95% CI, 14.39%-35.73%]; Asian youth: 23.00% [95% CI, 18.85%-27.41%]; White youth: 12.59% [95% CI, 7.75%-18.33%]), with no sex differences (OR for male vs female participants, 0.68 [95% CI, 0.46-1.01]). Heterogeneity was high among studies, with a low to moderate risk of bias.

**Conclusions and Relevance:**

In this study, markers of diabetes-related nephropathy were commonly detected in pediatric patients with type 2 diabetes, with a disproportionate burden noted among Pacific Islander and Indigenous youth. Personalized management strategies to target kidney outcomes are urgently needed in pediatric patients with type 2 diabetes to alleviate the burden of this condition on the kidneys.

## Key Points

**Question:**

What is the prevalence of hypertension and albuminuria in children and adolescents with type 2 diabetes?

**Findings:**

This systematic review and meta-analysis of 60 studies found that 25% of children and adolescents with type 2 diabetes had hypertension and 22% had albuminuria. Pacific Islander and Indigenous youth had a higher risk of these conditions than children from other racial groups.

**Meaning:**

In this study, the burden of hypertension and albuminuria in pediatric type 2 diabetes was substantial, especially among Pacific Islander and Indigenous youth.

## Teaser

This systematic review and meta-analysis measures the prevalence of hypertension and albuminuria in pediatric patients with type 2 diabetes and evaluates the association of sex and race/ethnicity with these conditions.

## Introduction

The global increase in obesity has driven the emergence of type 2 diabetes in children.^1,2^ Pediatric type 2 diabetes is an aggressive disease with greater risk of end-organ damage and comorbidities than pediatric type 1 diabetes or adult-onset type 2 diabetes.^1,2,3,4,5^ The kidneys are notable early targets of type 2 diabetes–associated organ damage, and diabetes-related nephropathy commonly manifests as hypertension and albuminuria.^6,7,8^ If untreated, hypertension is associated with cardiovascular anomalies, including increased carotid intima-media thickness and left ventricular hypertrophy.^9,10^ These subclinical adverse outcomes are known risk factors for future cardiovascular disease and mortality.^9,10^ Similarly, microalbuminuria is the first sign of diabetes-related nephropathy and can progress to chronic kidney disease and end-stage kidney disease if untreated.^11^

To ensure early detection and treatment of nephropathy in the pediatric type 2 diabetes population, current screening guidelines recommend measuring blood pressure (BP) and urine albumin-to-creatine ratio (ACR) at type 2 diabetes diagnosis and annually thereafter.^9,12,13^ With adequate glycemic and blood pressure control, the onset of end-stage kidney disease can be delayed, and the risk of microvascular and macrovascular complications can be reduced, making the management of hypertension and albuminuria crucial to improving outcomes in patients with pediatric type 2 diabetes.^11,14^ However, the full burden of diabetes-related nephropathy in pediatric patients with type 2 diabetes is not well established. There has also been some evidence suggesting that the rate of type 2 diabetes complications differs by sex and race/ethnicity.^15,16,17^ Determining how sex and race/ethnicity are associated with hypertension and albuminuria prevalence is an important step toward identifying at-risk groups and can inform future personalized screening and treatment strategies.

Thus, this systematic review aimed to determine the prevalence of hypertension and albuminuria in pediatric patients with type 2 diabetes and to explore the association of sex and race/ethnicity with prevalence.

## Methods

### Systematic Review Protocol and Registration

This systematic review has been registered with PROSPERO (CRD42018091127).^18^ The study is reported according to the Meta-analysis of Observational Studies in Epidemiology (MOOSE) reporting guideline.^19^

### Search Strategies

Search strategies were developed by a senior health sciences librarian and conducted in MEDLINE, Embase, CINAHL, Cochrane Central Register of Controlled Trials, and Cochrane Database of Systematic Reviews from database inception to February 20, 2020, without language restrictions (eTables 1-5 in the Supplement). The gray literature, including ClinicalTrials.gov and Web of Science: Conference Proceedings Citation Index–Science, was searched. We combined concepts of pediatrics and type 2 diabetes with terms for hypertension, albuminuria, prevalence, and epidemiologic study design. We also searched the references of included articles. If a conference abstract was deemed eligible, we sought a full-text publication and contacted the corresponding author if a published article could not be located or did not report the relevant data set for this analysis.

### Eligibility Criteria

We included studies with observational designs, including retrospective and prospective cohort studies as well as cross-sectional studies. The eligibility criteria included studies involving human participants with a sample size of at least 10 that reported on hypertension and/or albuminuria prevalence in patients with type 2 diabetes who were 18 years of age or younger. For studies with serial reporting of data, we included the report with the largest sample size. We excluded studies reporting participants with gestational diabetes.

To be as comprehensive as possible, we included studies reporting on all definitions of hypertension and albuminuria for our prevalence estimate. In the meta-analysis, we only pooled studies with similar definitions. Hypertension was defined as systolic and/or diastolic BP levels in the 95th percentile or greater for sex, age, and height.^20^ Most studies used BP reference values based on the National Heart, Lung, and Blood Institute data, whereas some used reference values based on the European guidelines.^20,21,22,23,24,25^ Urine ACR of 30 mg/g or greater defined albuminuria.^9,12,13,26^ Microalbuminuria was defined as an ACR of 30 or greater to 300 mg/g, and macroalbuminuria was defined as an ACR of greater than 300 mg/g. Persistent albuminuria, microalbuminuria, or macroalbuminuria were defined as 2 of 3 samples with levels greater than the corresponding ACR threshold over 6 months. If not specified, it was assumed that measurements were taken only once. Studies using other definitions of hypertension or albuminuria were removed in the sensitivity analysis.

### Study Selection, Data Abstraction, and Quality Appraisal

Three teams of 2 independent reviewers (M.C., M.H., A.N., Y.Q., S.J.J.C., A.R.) screened titles, abstracts, and full-text articles and completed data abstraction, risk of bias assessments, and level of evidence assessments. Reviewers resolved disagreements through discussion, and a third reviewer (M.C.S.) resolved persistent disagreements.

A data abstraction form was designed and piloted specifically for this study. We extracted data on study design, age at diabetes diagnosis, age at study enrollment, duration of diabetes, sample size, sex, and race. We also extracted hypertension and albuminuria definitions and prevalence with sex-specific and race-specific data, if reported.

For longitudinal studies, we extracted the prevalence values closest to the time of type 2 diabetes diagnosis because we wanted to define the prevalence closest to the time of hypertension or albuminuria diagnosis. For unreported data, we contacted the corresponding authors to retrieve the information specific to our study question. Several studies reported on cohorts that included participants older than 18 years. We contacted the study authors to retrieve pediatric-specific data. When we did not receive the data, we included studies if most participants were 18 years or younger and no participants were older than 25 years.

Risk of bias was evaluated for each study using a validated tool for prevalence studies developed by Hoy et al.^27^ The tool assesses methodological quality across 10 items addressing studies’ external and internal validity.^27^ Each criterion was given a score of 0 if unaddressed or unclear and 1 if it was met.^27^ External validity criteria included whether the target population was representative of the national population, the sampling frame was representative of the target population, random selection or a census was used, and whether there was limited evidence of nonresponse bias.^27^ Internal validity criteria included whether data were collected directly from participants, an acceptable case definition was used, reliable and valid tools were used to assess prevalence, data were collected using the same method for all participants, the length of the shortest prevalence period for the parameter of interest was appropriate, and, if appropriate, numerators and denominators were used to assess prevalence.^27^ The overall risk of bias was rated as low (score >8), moderate (score 6-8), or high (score ≤5).^27^ The overall level of evidence was assessed according to the Oxford Centre for Evidence-Based Medicine criteria (OCEBM).^28^

### Statistical Analysis

A random-effects model meta-analysis was performed when 2 or more studies of similar design, populations, methods, and outcomes were available.^29,30^ The primary outcomes of this review included the pooled prevalence of hypertension and albuminuria (reported as a percentage with 95% CIs) across all study designs. Because it was expected that some reports would have a small number of events, we transformed all prevalence estimates using the Freeman-Tukey double arcsine method^30^ and converted the results back to prevalence estimates for reporting.^31,32^ Inconsistency index (*I*^2^) and χ^2^ test *P* values were used to quantify heterogeneity among studies, and an *I*^2^ greater than 75% and *P* < .10 defined significant heterogeneity.^33^ Prespecified subgroup, sensitivity, metaregression, and publication bias assessments were performed if at least 10 studies were included in the meta-analysis for a given outcome.^33^ Subgroup analyses were performed by sex and race.

We also did a meta-analysis with studies comparing the prevalence between male and female participants and calculated odds ratios with 95% CIs. We used the National Institutes of Health definitions to categorize racial groups.^34^ We used the term Indigenous to report data from Indigenous populations in North America.

We also performed a random-effects metaregression to determine the association of obesity prevalence with hypertension and albuminuria. We performed sensitivity analyses by removing conference abstracts, studies with a sample size smaller than 50 patients, patients older than 18 years, or studies that used different or unspecified definitions of hypertension or albuminuria. A funnel plot was used to investigate publication bias with the Egger test and visual inspection to assess plot asymmetry.^35^ The prevalence meta-analyses were conducted using the metafor package in RStudio version 1.1.383, R version 3.4.3 (R Project for Statistical Computing).^36,37,38^ The sex-based forest plots for ORs were generated using Review Manager Version 5.3 (Cochrane Collaboration).^39^

## Results

### Search Results

The searches yielded 7614 unique records, and 60 eligible studies were included in the review (eFigure 1 in the Supplement). Most articles were removed, as they were irrelevant to the research question, reported on adult type 2 diabetes, or did not assess hypertension and/or albuminuria prevalence in children and adolescents with type 2 diabetes.

### Hypertension

#### Study Characteristics

Forty-six studies reported hypertension prevalence (Table 1).^4,15,17,40,41,42,43,44,45,46,47,48,49,50,51,52,53,54,55,56,57,58,59,60,61,62,63,64,65,66,67,68,69,70,71,72,73,74,75,76,77,78,79,80,81,82^ The reported age at diagnosis of type 2 diabetes ranged from 7.1 to 20.0 years,^46,47,49,72^ and the duration of diabetes ranged from inclusion at diagnosis^40,41,43,51,52,55,56,60,65,66,75,76,81^ to 7.8 years after diagnosis.^71^ While 26 studies (57%) had a cross-sectional design,^15,17,40,41,42,43,44,45,46,47,48,49,50,51,52,53,54,55,56,57,58,75,76,77,78,82^ 13 (28%) were retrospective cohort studies,^59,60,61,62,63,64,65,66,67,68,69,79,80^ and 7 (15%) were prospective cohort studies.^4,70,71,72,73,74,81^

**Table 1.** Characteristics of Included Studies Reporting on Prevalence of Hypertension in Pediatric Type 2 Diabetes

Source	Country	Study design	Age at diagnosis, mean (SD), y	Age at enrollment, mean (SD), y	Diabetes duration, mean (SD), y	Cases, No. (%)	Sample size, No.	Sex distribution No. (%)	Racial group distribution, No. (%)	Cases by sex and/or racial group, No. (%)	Hypertension definition	Reference values source	Prevalence of obesity, No. (%)
**Hypertension**													
Pinhas-Hamiel et al,^40^ 1996	United States	CS	13.8 (1.9)	13.8 (1.9)	0	9 (17)	54	M: 20 (37); F: 34 (63)	NHB: 37 (68), NHW: 17 (32)	NR	NR	NR	50 (92)
Scott et al,^41^ 1997	United States	CS	13.9 (0.4)^a^	13.9 (0.4)^a^	0	14 (32)	44	M: 17 (38); F: 27 (62)	AA: 32 (74); NHW: 11 (24); H: 1 (2)	NR	NR	NR	42 (85)^b^
Ettinger et al,^42^ 2005	United States	CS	NR	15.0 (1.9)	1.5 (1.0)	15 (58)	26	M: 12 (46); F: 14 (54)	H: 15 (58); NHB: 8 (31); other: 2 (7); multiracial: 1 (4)	NR	BP >95th percentile for sex and height	NHLBI Update on Second Task Force Report^23^	NR
Reinehr et al,^43^ 2005	Germany	CS	14.2 (13.0-15.0)^c^	14.2 (13.0-15.0)^c^	0	8 (50)	16	M: 10 (63); F: 6 (37)	White: 16 (100)	White: 8 (50)	BP ≥95th percentile for age, gender, and height	US age-sex-height specific values^21^	14 (88)
Eppens et al,^44^ 2006	Western Pacific	CS	12.0 (10.7-13.5)^b^^,^^c^	14.9 (13.2-16.4)^b^^,^^c^	2.3 (1.4-3.6)^b^^,^^c^	64 (24.2)	265	M: 120 (45.3); F: 145 (54.7)	NR	M: 34 (28.3); F: 30 (20.7)	Systolic and diastolic BP >95th percentile for age, sex, and height	NHLBI Update on Second Task Force Report^23^	106 (32.0)^b^
Unnikrishnan et al,^45^ 2008	India	CS	16.2 (2.9)	18.9 (4.9)	NR	1 (3)	36	M: 21 (58); F: 15 (42)	Indian: 36 (100)^d^	Indian: 1 (3)	NR	NR	NR
Bell et al,^46^ 2009	United States	CS	range, 10-19	Age 10-14 y: 41 (38.4%); age ≥15 y: 65 (61.3%)	NR	17 (16.0)	106	NR	NHW: 106 (100.0)	NHW: 17 (16.0)	BP ≥95th percentile for age, sex, and height	NHLBI Fourth Report^20^	83 (79.0)^b^
Dabelea et al,^47^ 2009	United States	CS	All participants <20	18.0 (2.8)	3.5 (2.2)	22 (36)	62	NR	Navajo: 62 (100)	Navajo: 22 (36)	BP ≥95th percentile for age, sex, and height	NHLBI Fourth Report^20^	42 (68)
Lawrence et al,^48^ 2009	United States	CS	Age 10-14 y: 11.6 (1.5); age ≥15 y: 14.6 (2.1)^b^	Age 10-14 y: 37 (30.8%); age ≥15 y: 83 (69.2%)	Age 10-14 y: 1.2 (0.9); age ≥15 y: 2.2 (2.0)^b^	25 (20.8)	120	NR	H: 120 (100.0)	H: 25 (20.8)	Systolic and/or diastolic BP ≥95th percentile for sex, age, and height	NHLBI Fourth Report^20^	93 (73.2)^b^^,^^e^
Liu et al,^49^ 2009	United States	CS	All participants <20	NR	Asian: 1.6 (1.4); API: 3.4 (3.1); PI: 1.7 (1.7)^b^	13 (27)	48	NR	Asian: 29 (60); API: 11 (23); PI: 8 (17)	Asian: 8 (28); API: 4 (36); PI: 1 (13)	Systolic or diastolic BP ≥95th percentile based on age, sex, and height	NHLBI Fourth Report^20^	38 (76)^b^
Mayer-Davis et al,^50^ 2009	United States	CS	Age 10-14 y: 11.7 (1); age ≥15 y: 15.1 (1.9)	Age 10-14 y: 81 (38.2%); age ≥15 y: 131 (61.8%)	Age 10-14 y: 1.2 (0.7); age ≥15 y: 2.6 (2.1)	49 (23.1)	212	NR	AA: 212 (100.0)	AA: 49 (23.1)	Systolic or diastolic BP ≥95th percentile for age, height, and sex	NHLBI Fourth Report^20^	NR
Urakami et al,^51^ 2009	Japan	CS	12.9 (1.5)	12.9 (1.5)	0	13 (11.6)	112	M: 45 (40.2); F: 67 (59.8)	Japanese: 112 (100.0)^d^	Japanese: 13 (11.6)	Systolic BP ≥130 mm Hg and/or diastolic BP ≥85 mm Hg	NR	93 (83.0)
Rodriguez et al,^17^ 2010	United States	CS	12.9 (2.1)	14.8 (2.0)	1.6 (1.5)	97 (23.7)	410	M: 152 (37.1); F: 258 (62.9)	AA: 130 (31.7); H: 99 (24.1); NHW: 84 (20.5); AI: 56 (13.7); API: 37 (9.0); other: 4 (1.0)	M: 38 (25.0); F: 59 (22.9); AA: 36 (27.7); H: 22 (22); NHW: 17 (20); AI: 12 (21); API: 10 (27); other: 0	Systolic or diastolic BP >95th percentile for age, sex, and height	NHLBI Fourth Report^20^	332 (81.0)
Copeland et al,^15^ 2011	United States	CS	range, 10-17	14.0 (2.0)	0.7 (0.5)	96 (13.6)	704	M: 247 (35.1); F: 457 (64.9)	H: 289 (41.1); NHB: 222 (31.5); NHW: 138 (19.6); AI: 43 (6.1); Asian: 12 (1.7)	M: 44 (17.8); F: 52 (11.4); H: 31 (10.7); NHB: 34 (15.3); NHW: 23 (16.7); AI: 7 (16)	BP ≥95th percentile for age, sex and height	NR	NR
Amed et al,^52^ 2012	Canada	CS	Canadian Aboriginal: 12.9 (12.4-13.4)^f^; White: 14.4 (13.8-15.1)^f^; other (African/Caribbean, Asian, H, Middle Eastern): 14.3 (13.7-14.9)^f^	Canadian Aboriginal: 12.9 (12.4-13.4)^f^, White: 14.4 (13.8-15.1)^f^, other (African/Caribbean, Asian, H, Middle Eastern): 14.3 (13.7-14.9)^f^	0	61 (27.8)	221	M: 91 (41.2); F: 130 (58.8)	Canadian Aboriginal: 100 (45.2); White: 57 (25.8); other (African/Caribbean, Asian, H, Middle Eastern): 64 (29.0)	Canadian Aboriginal: 27 (27.3); White: 16 (28); other (African/Caribbean, Asian, H, Middle Eastern): 18 (28)	NR	NR	211 (95.3)
Amutha et al,^53^ 2012	India	CS	NR	16.1 (2.5)	22.2 (9.7)	47 (23.7)	198	M: 81 (40.9); F: 117 (59.1)	South Indian: 198 (100.0)^d^	M: 22 (27); F: 25 (21.4); South Indian: 47 (23.7)	BP ≥130/85 mm Hg	NR	NR
Drutel and Paulo,^54^ 2014^g^	United States	CS	NR	range, 3-18	NR	73 (27.8)	263	NR	NR	NR	NR	NR	NR
Klingensmith et al,^55^ 2016	United States	CS	13.1 (2.3)^b^	13.1 (2.3)^b^	0	44 (29.3)	150	NR	NR	NR	BP ≥95th percentile for age and height	NR	315 (62.6)^b^
Zabeen et al,^56^ 2016	Bangladesh	CS	Age 9-10 y: 11 (14.3%); age 11-14 y: 46 (59.7%); age 15-17 y: 20 (26.0%)	Age 9-10 y: 11 (14.3%); age 11-14 y: 46 (59.7%); age 15-17 y: 20 (26.0%)	0	25 (32)	77	M: 26 (34); F: 51 (66)	Bangladeshi: 77 (100)^d^	Bangladeshi: 25 (32)	BP ≥95th percentile for age and sex	NHLBI Second Task Force Report^22^	45 (58)
Aulich et al,^57^ 2019	Australia	CS	NR	15.1 (1.9)	1.8 (0.3-3.3)^c^	6 (19)	31	NR	NR	NR	BP ≥95th percentile for age and sex for patients <18 y, ≥140/90 mm Hg for patients ≥18 y, or relevant medical therapy	NHLBI Second Task Force Report^22^	24 (75)^b^
Khalil et al,^58^ 2019	Egypt	CS	18.0 (2.0)	19.8 (1.1)	2.5 (2.0)	1 (8)	13	M: 6 (46); F: 7 (54)	Egyptian: 13 (100)^d^	M: 1 (17); F: 0; Egyptian: 0	Systolic BP ≥140 mm Hg or diastolic BP ≥80 mm Hg or taking antihypertensive medication	NR	NR
Scott et al,^59^ 2004	New Zealand	RC	NR	mean, 19.6; range, 14-23	1.7	5 (39)	13	M: 7 (54); F: 6 (46)	Maori: 7 (54); European: 4 (30); PI: 1 (8); Asian Indian: 1 (8)	NR	Systolic BP >130 mm Hg or diastolic BP >80 mm Hg	NR	13 (100)
Zdravkovic et al,^60^ 2004	Canada	RC	13.5 (2.2) [range, 8.8-17.5]	13.5 (2.2) [range, 8.8-17.5]	0	4 (10)	41	M: 15 (37); F: 26 (63)	South/East Asian: 19 (46); African Canadian: 11 (27); NHW: 6 (15); H: 4 (10); FN: 1 (2)	NR	BP ≥95th percentile for age and sex	NHLBI Update on Second Task Force Report^23^	33 (80)
Pérez-Perdomo et al,^61^ 2005	Puerto Rico	RC	NR	Age ≤17 y: 35 (80%); age 18-19 y: 9 (20%)	NR	5 (11)	44	NR	NR	NR	NR	NR	69 (80)^b^
Scott et al,^62^ 2006	New Zealand	RC	NR	20.0 (0.4)	3.0 (0.3)	21 (20.0)	105	NR	Maori/PI/other: 66 (62.9); European: 39 (37.1)	NR	BP >130/85 mm Hg	NR	105 (100)
Balasanthiran et al,^63^ 2012	United Kingdom	RC	15.2 (3.3)	21.2 (3.2)	5.4 (3.1)	9 (21)	44	M: 17 (39); F: 27 (61)	Bangladeshi: 11 (25); Pakistani: 9 (20); Indian: 7 (16); White British: 6 (14); Black African: 4 (9); Black Caribbean: 4 (9); unclear: 3 (7)	NR	BP persistently >130/85 mm Hg	NR	23 (59)^b^^,^^e^
Osman et al,^64^ 2013	Sudan	RC	Age <11 y: 3 (8%); age 11-18 y: 35 (92%)	NR	NR	22 (58)	38	M: 17 (45); F: 21 (55)	Arab: 32 (84); multiracial: 4 (11); non-Arab: 2 (5)	NR	BP ≥95th percentile for age and sex on >1 occasion	NR	29 (76)
Dart et al,^65^ 2014	Canada	RC	13.5 (2.2)	13.5 (2.2)	0	34 (9.9)	342	M: 129 (37.8); F: 213 (62.2)	NR	NR	Elevated BP for age, sex, and height	NHLBI Fourth Report^20^	NR
Haynes et al,^66^ 2014^g^	Australia	RC	13.3 (2.0)^b^	13.3 (2.0)^b^	0	15 (20)	75	NR	NR	NR	NR	NR	82 (60.7)^b^
Yafi,^67^ 2019^g^	United States	RC	range, 8-15	NR	NR	1 (5)	25	M: 11 (44); F: 14 (56)	H: 15 (60); other: 10 (40)	NR	NR	NR	NR
Yeow et al,^68^ 2019	Malaysia	RC	14.3 (3.5)	20.7 (3.7)	6.5 (2.8)	3 (13)	24	M: 10 (42); F: 14 (58)	Malay: 12 (50); Chinese: 11 (46); Asian Indian: 1 (4)	NR	BP ≥95th percentile for age and sex for patients <17 y; BP ≥140/90 mm Hg for patients ≥17 y	NHLBI Fourth Report^20^; NHLBI Seventh Report^24^	10 (42)
Curran et al,^69^ 2020	Australia	RC	All participants <10	All participants, <16	NR	3 (27)	11	NR	PI: 11 (100)	PI: 3 (27)	NR	NR	11 (100)
Eppens et al,^4^ 2006	Australia	PC	13.2 (11.6-15.0)^b^^,^^c^	15.3 (13.6-16.4)^b^^,^^c^	1.3 (0.6-3.1)^b^^,^^c^	21 (36)	58	NR	NR	NR	Systolic or diastolic BP >95th percentile for age and sex	NHLBI Update on Second Task Force Report^23^	36 (56)^b^
Shield et al,^70^ 2009	United Kingdom and Republic of Ireland	PC	mean, 13.6; range, 9.9-16.8^b^	mean, 14.5; range, 10.8-17.8^b^	mean, 1^b^	19 (32)	59	M: 24 (41); F: 35 (59)	NR	M: 7 (29); F: 12 (34)	Systolic or diastolic BP ≥98th percentile for age and sex	UK Reference Values^25^	61 (80)^b^
Ruhayel et al,^71^ 2010	Australia	PC	13.4 (range, 9.2-17.4)^a^^,^^c^	M: 16.0 (13.6-18.2); F: 15.6 (11.7-19.8)^a^^,^^c^	M: 2.2 (0.0-7.8); F: 2.3 (0.1-7.4)^a^^,^^c^	9 (30)	30	NR	NR	NR	BP >98th percentile for age and sex	UK Reference Values^25^	23 (70)^b^
Jefferies et al,^72^ 2012	New Zealand	PC	12.9 (1.8) [range, 7.1-15.5]	NR	NR	27 (52)	52	M: 17 (33); F: 35 (67)	PI/Maori: 47 (90); other: 5 (10)	NR	BP ≥95th percentile for sex and age	NR	NR
Schmidt et al,^73^ 2012	Germany and Austria	PC	13.5 (3.4)	15.3 (3.0)	NR	202 (29.5)	684	M: 261 (38.2); F: 423 (61.8)	German/Austrian: 482 (70.5); other: 202 (29.5)	NR	BP >95th percentile for age, sex, and height	NHLBI Fourth Report^20^	NR
Dart et al,^74^ 2019	Canada	PC	All participants <18	15 (13.3-16.8)^c^	2.3 (0.9-4.1)^c^	155 (82.9)	187	M: 62 (33.2); F: 125 (66.8)	Indigenous: 179 (95.7); other: 8 (4.3)	NR	BP ≥95th percentile for age, sex and height	NHLBI Fourth Report^20^	NR
**Systolic hypertension**													
Upchurch et al,^75^ 2003	United States	CS	13.6 (2.3)^b^	13.6 (2.3)^b^	0	41 (49)	83	NR	NR	NR	Systolic BP ≥95th percentile for age, sex, and height	NR	91 (93)^b^
Wei et al,^76^ 2003	Taiwan	CS	M: 13.7 (2.5); F: 13.0 (2.5)^a^^,^^b^	M: 13.7 (2.5); F: 13.0 (2.5)^a^^,^^b^	0	49 (39.2)	125	M: 47 (37.6); F: 78 (62.4)	NR	M: 23 (49); F: 26 (33)	Systolic BP ≥85th percentile of sex and age based on population in the study	NR	63 (48.1)^b^
Cruz et al,^77^ 2004	Mexico	CS	All participants <18	13.8 (1.8)	mean, 0.9; range, 0.08-3	2 (5)	44	M: 20 (46); F: 24 (54)	Mexican: 44 (100)^d^	Mexican: 2 (5)	NR	NR	NR
Hotu et al,^78^ 2004	New Zealand	CS	mean, 15; range, 11-19	NR	NR	5 (28)	18	M: 9 (50); F: 9 (50)	Maori/PI: 18 (100)	M: 3 (33); F: 2 (22); Maori/PI: 5 (28)	Systolic BP >95th percentile for age, sex and height	NHLBI Update on Second Task Force Report^23^	NR
Rodriguez et al,^17^ 2010	United States	CS	12.9 (2.1)	14.8 (2.0)	1.6 (1.5)	106 (25.9)	410	M: 152 (37.1); F: 258 (62.9)	AA: 130 (31.7); H: 99 (24.1); NHW: 84 (20.5); AI: 56 (13.7); API: 37 (9.0); other: 4 (1.0)	NR	Systolic BP >95th percentile for age, sex and height	NHLBI Fourth Report^20^	332 (81.0)
Sellers et al,^79^ 2007	Canada	RC	mean, 13.1; range, 9-17	mean, 15.3; range, 9-18	NR	13 (13)	99	M: 42 (42); F: 57 (58)	FN/Metis: 94 (95); other: 5 (5)	M: 5 (12); F: 8 (14)	Systolic BP >95th percentile for age and gender	NHLBI Fourth Report^20^	38 (38)
Pelham et al,^80^ 2018	United States	RC	NR	15.2 (2.7)	2.7 (1.7)	34 (37)	93	M: 27 (29); F: 66 (71)	NR	NR	Systolic BP ≥95th percentile for age, gender, and height	NHLBI Fourth Report^20^	NR
Shield et al,^70^ 2009	United Kingdom and Republic of Ireland	PC	mean, 13.6; range, 9.9-16.8^b^	mean, 14.5; range, 10.8-17.8^b^	mean, 1^b^	4 (7)	59	M: 24 (41); F: 35 (59)	NR	M: 1 (4); F: 3 (9)	Systolic BP ≥98th percentile for age and sex	UK Reference Values^25^	61 (80)^b^
Candler et al,^81^ 2018	United Kingdom and Republic of Ireland	PC	14.3 (7.9-16.9)^h^	14.3 (7.9-16.9)^h^	0	22 (20.8)	106	M: 35 (33.0); F: 71 (67.0)	NHW: 47 (44.3); Asian/Asian British: 36 (34.0); BACBB: 14 (13.2); other: 5 (4.7); uncertain: 4 (3.8)	NR	Systolic BP ≥95th percentile for sex, age and height	NHLBI Fourth Report^20^	86 (81.1)
**Diastolic hypertension**													
Upchurch et al,^75^ 2003	United States	CS	13.6 (2.3)^b^	13.6 (2.3)^b^	0	9 (11)	83	NR	NR	NR	Diastolic BP ≥95th percentile for age, sex, and height	NR	91 (93)^b^
Wei et al,^76^ 2003	Taiwan	CS	M: 13.7 (2.5); F: 13.0 (2.5)^a^^,^^b^	M: 13.7 (2.5); F: 13.0 (2.5)^a^^,^^b^	0	53 (42.4)	125	M: 47 (37.6); F: 78 (62.4)	NR	M: 24 (51); F: 29 (37)	Diastolic BP ≥85th percentile of sex and age based on population in the study	NR	63 (48.1)^b^
Cruz et al,^77^ 2004	Mexico	CS	NR	13.8 (1.7)	mean, 0.9; range, 0.08-3	5 (11)	44	M: 20 (46); F: 24 (54)	Mexican: 44 (100)^d^	Mexican: 5 (11)	NR	NR	NR
Rodriguez et al,^17^ 2010	United States	CS	12.9 (2.1)	14.8 (2.0)	1.6 (1.5)	75 (18.3)	410	M: 152 (37.1); F: 258 (62.9)	AA: 130 (31.7); H: 99 (24.1); NHW: 84 (20.5); AI: 56 (13.7); API: 37 (9.0); other: 4 (1.0)	NR	Diastolic BP >95th percentile for age, sex and height	NHLBI Fourth Report^20^	332 (81.0)
Shalitin et al,^82^ 2014	Israel	CS	NR	15.9 (3.6)	3.3 (2.1)	4 (36)	11	M: 5 (45); F: 6 (55)	Israeli: 11 (100)^d^	Israeli: 4 (36)	NR	NR	11 (100)
Sellers et al,^79^ 2007	Canada	RC	mean, 13.1; range, 9-17	mean, 15.3; range, 9-18	NR	6 (6)	99	M: 42 (42); F: 57 (58)	FN/Metis: 94 (95); Other: 5 (5)	M: 3 (7); F: 3 (5)	Diastolic BP >95th percentile for age and gender	NHLBI Fourth Report^20^	38 (38)
Pelham et al,^80^ 2018	United States	RC	NR	15.2 (2.7)	2.7 (1.7)	7 (8)	93	M: 27 (29); F: 66 (71)	NR	NR	Diastolic BP ≥95th percentile for age, gender, and height	NHLBI Fourth Report^20^	NR
Shield et al,^70^ 2009	United Kingdom and Republic of Ireland	PC	mean, 13.6; range, 9.9-16.8^b^	mean, 14.5; range, 10.8-17.8^b^	mean, 1^b^	11 (19)	59	M: 24 (41); F: 35 (59)	NR	M: 6 (25); F: 5 (14)	Diastolic BP ≥98th percentile for age and sex	UK Reference Values^25^	61 (80)^b^

Abbreviations: AA, African American; AI, American Indian; API, Asian–Pacific Islander; BACBB, Black/African/Caribbean/Black British; BP, blood pressure; CS, cross-sectional; F, female; FN, First Nations; H, Hispanic; M, male; NHB, Non-Hispanic Black; NHLBI, National Heart, Lung, and Blood Institute; NHW, Non-Hispanic White; NR, not reported; PC, prospective cohort; PI, Pacific Islander; RC, retrospective cohort.

^a^ Mean (SE).

^b^ Based on total study cohort instead of only patients examined for the specific comorbidity.

^c^ Median (interquartile range).

^d^ Racial group distribution assumed to match country of origin.

^e^ Value estimated based on graph.

^f^ Mean (CI).

^g^ Abstract only.

^h^ Median (range).

#### Pooled Prevalence of Hypertension

**Figure 1.**
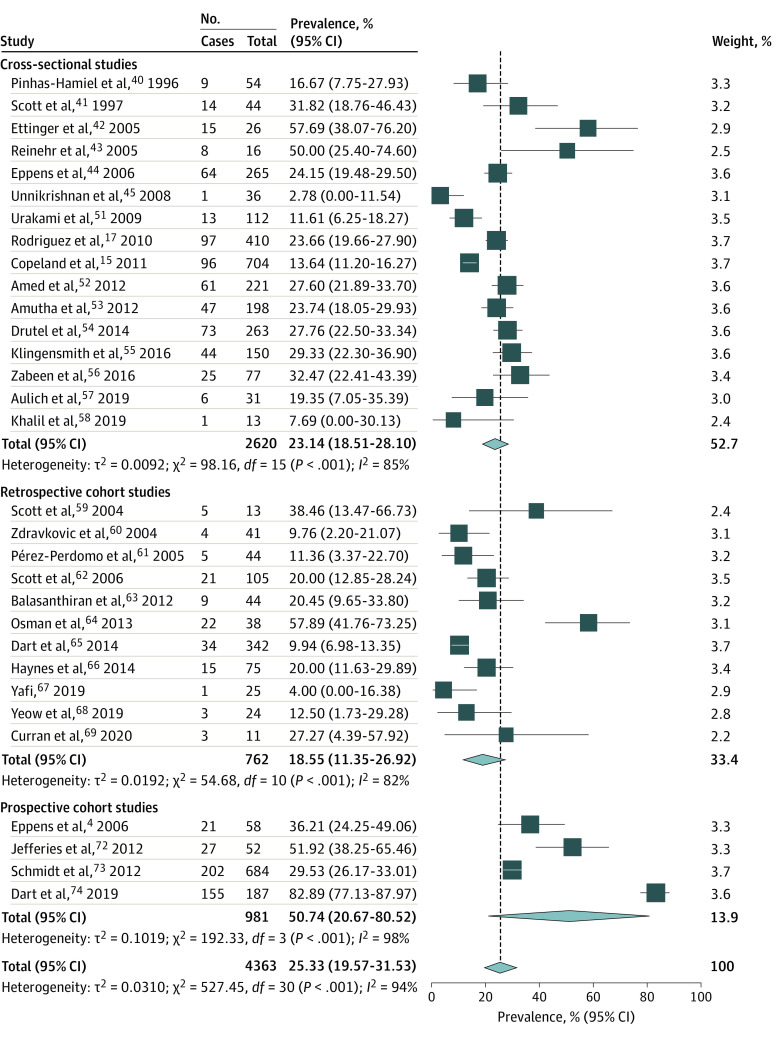
Forest Plot Showing Pooled Prevalence of Hypertension in Pediatric Type 2 Diabetes

Thirty-one studies including 4363 patients with type 2 diabetes reported on the prevalence of hypertension defined as BP in the 95th percentile or greater for age, sex, and height or systolic BP 130 to 140 mm Hg or greater and diastolic BP of 80 to 90 mm Hg or greater.^4,15,17,40,41,42,43,44,45,51,52,53,54,55,56,57,58,59,60,61,62,63,64,65,66,67,68,69,72,73,74^ The pooled prevalence of hypertension was 25.33% (95% CI, 19.57%-31.53%) (Figure 1). High heterogeneity was noted across studies (*I*^2^ = 94%; *P* < .001).

Another 15 studies that reported on the prevalence of hypertension were not included in the meta-analysis. Two studies had a higher cutoff for hypertension (BP ≥98th percentile) and reported a prevalence of 30% among 30 participants and 32% among 59 participants, respectively.^70,71^ Five studies presented racial subgroup data^46,47,48,49,50^ of another included study.^17^ Eight studies were only included in the analysis of isolated systolic or diastolic hypertension.^75,76,77,78,79,80,81,82^

When pooling only the studies with hypertension definition of BP in 95th percentile or greater for age, sex, and height (2763 participants), the prevalence was significantly higher at 34.00% (95% CI, 24.00%-45.00%; *I*^2^ = 97%; *P* < .001) (eTable 6 in the Supplement).^4,15,17,42,43,44,55,56,57,60,64,68,72,73,74^ The metaregression analysis revealed no significant association between hypertension prevalence and obesity prevalence.

#### Pooled Prevalence of Systolic and Diastolic Hypertension in Type 2 Diabetes

Isolated systolic hypertension prevalence across 6 studies with 747 participants was 24.79% (95% CI, 14.04%-37.31%; *I*^2^ = 90%; *P* < .001) (eFigure 2 in the Supplement).^17,75,77,78,79,80^ Two additional studies using different definitions reported a prevalence of 39.2% among 125 participants (hypertension definition, BP ≥85th percentile)^76^ and 7% among 59 participants (hypertension definition, BP ≥98th percentile).^70^ Another study determined a prevalence of 20.8% among 106 participants; because it was the only prospective cohort study, it was not included in the meta-analysis.^81^

Isolated diastolic hypertension prevalence across 6 studies with 740 participants was 11.65% (95% CI, 6.41%-18.04%; *I*^2^ = 75%; *P* = .001) (eFigure 3 in the Supplement).^17,75,77,79,80,82^ Two additional studies using different definitions reported a prevalence of 42.4% among 125 participants (diastolic hypertension, BP ≥85th percentile)^76^ and 19% among 59 participants (diastolic hypertension, BP ≥98th percentile).^70^

#### Sex and Race Associations With Hypertension

Four studies reported hypertension prevalence in 600 male participants of 23.81% (95% CI, 18.56%-29.47%; *I*^2^ = 58%; *P* = .07) and 977 female participants of 18.56% (95% CI, 12.25%-25.82%; *I*^2^ = 85%; *P* < .001) with an OR of 1.42 (95% CI, 1.10-1.83; *I*^2^ = 0%; *P *for heterogeneity = .65) (eFigure 4 and eFigure 5 in the Supplement).^15,17,44,53^ In contrast, 1 study with hypertension definition of BP in the 98th percentile or greater reported a prevalence of 29% in 24 male participants and 34% in 35 female participants.^70^

When assessing the prevalence of hypertension in different racial groups, Indigenous and Pacific Islander youth had the highest rates of hypertension when compared with other groups (Pacific Islander youth^17,69^: 48 participants; prevalence, 26.71% [95% CI, 14.54%-40.72%]; *I*^2^ = 0%; *P* = .92; Indigenous youth^15,47,52^: 205 participants; prevalence, 26.48% [95% CI, 17.34%-36.74%]; *I*^2^ = 58%, *P* = .09; White youth^15,43,46,52,58^: 330 participants; prevalence, 20.95% [95% CI, 12.65%-30.57%]; *I*^2^ = 66%; *P* = .02; African American youth^15,50^: 434 participants; prevalence, 19.04% [95% CI, 12.01%-27.23%]; *I*^2^ = 76%; *P* = .04; Hispanic/Latino youth^15,48^: 409 participants; prevalence, 15.11% [95% CI, 6.56%-26.30%]; *I*^2^ = 85%; *P* < .001; Asian youth^45,49,51,53,56^: 452 participants; prevalence, 18.37% [95% CI, 9.49%-29.23%]; *I*^2^ = 84%, *P* < .001) (eFigure 6 in the Supplement).

### Albuminuria

#### Study Characteristics

Thirty-nine studies reported on albuminuria prevalence (Table 2).^4,8,15,16,42,44,45,46,50,52,53,57,58,59,62,64,65,66,67,68,69,71,72,73,74,78,81,83,84,85,86,87,88,89,90,91,92,93,94^ The age at type 2 diabetes diagnosis ranged from 6.5 to 21.0 years,^90,93^ and type 2 diabetes duration ranged from diagnosis^52,65,66,71,81,83,85,89,94^ to more than 15.0 years after diagnosis.^50,53^ Nineteen studies (49%) were cross-sectional studies,^15,16,42,44,45,46,50,52,53,57,58,78,83,84,86,87,88,89,90^ 14 (36%) were retrospective cohort studies,^8,59,62,64,65,66,67,68,69,85,91,92,93,94^ and 6 (15%) were prospective cohort studies.^4,71,72,73,74,81^

**Table 2.** Characteristics of Included Studies Reporting on the Prevalence of Albuminuria in Pediatric Type 2 Diabetes

Source	Country	Study design	Age at diagnosis, mean (SD), y	Age at study enrollment or measurement, mean (SD), y	Duration of diabetes, mean (SD), y	Cases, No. (%)	Sample size	Sex distribution, No. (%)	Racial group distribution, No. (%)	Cases by sex or racial group, No. (%)	Albuminuria definition	Prevalence of obesity, No. (%)
**Albuminuria**												
Hotu et al,^78^ 2004	New Zealand	CS	mean, 15; range, 11-19	NR	NR	7 (58)	12	M: 6 (50); F: 6 (50)	Maori/PI: 12 (100)	M: 4 (67); F: 3 (50); Maori/PI: 7 (58)	ACR ≥30mg/g	NR
Ettinger et al,^42^ 2005	United States	CS	NR	15.0 (1.9)	1.5 (1.0)	10 (40)	25	M: 12 (46); F: 14 (54)^a^	H: 15 (58); NHB: 8 (31); other: 2 (7); multiracial: 1 (4)^a^	NR	AER ≥30mg albumin/24 h	NR
Maahs et al,^16^ 2007	United States	CS	All participants <20	Age <12 y: 19 (5.1%); age ≥12 y: 355 (94.9%)	1.9 (0.4-3.2)^b^	83 (22.2)	374	M: 140 (37.4); F: 234 (62.6)	AA: 110 (29.4); AI: 92 (24.6); NHW: 71 (19.0); H: 64 (17.1); API: 25 (6.8); multiracial or other: 11 (2.9); unknown: 1 (0.2)	M: 29 (20.7); F: 54 (23.1); AA: 18 (16.4); AI: 33 (36); NHW: 9 (13); H: 15 (23); API: 6 (24); multiracial or other: 2 (18); unknown: 0 (0)	ACR ≥30mg/g	266 (72.3)^a^
Unnikrishnan et al,^45^ 2008	India	CS	16.2 (2.9)	18.9 (4.9)	NR	0	36	M: 21 (58); F: 15 (42)	Indian: 36 (100)^c^	Indian: 0	AER >500mg albumin/24 h	NR
Bell et al,^46^ 2009	United States	CS	All participants <20	Age 10-14 y: 42 (41.6%); age ≥15 y: 59 (58.4%)	NR	14 (13.9)	101	NR	NHW: 101 (100)	NHW: 14 (13.9)	ACR ≥30mg/g	83 (79.0)^a^
Mayer-Davis et al,^50^ 2009	United States	CS	10-14: 11.7 (1); ≥15: 15.1 (1.9)	10-14: 81 (38.2%); ≥15: 131 (61.8%)	10-14: 1.2 (0.7); ≥15: 2.6 (2.1)	30 (14.1)	212	NR	AA: 212 (100)	AA: 30 (14.1)	ACR ≥30mg/g	NR
Kim et al,^83^ 2010	United States	CS	NR	14.5 (3.0)	mean, 1.3; range, 0.0-2.1	22 (21.4)	103	M: 40 (38.8); F: 63 (61.2)	Pima Indian: 103 (100)	Pima Indian: 22 (21.4)	ACR ≥30mg/g	NR
Amed et al,^52^ 2012	Canada	CS	Canadian Aboriginal: 12.9 (12.4-13.4)^d^; White: 14.4 (13.8-15.1)^d^; other (African/Caribbean, Asian, H, Middle Eastern): 14.3 (13.7-14.9)^d^	Canadian Aboriginal: 12.9 (12.4-13.4)^d^; White: 14.4 (13.8-15.1)^d^; other (African/Caribbean, Asian, H, Middle Eastern): 14.3 (13.7-14.9)^d^	0	32 (14.4)	221	M: 91 (41.2); F: 130 (58.8)	Canadian Aboriginal: 100 (45.2); White: 57 (25.8); other (African/Caribbean, Asian, H, Middle Eastern): 64 (29.0)	Canadian Aboriginal: 16 (16.7); White: 6 (10); other (African/Caribbean, Asian, H, Middle Eastern): 9 (14)	NR	211 (95.3)
Amutha et al,^53^ 2012	India	CS	16.1 (2.5)	22.2 (9.7)	Age ≤5 y: 219 (59.5%); age >5 to ≤10 y: 67 (18.2%); age >10 to ≤15: 21 (5.7%); age >15 y: 61 (16.6%)	85 (23.1)	368	M: 168 (45.7); F: 200 (54.3)	South Indian: 368 (100.0)^c^	South Indian: 85 (23.1)	ACR ≥30mg/g	NR
Holman et al,^84^ 2015	United Kingdom	CS	All participants >12	All participants, >12	NR	NR (23)	NR	NR	NR	NR	NR	NR
Sellers et al,^85^ 2009	Canada	RC	NR	NR	0	26 (29)	90	M: 40 (45); F: 50 (55)	FN/Metis: 88 (98); other: 2 (2)	NR	ACR >3 mg/mmol	NR
Yafi,^67^ 2019^e^	United States	RC	range, 8-15	NR	NR	3 (12)	25	M: 11 (44); F: 14 (56)	H: 15 (60); other: 10 (40)	NR	NR	NR
Yeow et al,^68^ 2019	Malaysia	RC	14.3 (3.5)	20.7 (3.7)	6.5 (2.8)	7 (29)	24	M: 10 (42); F: 14 (58)	Malay: 12 (50); Chinese: 11 (46); Asian Indian: 1 (4)	Asian: 7 (29)	M: ACR >2.5mg/ mmol, F: ACR >3.5mg/ mmol	10 (42)
Curran et al,^69^ 2020	Australia	RC	All participants <10	All participants, <16	NR	2 (18)	11	NR	PI: 11 (100)	PI: 2 (18)	NR	11 (100)
Ruhayel et al,^71^ 2010	Australia	PC	13.4 (9.2-17.4)^a^^,^^d^	M: 16.0 (13.6-18.2; F: 15.6 (11.7-19.8)^a^^,^^d^	M: 2.2 (0.0-7.8; F: 2.3 (0.1-7.4)^a^^,^^d^	9 (45)	20	NR	NR	NR	ACR >3.5mg/ mmol	23 (70)^a^
Schmidt et al,^73^ 2012	Germany and Austria	PC	13.5 (3.4)	15.3 (3.0)	NR	170 (24.9)	684	M: 261 (38.2); F: 423 (61.8)	German/Austrian: 482 (70.5); other: 202 (29.5)	NR	NR	NR
Candler et al,^81^ 2018	United Kingdom and Republic of Ireland	PC	14.3 (7.9-16.9)^d^	14.3 (7.9-16.9)^d^	0	3 (2.8)	106	M: 35 (33.0); F: 71 (67.0)	NHW: 47 (44.3); Asian/Asian-British: 36 (34.0); BACBB: 14 (13.2); other: 5 (4.7); uncertain: 4 (3.8)	NR	NR	86 (81.1)
Dart et al,^74^ 2019	Canada	PC	All participants <18	15 (13.3-16.8)^b^	2.3 (0.9-4.1)^b^	47 (25.1)	187	M: 62 (33.2); F: 125 (66.8)	Indigenous: 179 (95.7); other: 8 (4.3)	NR	ACR >2mg/mmol	NR
**Persistent albuminuria**												
Yoo et al,^86^ 2004	Korea	CS	12.8 (1.5)	18.4 (4.3)	5.5 (3.9)	5 (23)	22	M: 8 (36); M: 14 (64)	Korean: 22 (100)	Korean: 5 (23)	AER >20 μg/min on samples 3 mo apart	NR
Farah et al,^87^ 2006	United States	CS	NR	range, 10-21	mean, 1.8; range, <2-5	9 (27)	33	NR	NR	NR	ACR >30 mg/g on 2 samples within 3-6 mo	29 (73)^a^
Copeland et al,^15^ 2011	United States	CS	range, 10-17	14.0 (2.0)	0.7 (0.5)	92 (13.0)	704	M: 247 (35.1); F: 457 (64.9)	H: 289 (41.1); NHB: 222 (31.5); NHW: 138 (19.6); AI: 43 (6.1); Asian: 12 (1.7)	M: 26 (10.6); F: 65 (14.3); H: 41 (14.1); NHB: 25 (11.2); NHW: 20 (14.6); AI: 3 (8)	ACR ≥30mg/g on 2 of 3 samples during 3-mo period	NR
Sellers et al,^88^ 2016	Canada	CS	All participants <18	NR	NR	50 (5.1)	976	NR	NR	NR	M: ACR >2mg/ mmol, F: ACR >2.8mg/ mmol on 2 occasions during 6-mo period	NR
Aulich et al,^57^ 2019	Australia	CS	NR	15.1 (1.9)	1.8 (0.3-3.3)^b^	6 (30)	20	NR	NR	NR	AER ≥20 μg/min in ≥2 of 3 samples or mean ACR, M: ≥3.5 mg/mmol; F: ≥4 mg/mmol from 3 first morning collections	24 (75)^a^
Khalil et al,^58^ 2019	Egypt	CS	18.0 (2.0)	19.8 (1.1)	2.5 (2.0)	0 (0)	13	M: 6 (46); F: 7 (54)	Egyptian: 0^c^	Egyptian: 0	ACR ≥30mg/g on 2 samples within 3-6 mo	NR
Scott et al,^59^ 2004	New Zealand	RC	NR	mean, 19.6; range, 14-23	1.7	2 (15)	13	M: 7 (54); F: 6 (46)	Maori: 7 (54); European: 4 (30); PI: 1 (8); Asian Indian: 1 (8)	NR	M: ACR >2.5mg/mmol; F: ACR >3.5mg/mmol on ≥2 occasions	13 (100)
Scott et al,^62^ 2006	New Zealand	RC	NR	20.0 (0.4)	3.0 (0.3)	76 (72.4)	105	NR	Maori/PI/other: 66 (62.9); European: 39 (37.1)	NR	M: ACR >2.5mg/ mmol; F: ACR >3.5mg/ mmol on ≥2 occasions	105 (100)
Dart et al,^65^ 2014	Canada	RC	13.5 (2.2)	13.5 (2.2)	0	93 (27.1)	342	M: 129 (37.8); F: 213 (62.2)	NR	NR	ACR >3mg/mmol or AER >30mg/24 h on ≥2 of 3 measurements 1 mo apart	NR
Eppens et al,^4^ 2006	Australia	PC	13.2 (11.6-15.0)^a^^,^^b^	15.3 (13.6-16.4)^a^^,^^b^	1.3 (0.6-3.1)^a^^,^^b^	10 (28)	36	NR	NR	NR	AER ≥20 μg/min in ≥2 of 3 samples or ACR ≥2.5 mg/mmol	36 (56)^a^
Jefferies et al,^72^ 2012	New Zealand	PC	mean, 12.9; range, 7.1-15.5	NR	NR	18 (35)	52	M: 17 (33); F: 35 (67)	PI/Maori: 47 (90); other: 5 (10)	NR	ACR ≥2.5 mg/mmol on ≥2 of 3 samples during 6-mo period	NR
Dart et al,^74^ 2019	Canada	PC	All participants <18	15 (13.3-16.8^b^	2.3 (0.9-4.1)^b^	57 (30.5)	187	M: 62 (33.2); F: 125 (66.8)	Indigenous: 179 (95.7); other: 8 (4.3)	M: 15 (24); F: 42 (33.6); Indigenous: 56 (31.3)	ACR >2mg/mmol on 2 of 3 samples during a 6-mo period	NR
**Microalbuminuria**												
Hotu et al,^78^ 2004	New Zealand	CS	mean, 15 (11-19	NR	NR	5 (42)	12	M: 6 (50); F: 6 (50)	Maori/PI: 12 (100)	M: 3 (50); F: 2 (33); Maori/PI: 5 (42)	ACR ≥30-300mg/g	NR
Ettinger et al,^42^ 2005	United States	CS	NR	15.0 (1.9)	1.5 (1.0)	10 (40)	25	M: 12 (46); F: 14 (54)^a^	H: 15 (58); NHB: 8 (31); other: 2 (7); multiracial: 1 (4)^a^	NR	AER ≥30mg albumin/24 h	NR
Eppens et al,^44^ 2006	Western Pacific	CS	12.0 (10.7-13.5)^a^^,^^b^	14.9 (13.2-16.4)^a^^,^^b^	2.3 (1.4-3.6)^a^^,^^b^	20 (8.0)	251	NR	NR	NR	AER 30-300mg/24 h or >20 μg/min or ACR >2.5mg/mmol	106 (32.0)^a^
Kim et al,^83^ 2010	United States	CS	NR	14.5 (3.0)	mean, 1.3 (0-2.1	19 (18.5)	103	M: 40 (38.8); F: 63 (61.2)	Pima Indian: 103 (100.0)	Pima Indian: 19 (18.5)	ACR ≥30-300mg/g	NR
Amutha et al,^53^ 2012	India	CS	16.1 (2.5)	22.2 (9.7)	Age ≤5 y: 219 (59.5%); age >5 to ≤10 y: 67 (18.2%); age >10 to ≤15 y: 21 (5.7%); age >15 y: 61 (16.6%)	54 (14.7)	368	M: 168 (45.7); F: 200 (54.3)	South Indian: 368 (100)^c^	South Indian: 54 (14.7)	ACR ≥30-299mg/g	NR
Zabeen et al,^89^ 2016^e^	Bangladesh	CS	13.0 (11.0-15.0)^d^	13.0 (11.0-15.0)^d^	0	14 (10.0)	144	NR	Bangladeshi: 144 (100)^c^	Bangladeshi: 14 (10.0)	ACR ≥30-300mg/g	NR
Nambam et al,^90^ 2017	United States	CS	All participants <21	16.0 (14.0-17.7)^b^	2.0 (0.7-4.2)^b^	36 (6.0)	598	M: 218 (36.5); F: 380 (63.5)	H: 329 (55.0); AA: 179 (30.0); NHW: 48 (8.0); other/multiracial: 42 (7.0)	NR	NR	472 (85.0)^a^
Le et al,^91^ 2013	United States	RC	13.8 (2.4)^a^	14.2 (2.4)	NR	11 (17)	64	M: 20 (31); F: 44 (69)	AA: 52 (81); NHW: 12 (19)	AA: 11 (21); NHW: 0^f^	ACR ≥30-299 mg/g	NR
Osman et al,^64^ 2013	Sudan	RC	Age <11 y: 3 (7.9%); age 11-18 y: 35 (92.1%)	NR	NR	7 (18)	38	M: 17 (45); F: 21 (55)	Arab: 32 (84); multiracial: 4 (11); non-Arab: 2 (5)	NR	NR	29 (76)
Haynes et al,^66^ 2014^e^	Australia	RC	13.3 (2.0)^a^	13.3 (2.0)^a^	0	11 (18)	61	NR	NR	NR	NR	82 (60.7)^a^
Calagua Quispe et al,^92^ 2015^e^	Peru	RC	12.6 (2.3)	NR	3.7 (2.4)	9 (43)	20	M: 10 (50); F: 10 (50)	NR	NR	NR	NR
Newton et al,^93^ 2015^e^	New Zealand	RC	range, 6.5-17	All participants, <17	NR	6 (55)	11	NR	NR	NR	NR	22 (96)^a^
Son et al,^94^ 2015	Korea	RC	NR	15.4 (12.6-17.4)^b^	0.9 (0.0-3.0)^b^	8 (44)	18	M: 4 (22); F: 14 (78)	Korean: 18 (100)^c^	Korean: 8 (44)	ACR ≥30-300mg/g	NR
Yeow et al.,^68^ 2019	Malaysia	RC	14.3 (3.5)	20.7 (3.7)	6.5 (2.8)	7 (29)	24	M: 10 (42); F: 14 (58)	Malay: 12 (50); Chinese: 11 (46); Asian Indian: 1 (4)	Asian: 7 (29)	M: ACR >2.5mg/mmol, F: ACR >3.5mg/mmol	10 (42)
Ruhayel et al,^71^ 2010	Australia	PC	13.4 (9.2-17.4)^a^^,^^d^	M: 16.0 (13.6-18.2); F: 15.6 (11.7-19.8)^a^^,^^d^	M: 2.2 (0.0-7.8); F: 2.3 (0.1-7.4)^a^^,^^d^	9 (45)	20	NR	NR	NR	ACR >3.5mg/mmol	23 (70)^a^
Schmidt et al,^73^ 2012	Germany and Austria	PC	13.5 (3.3)	15.3 (3.0)	NR	154 (22.5)	684	M: 261 (38.2); F: 423 (61.8)	German/Austrian: 482 (70.5); other: 202 (29.5)	NR	NR	NR
**Persistent microalbuminuria**												
Yoo et al,^86^ 2004	Korea	CS	12.8 (1.5)	18.4 (4.3)	5.5 (3.9)	4 (18)	22	M: 8 (36); M: 14 (64)	Korean: 22 (100)	Korean: 4 (18)	AER >20 μg/min on samples 3 mo apart	NR
Farah et al,^87^ 2006	United States	CS	NR	range, 10-21	mean, 1.8; range, <2-5	9 (27)	33	NR	NR	NR	ACR >30mg/g on 2 samples within 3-6 mo	29 (73)^a^
Copeland et al,^15^ 2011	United States	CS	range, 10-17	14.0 (2.0)	0.7 (0.5)	92 (13.0)	704	M: 247 (35.1); F: 457 (64.9)	H: 289 (41.1); NHB: 222 (31.5); NHW: 138 (19.6); AI: 43 (6.1); Asian: 12 (1.7)	M: 35 (14.3); F: 48 (10.6); H: 41 (14.1); NHB: 25 (11.2); NHW: 20 (14.6); AI: 1 (8)	ACR ≥30mg/g on 2 of 3 samples during 3-mo period	NR
Aulich et al,^57^ 2019	Australia	CS	NR	15.1 (1.9)	1.8 (0.3-3.3)^b^	6 (30)	20	NR	NR	NR	AER ≥20 μg/min in ≥2 of 3 samples or mean ACR, M: ≥3.5 mg/mmol; F: ≥4 mg/mmol from 3 first morning collections	24 (75)^a^
Scott et al,^59^ 2004	New Zealand	RC	NR	19.6 range, 14-23	1.7	2 (15)	13	M: 7 (54); F: 6 (46)	Maori: 7 (54); European: 4 (30); PI: 1 (8); Asian Indian: 1 (8)	NR	M: ACR >2.5mg/mmol, F: ACR >3.5mg/mmol on ≥2 occasions	13 (100)
Scott et al,^62^ 2006	New Zealand	RC	NR	20.0 (0.4)	3.0 (0.3)	76 (72.4)	105	NR	Maori/PI/other: 66 (62.9); European: 39 (37.1)	NR	M: ACR >2.5mg/mmol, F: ACR >3.5mg/mmol on ≥2 occasions	105 (100)
Dart et al,^8^ 2012	Canada	RC	13.5 (2.2)	14.9 (2.1)	1.6 (1.5)	92 (26.9)	342	M: 129 (37.8); F: 213 (62.2)	NR	NR	ACR ≥3mg/mmol or AER 30mg/24 h on 2 of 3 samples 1 mo apart	NR
Son et al,^94^ 2015	Korea	RC	NR	15.4 (12.6-17.4)^b^	0.9 (0.0-3.0)^b^	5 (28)	18	M: 4 (22); F: 14 (78)	Korean: 18 (100)^c^	Korean: 5 (28)	ACR ≥30-300 mg/g at baseline and follow-up	NR
Eppens et al,^4^ 2006	Australia	PC	13.2 (11.6-15.0)^a^^,^^b^	15.3 (13.6-16.4)^a^^,^^b^	1.3 (0.6-3.1)^a^^,^^b^	10 (28)	36	NR	NR	NR	AER ≥20 μg/min in at least 2 of 3 samples or ACR ≥2.5 mg/mmol	36 (56)^a^
Jefferies et al,^72^ 2012	New Zealand	PC	mean, 12.9; range, 7.1-15.5	NR	NR	18 (35)	52	M: 17 (33); F: 35 (67)	PI/Maori: 47 (90); other: 5 (10)	NR	ACR ≥2.5 mg/mmol on ≥2 of 3 samples during 6-mo period	NR
**Macroalbuminuria**												
Hotu et al,^78^ 2004	New Zealand	CS	mean, 15; range, 11-19	NR	NR	2 (17)	12	M: 6 (50); F: 6 (50)	Maori/PI: 12 (100)	M: 1 (17); F: 1 (17); Maori/PI: 2 (17)	ACR >300mg/g	NR
Eppens et al,^44^ 2006	Western Pacific	CS	12.0 (10.7-13.5)^a^^,^^b^	14.9 (13.2-16.4)^a^^,^^b^	2.3 (1.4-3.6)^a^^,^^b^	1 (0.6)	247	NR	NR	NR	AER >300mg/24 h	106 (32.0)^a^
Kim et al,^83^ 2010	United States	CS	NR	14.5 (3.0)	1.3 (0-2.1)^b^	3 (2.9)	103	M: 40 (38.8); F: 63 (61.2)	Pima Indian: 103 (100)	Pima Indians: 3 (2.9)	ACR >300mg/g	NR
Amutha et al,^53^ 2012	India	CS	16.1 (2.5)	22.2 (9.7)	Age ≤5 y: 219 (59.5%); age >5 to ≤10 y: 67 (18.2%); age >10 to ≤15: 21 (5.7%); age >15 y: 61 (16.6%)	31 (8.4)	368	NR	South Indian: 368 (100)^c^	South Indian: 31 (8.4)	ACR >300mg/g	NR
Schmidt et al,^73^ 2012	Germany and Austria	PC	13.5 (3.4)	15.3 (3.0)	NR	16 (2.4)	684	M: 261 (38.2); F: 423 (61.8)	German/Austrian: 482 (70.5); other: 202 (29.5)	NR	NR	NR
**Persistent macroalbuminuria**												
Yoo et al,^86^ 2004	Korea	CS	12.8 (1.5)	18.4 (4.3)	5.5 (3.9)	1 (5)	22	M: 8 (36); F: 14 (64)	Korean: 22 (100)	Korean: 1 (5)	AER >200 μg/min on samples 3 mo apart	NR
Dart et al,^8^ 2012	Canada	RC	13.5 (2.2)	14.9 (2.1)	1.6 (1.5)	16 (4.7)	342	M: 129 (37.8); F: 213 (62.2)	NR	NR	NR	NR

Abbreviations: AA, African American; ACR, albumin-to-creatinine ratio; AER, albumin excretion rate; AI, American Indian; API, Asian–Pacific Islander; BACBB, Black/African/Caribbean/Black British; CS, cross-sectional; F, Females; FN, First Nations; H, Hispanic; NR, not reported; M, Males; NHB, Non-Hispanic Black; NHW, Non-Hispanic White; PC, prospective cohort; PI, Pacific Islander; RC, retrospective cohort.

^a^ Based on total study cohort instead of only patients examined for the specific comorbidity.

^b^ Median (interquartile range).

^c^ Racial group distribution assumed to match country of origin.

^d^ Median (range).

^e^ Abstract only.

^f^ Value estimated based on graph.

#### Pooled Prevalence of Albuminuria and Persistent Albuminuria in Type 2 Diabetes

**Figure 2.**
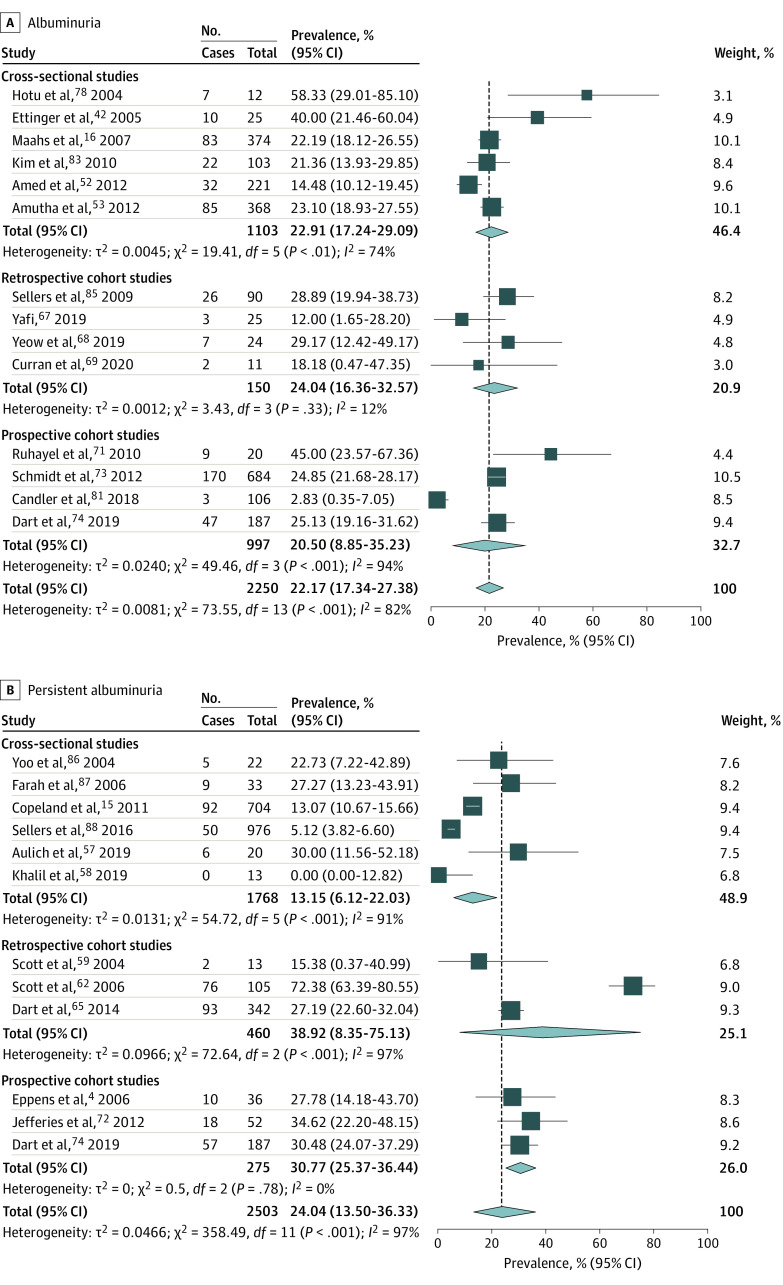
Forest Plot Showing Pooled Prevalence of Albuminuria and Persistent Albuminuria in Pediatric Type 2 Diabetes

Pooled albuminuria prevalence in 14 studies of 2250 patients with type 2 diabetes was 22.17% (95% CI, 17.34%-27.38%) (Figure 2).^16,42,52,53,67,68,69,71,73,74,78,81,83,85^ There were high levels of heterogeneity (*I*^2^ = 82%; *P* < .001).

Four studies were not included in the meta-analysis. One used a definition of albuminuria of 24-hour urine protein excretion of greater than 500 mg, and found no patients with this outcome.^45^ Another study did not report the sample size or the definition of albuminuria but reported a prevalence of 23%.^84^ Two other studies^46,50^ reported the prevalence in specific racial groups, and the data were captured by another included study.^16^ Removing studies with 678 patients older than 18 years lowered the estimate to 17.00% (95% CI, 9.00%-27.00%; *I*^2^ = 86%; *P* < .001),^42,52,67,69,74,81,83^ suggesting that albuminuria worsens with age in this population (eTable 7 in the Supplement).

Pooled prevalence of persistent albuminuria across 12 studies with 2503 participants was 24.04% (95% CI, 13.50%-36.33%; *I*^2^ = 97%; *P* < .001) (Figure 2).^4,15,57,58,59,62,65,72,74,86,87,88^ Removing studies using different definitions of albuminuria lowered the pooled estimate to 17.00% (95% CI, 7.00%-29.00%; *I*^2^ = 92%; *P* < .001) (eTable 8 in the Supplement).

Microalbuminuria pooled prevalence across 16 studies with 2441 participants was 21.57% (95% CI, 15.59%-28.16%; *I*^2^ = 90%; *P* < .001) (eFigure 7 in the Supplement).^42,44,53,64,66,68,71,73,78,83,89,90,91,92,93,94^ Removing studies with 50 participants or fewer changed the estimate to 14.00% (95% CI, 9.00%-20.00%; *I*^2^ = 92%; *P* < .001; 2273 participants),^44,53,66,73,83,89,90,91^ as this resulted in the elimination of Pacific Islander group from the analysis; this group had the highest microalbuminuria prevalence (eTable 9 in the Supplement). Ten studies with 1345 participants reported persistent microalbuminuria pooled prevalence of 29.19% (95% CI, 16.85%-43.21%; *I*^2^ = 95%; *P* < .001) (eFigure 8 in the Supplement).^4,8,15,57,59,62,72,86,87,94^ Similarly, removing studies with patients older than 18 years or those using different definitions of microalbuminuria reduced the prevalence and heterogeneity estimates to 23.00% (95% CI, 14.00%-34.00%; *I*^2^ = 88%; *P* < .001) and 24.00% (95% CI, 11.00%-39.00%; *I*^2^ = 84%; *P* < .001), respectively, because this resulted in the exclusion of a study reporting a very high microalbuminuria prevalence of 72% among 105 participants in a sample with a high proportion of Pacific Islander and Indigenous patients (eTable 10 in the Supplement).^62^

Macroalbuminuria pooled prevalence from 4 studies with 730 participants was 3.85% (95% CI, 0.02%-11.63%; *P* < .001) (eFigure 9 in the Supplement).^44,53,78,83^ Another study reported a prevalence of 2.4% among 684 participants,^73^ but it was the only prospective cohort study, so it was not included in the meta-analysis. In addition, the prevalence of persistent macroalbuminuria was 5% among 22 participants in 1 cross-sectional study^86^ and 4.7% among 342 participants in another retrospective cohort study.^8^ Metaregression analysis revealed no statistically significant correlation between obesity prevalence and albuminuria, persistent albuminuria, microalbuminuria, or persistent microalbuminuria prevalence.

#### Sex and Racial Group Associations With Albuminuria

One study reported that albuminuria in 140 male participants (20.7%) was lower than in 234 female participants (23.1%).^16^ Similarly, persistent microalbuminuria prevalence was higher in 247 male participants (14.3%) than in 457 female participants (10.6%) in another study.^15^ Persistent albuminuria prevalence across 2 studies in 309 male participants was 16.14% (95% CI, 5.05%-31.65%; *I*^2^ = 86%; *P* < .001) and 22.90% (95% CI, 7.08%-44.22%; *I*^2^ = 95%; *P* < .001) in 582 female participants (OR, 0.68 [95% CI, 0.46-1.01]; *I*^2^ = 0%; *P *for heterogeneity = .78) (eFigure 10 and eFigure 11 in the Supplement).^15,74^

Albuminuria prevalence was assessed by racial group, and White youth had lower rates of albuminuria than other groups. The pooled prevalence among 158 White participants was 12.59% (95% CI, 7.75%-18.33%; *I*^2^ = 0%; *P* = .58)^46,52^ compared to 23.00% (95% CI, 18.85%-27.41%; *I*^2^ = 0%; *P* = .46) in 392 Asian participants,^53,68^ 24.27% (95% CI, 14.39%-35.73%; *I*^2^ = 79%; *P* < .01) in 295 Indigenous participants,^16,52,83^ and 31.84% (95% CI, 11.90%-55.47%; *I*^2^ = 58%; *P* = .09) in 48 Pacific Islander participants^16,69,78^ (eFigure 12 in the Supplement). Single studies found an albuminuria prevalence of 14.1% in 212 African American participants^50^ and 23% in 64 Hispanic/Latino participants.^16^

Individual studies reported a prevalence of persistent albuminuria of 11.2% in 222 African American participants,^15^ 14.1% in 289 Hispanic/Latino participants,^15^ and 23% in 22 Korean participants.^86^ Two studies reported a prevalence of 6.96% (95% CI, 0.00%-25.91%; *I*^2^ = 70%; *P* = .07) in 150 White participants^15,58^ and 19.06% (95% CI, 2.27%-45.67%; *I*^2^ = 91%; *P* < .001) in 217 Indigenous participants (eFigure 13 in the Supplement).^15,74^

For microalbuminuria, the pooled prevalence was 18.98% (95% CI, 9.98%-29.87%; *I*^2^ = 80%; *P* = .002) in 554 Asian participants (eFigure 14 in the Supplement).^53,68,89,94^ Individual studies found a prevalence of microalbuminuria of 0% in 12 White participants,^91^ 21% in 52 African American participants,^91^ 18.5% in 103 Indigenous participants,^83^ and 42% in 12 Pacific Islander participants.^78^

Finally, for persistent microalbuminuria, the pooled prevalence 22.31% (95% CI, 10.22%-37.01%; *I*^2^ = 0%; *P* = .48) in 40 Asian participants (eFigure 15 in the Supplement).^86,94^ One study reported a prevalence of 14.1% in 289 Hispanic/Latino participants, 11.2% in 222 African American participants, 14.6% in 138 White participants, and 8% in 43 Indigenous participants.^15^

### Publication Bias

Publication bias was found for the prevalence of microalbuminuria based on the funnel plot and Egger test. It was not found for hypertension, albuminuria, persistent albuminuria, or persistent microalbuminuria (eFigures 16-20 in the Supplement).

### Risk of Bias and Overall Quality of Evidence

The included studies had either a low (n = 27)^8,15,16,17,40,44,45,46,47,48,50,53,55,56,60,64,65,69,70,72,76,78,79,81,85,89,90^ or moderate (n = 33)^4,41,42,43,49,51,52,54,57,58,59,61,62,63,66,67,68,71,73,74,75,77,80,82,83,84,86,87,88,91,92,93,94^ risk of bias (eTable 11 and eFigure 21 in the Supplement). Some studies did not have a nationally representative sample, which limits their generalizability.^4,8,40,41,42,43,46,47,48,49,50,51,53,54,56,57,58,59,60,63,64,65,66,67,68,71,72,74,75,77,78,79,80,82,83,85,86,87,89,91,92,93,94^ The sampling frame of some studies was not representative of their target population,^41,42,44,51,52,54,61,63,67,74,75,77,80,82,86,87,88,91,92,94^ and some did not take a random or census sample.^15,41,42,51,52,54,57,67,75,82,86,87,92,94^ Some studies also had missing data of greater than 25%, potentially leading to nonresponse bias.^4,43,49,57,58,61,62,66,68,71,73,75,83,84,91,93^ In 3 studies, the definition used to diagnose hypertension or albuminuria was unspecified,^67,69,92^ and in some studies it was unclear that all participants were examined using the same methods.^45,52,55,59,61,62,70,73,81,84,88,90^

Based on the OCEBM criteria,^28^ 29 studies (48%)^8,16,17,40,44,46,47,48,50,53,55,56,62,65,66,70,72,73,74,76,79,80,81,83,85,88,89,90,91^ had an evidence level of 1; 17 studies (28%)^4,43,45,49,58,59,60,61,63,64,68,69,71,77,78,84,93^, 2; and 14 studies (23%),^15,41,42,51,52,54,57,67,75,82,86,87,92,94^ 3 (eTable 11 in the Supplement). Nearly half of the studies thus provide the highest level of evidence to answer the prevalence question we posed, although a significant portion of studies did not use a random sample or census to estimate prevalence.

## Discussion

The rates of type 2 diabetes in children and adolescents are increasing globally, and this rise is associated with the obesity epidemic.^95^ Type 2 diabetes is associated with rapid progression of kidney complications, and early detection and treatment are crucial to avoid end-stage kidney disease, cardiovascular morbidities, and mortality.^7,8,11,14^ Current clinical guidelines on the management of pediatric type 2 diabetes are informed by independent studies with variable sample sizes.^9,12,13^

This systematic review investigated the prevalence of hypertension and albuminuria, markers of diabetes-related nephropathy and important predictors of kidney outcomes, in pediatric type 2 diabetes. Approximately 1 in 4 pediatric patients with type 2 diabetes had hypertension. Although more female patients had type 2 diabetes than male patients,^9^ male patients appeared to be more likely to develop hypertension than female patients. Pacific Islander and Indigenous youth had a higher burden of hypertension than other racial groups.

While most studies followed the National Heart, Lung, and Blood Institute guidelines for assessing hypertension in children and adolescents^4,17,42,44,46,47,48,49,50,56,57,60,68,73,74^ (ie, BP ≥95th percentile for age, sex, and height^20^), 6 studies used the adult definition of hypertension of systolic BP level of 130 to 140 mm Hg or greater and diastolic BP level of 80 to 90 mm Hg or greater.^51,53,58,59,62,63^ The different definitions of hypertension across studies were partly responsible for the noted heterogeneity, and using adult definitions might underestimate hypertension prevalence in children and adolescents.

Updated National Heart, Lung, and Blood Institute guidelines were released in 2017 that lowered BP thresholds for hypertension, as they were based on only children with weight in the reference range, whereas older guidelines also included children with overweight and obesity.^96^ While no studies reported using the 2017 guidelines, it is possible that hypertension prevalence will be higher if assessing data from existing studies against the 2017 guidelines.

Obesity is an important contributor to hypertension risk, with an estimated 6% increased risk of hypertension per unit of body mass index increase.^6^ Conversely, the metaregression analysis revealed that obesity was not associated with the prevalence of hypertension. However, obesity prevalence and severity were not available in all studies, and it is probable that obesity may contribute indirectly to the risk of hypertension in pediatric type 2 diabetes. On a mechanistic level, obesity-driven insulin resistance and hyperinsulinemia increases sodium reabsorption from the renal tubules.^97^ In addition, hyperglycemia can lead to hypervolemia, increased sympathetic activity,^97^ and the activation of the renin-angiotensin-aldosterone system, which increases cardiac output and peripheral vascular resistance, leading to hypertension.^97^ The associations between obesity and hypertension in pediatric type 2 diabetes require further study.

This review also demonstrated that between 1 in 5 and 1 in 4 pediatric patients with type 2 diabetes had albuminuria. While no sex differences were identified, Pacific Islander, Indigenous, and Asian youth had higher rates of albuminuria than White youth. While macroalbuminuria occurred in 4% of participants, fewer studies reported the persistence of albuminuria, despite the need for confirmation of persistence being a key criterion for albuminuria diagnosis.^9,12,13^ Persistent albuminuria is associated with macrovascular disease^98^ and predicts the progression to end-stage kidney disease.^9,99^ Prospective studies are needed to assess persistent albuminuria in pediatric type 2 diabetes. When studies of adult patients with type 2 diabetes were excluded, the albuminuria pooled prevalence estimate decreased from 22.17% (95% CI, 17.34%-27.38%) to 17.00% (95% CI, 9.00%-27.00%), and these results corroborate current evidence that albuminuria increases with age and duration of diabetes.^6,53^

These data have several important implications. Type 2 diabetes–related nephropathy exerts a much higher burden than that seen in children with type 1 diabetes.^16,100^ For example, the SEARCH for Diabetes in Youth study reported elevated urine ACR of 9.2% in children with type 1 diabetes vs 22.2% in those with type 2 diabetes,^16^ and youth with type 2 diabetes had 4-fold higher rates of kidney failure compared with youth with type 1 diabetes.^8^ In addition, a study including Pima Indians, an Indigenous group with high rates of type 2 diabetes, found that those who developed type 2 diabetes before 20 years of age had a 5-fold increased risk of end-stage kidney disease by middle age and higher mortality rates compared with patients with adult-onset type 2 diabetes.^7^ Ongoing intensive screening and intervention strategies are warranted to reduce mortality and end-stage kidney disease in pediatric patients with type 2 diabetes.

The specific renal pathology that drives proteinuria and hypertension in pediatric type 2 diabetes is unknown. While studies of kidney biopsies in youth with type 2 diabetes are limited, most anomalies found on kidney ultrasounds have been classified as congenital.^88^ Kidney biopsies from patients describe immune complex disease and glomerulosclerosis, findings that are not characteristic of typical diabetes-related nephropathy, which are considered non–diabetes-driven pathologies.^85^ However, these observations are based on a small sample of Indigenous youth and may not be generalizable to all children and adolescents with type 2 diabetes. Further studies are urgently needed to assess renal histopathology in type 2 diabetes across different sexes and racial groups to define the exact mechanisms of nephropathy in this population.

### Limitations

This study has limitations, including the high heterogeneity among studies. Some studies did not achieve a high quality rating (n = 31) because of small sample sizes (n = 17) or the lack of clarity as to whether the results were based on a randomized sample or census (n = 14). Moreover, a large proportion of the studies did not have a nationally representative sample, as they were based in a single center or clinic. As such, larger studies across multiple centers are needed to assess prevalence. In addition, obesity severity data, which could confound hypertension and proteinuria prevalence, were also not available. While the results should be interpreted with this information in mind, this report presents all current data available to assess hypertension and albuminuria in pediatric patients with type 2 diabetes.

## Conclusions

In this study, hypertension and albuminuria were frequent comorbidities of pediatric type 2 diabetes, and Pacific Islander and Indigenous youth had a disproportionately higher burden of these conditions than youth from other racial groups. There is a critical need for personalized screening and treatment strategies to provide renoprotection from prolonged hyperglycemia and obesity to prevent end-stage kidney disease, future cardiovascular disease, and improve life expectancy. The exact etiopathogenetic mechanisms driving nephropathy in youth with type 2 diabetes need to be elucidated. These data are relevant for health care professionals and policy makers, as clinical services treating pediatric patients with type 2 diabetes need to be resourced to track kidney screening and treatments to improve outcomes.

## Notes

Supplement. **eTable 1.** Search Strategy, MEDLINE **eTable 2.** Search Strategy, Embase **eTable 3.** Search Strategy, CINAHL **eTable 4.** Search Strategy, Cochrane Library: Cochrane Central Register of Controlled Trials and Cochrane Database of Systematic Reviews **eTable 5.** Search Strategy, Web of Science: Conference Proceedings Citation Index-Science **eTable 6.** Results of Sensitivity Analysis for Prevalence of Hypertension in Pediatric Type 2 Diabetes Meta-analysis **eTable 7.** Results of Sensitivity Analysis for Prevalence of Albuminuria in Pediatric Type 2 Diabetes Meta-analysis **eTable 8.** Results of Sensitivity Analysis for Prevalence of Persistent Albuminuria in Pediatric Type 2 Diabetes Meta-analysis **eTable 9.** Results of Sensitivity Analysis for Prevalence of Microalbuminuria in Pediatric Type 2 Diabetes Meta-analysis **eTable 10.** Results of Sensitivity Analysis for Prevalence of Persistent Microalbuminuria in Pediatric Type 2 Diabetes Meta-analysis **eTable 11.** Risk of Bias and OCEBM Level of Evidence of Included Studies **eFigure 1.** Study Flow Diagram **eFigure 2.** Forest Plot Showing Pooled Prevalence of Systolic Hypertension in Pediatric Type 2 Diabetes **eFigure 3.** Forest Plot Showing Pooled Prevalence of Diastolic Hypertension in Pediatric Type 2 Diabetes **eFigure 4.** Forest Plot Showing Pooled Prevalence of Hypertension in Pediatric Type 2 Diabetes by Sex **eFigure 5.** Forest Plot Showing Pooled Odds Ratio of Hypertension in Male vs Female Participants with Pediatric Type 2 Diabetes **eFigure 6.** Forest Plot Showing Pooled Prevalence of Hypertension Across Different Racial Groups with Pediatric Type 2 Diabetes **eFigure 7.** Forest Plot Showing Pooled Prevalence of Microalbuminuria in Pediatric Type 2 Diabetes **eFigure 8.** Forest Plot Showing Pooled Prevalence of Persistent Microalbuminuria in Pediatric Type 2 Diabetes **eFigure 9.** Forest Plot Showing Pooled Prevalence of Macroalbuminuria in Pediatric Type 2 Diabetes **eFigure 10.** Forest Plot Showing Pooled Prevalence of Persistent Albuminuria in Pediatric Type 2 Diabetes by Sex **eFigure 11.** Forest Plot Showing Pooled Odds Ratio of Persistent Albuminuria in Male vs Female Participants with Pediatric Type 2 Diabetes **eFigure 12.** Forest Plot Showing Pooled Prevalence of Albuminuria across Different Racial Groups with Pediatric Type 2 Diabetes **eFigure 13.** Forest Plot Showing Pooled Prevalence of Persistent Albuminuria across Different Racial Groups with Pediatric Type 2 Diabetes **eFigure 14.** Forest Plot Showing Pooled Prevalence of Microalbuminuria in Asian Patients with Pediatric Type 2 Diabetes **eFigure 15.** Forest Plot Showing Pooled Prevalence of Persistent Microalbuminuria in Asian Patients with Pediatric Type 2 Diabetes **eFigure 16.** Funnel Plot Examining Publication Bias for Pooled Prevalence of Hypertension Outcome **eFigure 17.** Funnel Plot Examining Publication Bias for Pooled Prevalence of Albuminuria Outcome **eFigure 18.** Funnel Plot Examining Publication Bias for Pooled Prevalence of Persistent Albuminuria Outcome **eFigure 19.** Funnel Plot Examining Publication Bias for Pooled Prevalence of Microalbuminuria Outcome **eFigure 20.** Funnel Plot Examining Publication Bias for Pooled Prevalence of Persistent Microalbuminuria Outcome **eFigure 21.** Distribution of Risk of Bias Sources in the Included Studies **eAppendix.** List of Studies Excluded at the Full-Text Screening Stage **eReferences.**
