# Error in Abstract

**DOI:** 10.1001/jamanetworkopen.2023.41796

**Published:** 2023-10-19

In the Original Investigation titled “Prevalence of Hypertension and Albuminuria in Pediatric Type 2 Diabetes: A Systematic Review and Meta-analysis,”^1^ published April 30, 2021, there was an error in the Abstract. The number of participants in the analysis of hypertension prevalence should have read 4363. This article has been corrected.^1^
