# Supplementary material for: Prevalence of Hypertension and Albuminuria in Pediatric Type 2 Diabetes: A Systematic Review and Meta-analysis
Source: JAMA Netw Open. 2021 Apr 30;4(4):e216069. doi: 10.1001/jamanetworkopen.2021.6069 (PMC8087958; doi:10.1001/jamanetworkopen.2021.6069)
Supplement: Supplement. — eTable 1. Search Strategy, MEDLINE eTable 2. Search Strategy, Embase eTable 3. Search Strategy, CINAHL eTable 4. Search Strategy, Cochrane Library: Cochrane Central Register of Controlled Trials and Cochrane Database of Systematic Reviews eTable 5. Search Strategy, Web of Science: Conference Proceedings Citation Index-Science eTable 6. Results of Sensitivity Analysis for Prevalence of Hypertension in Pediatric Type 2 Diabetes Meta-analysis eTable 7. Results of Sensitivity Analysis for Prevalence of Albuminuria in Pediatric Type 2 Diabetes Meta-analysis eTable 8. Results of Sensitivity Analysis for Prevalence of Persistent Albuminuria in Pediatric Type 2 Diabetes Meta-analysis eTable 9. Results of Sensitivity Analysis for Prevalence of Microalbuminuria in Pediatric Type 2 Diabetes Meta-analysis eTable 10. Results of Sensitivity Analysis for Prevalence of Persistent Microalbuminuria in Pediatric Type 2 Diabetes Meta-analysis eTable 11. Risk of Bias and OCEBM Level of Evidence of Included Studies eFigure 1. Study Flow Diagram eFigure 2. Forest Plot Showing Pooled Prevalence of Systolic Hypertension in Pediatric Type 2 Diabetes eFigure 3. Forest Plot Showing Pooled Prevalence of Diastolic Hypertension in Pediatric Type 2 Diabetes eFigure 4. Forest Plot Showing Pooled Prevalence of Hypertension in Pediatric Type 2 Diabetes by Sex eFigure 5. Forest Plot Showing Pooled Odds Ratio of Hypertension in Male vs Female Participants with Pediatric Type 2 Diabetes eFigure 6. Forest Plot Showing Pooled Prevalence of Hypertension Across Different Racial Groups with Pediatric Type 2 Diabetes eFigure 7. Forest Plot Showing Pooled Prevalence of Microalbuminuria in Pediatric Type 2 Diabetes eFigure 8. Forest Plot Showing Pooled Prevalence of Persistent Microalbuminuria in Pediatric Type 2 Diabetes eFigure 9. Forest Plot Showing Pooled Prevalence of Macroalbuminuria in Pediatric Type 2 Diabetes eFigure 10. Forest Plot Showing Pooled Prevalence of Persistent Albuminuria in Pediatric Type [file jamanetwopen-e216069-s001.pdf]

Supplemental Online Content

Cioana M, Deng J, Hou M, et al. Prevalence of hypertension and albuminuria in pediatric type 2 diabetes: a systematic review and meta-analysis. *JAMA Netw Open.* 2021;4(4):e216069. doi:10.1001/jamanetworkopen.2021.6069

eTable 1. Search Strategy, MEDLINE

eTable 2. Search Strategy, Embase

eTable 3. Search Strategy, CINAHL

eTable 4. Search Strategy, Cochrane Library: Cochrane Central Register of Controlled Trials and Cochrane Database of Systematic Reviews

eTable 5. Search Strategy, Web of Science: Conference Proceedings Citation Index-Science

eTable 6. Results of Sensitivity Analysis for Prevalence of Hypertension in Pediatric Type 2 Diabetes Meta-analysis

eTable 7. Results of Sensitivity Analysis for Prevalence of Albuminuria in Pediatric Type 2 Diabetes Meta-analysis

eTable 8. Results of Sensitivity Analysis for Prevalence of Persistent Albuminuria in Pediatric Type 2 Diabetes Meta-analysis

eTable 9. Results of Sensitivity Analysis for Prevalence of Microalbuminuria in Pediatric Type 2 Diabetes Meta-analysis

eTable 10. Results of Sensitivity Analysis for Prevalence of Persistent Microalbuminuria in Pediatric Type 2 Diabetes Meta-analysis

eTable 11. Risk of Bias and OCEBM Level of Evidence of Included Studies

eFigure 1. Study Flow Diagram

eFigure 2. Forest Plot Showing Pooled Prevalence of Systolic Hypertension in Pediatric Type 2 Diabetes

eFigure 3. Forest Plot Showing Pooled Prevalence of Diastolic Hypertension in Pediatric Type 2 Diabetes

eFigure 4. Forest Plot Showing Pooled Prevalence of Hypertension in Pediatric Type 2 Diabetes by Sex

eFigure 5. Forest Plot Showing Pooled Odds Ratio of Hypertension in Male vs Female Participants With Pediatric Type 2 Diabetes

© 2021 Cioana M et al. *JAMA Network Open.*

eFigure 6. Forest Plot Showing Pooled Prevalence of Hypertension Across Different Racial Groups With Pediatric Type 2 Diabetes

eFigure 7. Forest Plot Showing Pooled Prevalence of Microalbuminuria in Pediatric Type 2 Diabetes

eFigure 8. Forest Plot Showing Pooled Prevalence of Persistent Microalbuminuria in Pediatric Type 2 Diabetes

eFigure 9. Forest Plot Showing Pooled Prevalence of Macroalbuminuria in Pediatric Type 2 Diabetes

eFigure 10. Forest Plot Showing Pooled Prevalence of Persistent Albuminuria in Pediatric Type 2 Diabetes by Sex

eFigure 11. Forest Plot Showing Pooled Odds Ratio of Persistent Albuminuria in Male vs Female Participants With Pediatric Type 2 Diabetes

eFigure 12. Forest Plot Showing Pooled Prevalence of Albuminuria Across Different Racial Groups With Pediatric Type 2 Diabetes

eFigure 13. Forest Plot Showing Pooled Prevalence of Persistent Albuminuria Across Different Racial Groups With Pediatric Type 2 Diabetes

eFigure 14. Forest Plot Showing Pooled Prevalence of Microalbuminuria in Asian Patients with Pediatric Type 2 Diabetes

eFigure 15. Forest Plot Showing Pooled Prevalence of Persistent Microalbuminuria in Asian Patients with Pediatric Type 2 Diabetes

eFigure 16. Funnel Plot Examining Publication Bias for Pooled Prevalence of Hypertension Outcome

eFigure 17. Funnel Plot Examining Publication Bias for Pooled Prevalence of Albuminuria Outcome

eFigure 18. Funnel Plot Examining Publication Bias for Pooled Prevalence of Persistent Albuminuria Outcome

eFigure 19. Funnel Plot Examining Publication Bias for Pooled Prevalence of Microalbuminuria Outcome

eFigure 20. Funnel Plot Examining Publication Bias for Pooled Prevalence of Persistent Microalbuminuria Outcome

eFigure 21. Distribution of Risk of Bias Sources in the Included Studies

eAppendix. List of Studies Excluded at the Full-Text Screening Stage

eReferences.

This supplemental material has been provided by the authors to give readers additional information about their work.

eTable 1. Search Strategy, MEDLINE

1	exp Diabetes Mellitus, Type 2/
2	NIDDM.ti,ab,kf.
3	MODY.ti,ab,kf.
4	t2d*.ti,ab,kf.
5	((typ* two or typ?two or typ* 2 or typ* II or typ?2 or typ?II or typ* ii or typ?ii) adj4 diabet*).ti,ab,kf.
6	((non insulin or noninsulin or late or adult* or matur* or slow or stabl*) adj4 diabet*).ti,ab,kf.
7	((ketoresist* or keto* resist* or keto* prone) adj4 diabet*).ti,ab,kf.
8	or/1-7
9	exp Child/
10	child*.ti,ab,kf.
11	adolescen*.ti,ab,kf.
12	exp Adolescent/
13	youth*.ti,ab,kf.
14	teenage*.ti,ab,kf.
15	preadolescen*.ti,ab,kf.
16	Pediatrics/
17	p?ediatric*.ti,ab,kf.
18	pe?diatric*.ti,ab,kf.
19	or/9-18
20	8 and 19
21	exp Proteinuria/
22	proteinuria*.ti,ab,kf.
23	albuminuria*.ti,ab,kf.
24	hemoglobinuria*.ti,ab,kf.
25	microalbuminuria*.ti,ab,kf.
26	micro albuminuria*.ti,ab,kf.
27	macroalbuminuria*.ti,ab,kf.
28	macro albuminuria*.ti,ab,kf.
29	Creatinine/ and (ur.fs. or Urine/)
30	creatininuria*.ti,ab,kf.
31	(creatinine and urine*).ti,ab,kf.
32	exp Diabetic Nephropathies/
33	(diabet* adj3 (nephropath* or glomerulopath* or glomerulosclerosis or nephrosclerosis)).ti,ab,kf.
34	or/21-33
35	hypertension/ or diabetic hypertension/ or malignant hypertension/ or exp renovascular hypertension/
36	hypertens*.ti,ab,kw.
37	elevated blood pressure/

© 2021 Cioana M et al. *JAMA Network Open.*

38	((elevat* or high* or rais*) adj3 (diastolic or systolic or arterial or blood) adj1 pressure*).ti,ab,kw.
39	or/35-38
40	34 or 39
41	Prevalence/
42	prevalence.ti,ab,kf.
43	prevalence studies/
44	Incidence/
45	incidence studies/
46	incidence.ti,ab,kf.
47	Epidemiology/
48	epidemiolog*.ti,ab,kf.
49	ep.fs.
50	epidemiologic methods/ or epidemiological monitoring/ or sentinel surveillance/
51	exp epidemiologic studies/
52	case-control.ti,ab,kf.
53	cohort.ti,ab,kf.
54	prospective.ti,ab,kf.
55	longitudinal.ti,ab,kf.
56	retrospective.ti,ab,kf.
57	cross sectional.ti,ab,kf.
58	correlational.ti,ab,kf.
59	or/41-58
60	20 and 40 and 59
61	60 not (animals/ not (humans/ and animals/))
62	remove duplicates from 61

© 2021 Cioana M et al. *JAMA Network Open.*

eTable 2. Search Strategy, Embase

1	non insulin dependent diabetes mellitus/
2	NIDDM.ti,ab,kw.
3	MODY.ti,ab,kw.
4	t2d*.ti,ab,kw.
5	((typ* two or typ?two or typ* 2 or typ* II or typ?2 or typ?II or typ* ii or typ?ii) adj4 diabet*).ti,ab,kw.
6	((non insulin or noninsulin or late or adult* or matur* or slow or stabl*) adj4 diabet*).ti,ab,kw.
7	((ketoresist* or keto* resist*) adj6 diabet*).ti,ab,kw.
8	or/1-7
9	exp child/
10	child*.ti,ab,kw.
11	adolescent/
12	adolescen*.ti,ab,kw.
13	youth*.ti,ab,kw.
14	teenage*.ti,ab,kw.
15	preadolescen*.ti,ab,kw.
16	pediatrics/
17	p?ediatric*.ti,ab,kw.
18	pe?diatric*.ti,ab,kw.
19	or/9-18
20	8 and 19
21	exp proteinuria/
22	proteinuria*.ti,ab,kw.
23	albuminuria*.ti,ab,kw.
24	hemoglobinuria*.ti,ab,kw.
25	microalbuminuria*.ti,ab,kw.
26	micro albuminuria*.ti,ab,kw.
27	macroalbuminuria*.ti,ab,kw.
28	macro albuminuria*.ti,ab,kw.
29	creatinine urine level/
30	creatininuria*.ti,ab,kw.
31	(creatinine and urine*).ti,ab,kw.
32	diabetic nephropathy/
33	(diabet* adj3 (nephropath* or glomerulopath* or glomerulosclerosis or nephrosclerosis)).ti,ab,kw.
34	or/21-33
35	hypertension/ or diabetic hypertension/ or malignant hypertension/ or exp renovascular hypertension/
36	hypertens*.ti,ab,kw.
37	elevated blood pressure/

© 2021 Cioana M et al. *JAMA Network Open.*

38	((elevat* or high* or rais*) adj3 (diastolic or systolic or arterial or blood) adj1 pressure*).ti,ab,kw.
39	or/35-38
40	34 or 39
41	20 and 40
42	prevalence/
43	prevalence.ti,ab,kw.
44	incidence/
45	incidence.ti,ab,kw.
46	epidemiology/
47	epidemiolog*.ti,ab,kw.
48	ep.fs.
49	epidemiological monitoring/
50	sentinel surveillance/
51	case-control.ti,ab,kw.
52	cohort.ti,ab,kw.
53	prospective.ti,ab,kw.
54	longitudinal.ti,ab,kw.
55	retrospective.ti,ab,kw.
56	cross sectional.ti,ab,kw.
57	correlational.ti,ab,kw.
58	or/42-57
59	41 and 58
60	59 not (exp animals/ not (humans/ and animals/))
61	remove duplicates from 60

© 2021 Cioana M et al. *JAMA Network Open.*

eTable 3. Search Strategy, CINAHL

#	Query	Limiters/Expanders	Last Run Via
S1	(MH "Child+")	Search modes - Boolean/Phrase	Interface - EBSCOhost Research Databases Search Screen - Advanced Search Database - CINAHL
S2	"child*"	Search modes - Boolean/Phrase	Interface - EBSCOhost Research Databases Search Screen - Advanced Search Database - CINAHL
S3	(MH "Adolescence+")	Search modes - Boolean/Phrase	Interface - EBSCOhost Research Databases Search Screen - Advanced Search Database - CINAHL
S4	"youth*"	Search modes - Boolean/Phrase	Interface - EBSCOhost Research Databases Search Screen - Advanced Search Database - CINAHL
S5	"teenage*"	Search modes - Boolean/Phrase	Interface - EBSCOhost Research Databases Search Screen - Advanced Search Database - CINAHL
S6	(MH "Pediatrics")	Search modes - Boolean/Phrase	Interface - EBSCOhost Research Databases Search Screen - Advanced Search Database - CINAHL
S7	"p?ediatric*"	Search modes - Boolean/Phrase	Interface - EBSCOhost Research Databases Search Screen - Advanced Search Database - CINAHL
S8	"p#ediatric*"	Search modes - Boolean/Phrase	Interface - EBSCOhost Research Databases Search Screen - Advanced Search Database - CINAHL
S9	"pe#diatric*"	Search modes - Boolean/Phrase	Interface - EBSCOhost Research Databases Search Screen - Advanced Search Database - CINAHL
S10	"pediatric*"	Search modes - Boolean/Phrase	Interface - EBSCOhost Research Databases Search Screen - Advanced Search Database - CINAHL
S11	"preadolescen*"	Search modes - Boolean/Phrase	Interface - EBSCOhost Research Databases Search Screen - Advanced Search Database - CINAHL
S12	S1 OR S2 OR S3 OR S4 OR S5 OR S6 OR S7 OR S8 OR S9 OR S10 OR S11	Search modes - Boolean/Phrase	Interface - EBSCOhost Research Databases Search Screen - Advanced Search Database - CINAHL
S13	(MH "Diabetes Mellitus, Type 2")	Search modes - Boolean/Phrase	Interface - EBSCOhost Research Databases Search Screen - Advanced Search Database - CINAHL

© 2021 Cioana M et al. *JAMA Network Open.*

S14	"NIDDM"	Search modes - Boolean/Phrase	Interface - EBSCOhost Research Databases Search Screen - Advanced Search Database - CINAHL
S15	"MODY"	Search modes - Boolean/Phrase	Interface - EBSCOhost Research Databases Search Screen - Advanced Search Database - CINAHL
S16	"T2D*"	Search modes - Boolean/Phrase	Interface - EBSCOhost Research Databases Search Screen - Advanced Search Database - CINAHL
S17	(typ* two or typ?two or typ* 2 or typ* II or typ?2 or typ? II) N4 diabet*	Search modes - Boolean/Phrase	Interface - EBSCOhost Research Databases Search Screen - Advanced Search Database - CINAHL
S18	(non insulin or noninsulin or late or adult* or matur* or slow or stabl*) N4 diabet*	Search modes - Boolean/Phrase	Interface - EBSCOhost Research Databases Search Screen - Advanced Search Database - CINAHL
S19	(ketoresist* or keto* resist* or keto* prone) adj4 diabet*	Search modes - Boolean/Phrase	Interface - EBSCOhost Research Databases Search Screen - Advanced Search Database - CINAHL
S20	S13 OR S14 OR S15 OR S16 OR S17 OR S18 OR S19	Search modes - Boolean/Phrase	Interface - EBSCOhost Research Databases Search Screen - Advanced Search Database - CINAHL
S21	S12 AND S20	Search modes - Boolean/Phrase	Interface - EBSCOhost Research Databases Search Screen - Advanced Search Database - CINAHL
S22	(MH "Hypertension") OR (MH "Hypertension, Renal+") OR (MH "Hypertension, Malignant")	Search modes - Boolean/Phrase	Interface - EBSCOhost Research Databases Search Screen - Advanced Search Database - CINAHL
S23	"hypertens*"	Search modes - Boolean/Phrase	Interface - EBSCOhost Research Databases Search Screen - Advanced Search Database - CINAHL
S24	((elevat* or high* or rais*) N3 (diastolic or systolic or arterial or blood) N1 pressure*)	Search modes - Boolean/Phrase	Interface - EBSCOhost Research Databases Search Screen - Advanced Search Database - CINAHL
S25	S22 OR S23 OR S24	Search modes - Boolean/Phrase	Interface - EBSCOhost Research Databases Search Screen - Advanced Search Database - CINAHL
S26	(MH "Proteinuria+")	Search modes - Boolean/Phrase	Interface - EBSCOhost Research Databases Search Screen - Advanced Search Database - CINAHL
S27	"proteinuria*"	Search modes - Boolean/Phrase	Interface - EBSCOhost Research Databases Search Screen -

© 2021 Cioana M et al. *JAMA Network Open.*

			Advanced Search Database - CINAHL
S28	"albuminuria*"	Search modes - Boolean/Phrase	Interface - EBSCOhost Research Databases Search Screen - Advanced Search Database - CINAHL
S29	"hemoglobinuria*"	Search modes - Boolean/Phrase	Interface - EBSCOhost Research Databases Search Screen - Advanced Search Database - CINAHL
S30	"microalbuminuria*"	Search modes - Boolean/Phrase	Interface - EBSCOhost Research Databases Search Screen - Advanced Search Database - CINAHL
S31	"micro albuminuria*"	Search modes - Boolean/Phrase	Interface - EBSCOhost Research Databases Search Screen - Advanced Search Database - CINAHL
S32	"macroalbuminuria*"	Search modes - Boolean/Phrase	Interface - EBSCOhost Research Databases Search Screen - Advanced Search Database - CINAHL
S33	"macro albuminuria*"	Search modes - Boolean/Phrase	Interface - EBSCOhost Research Databases Search Screen - Advanced Search Database - CINAHL
S34	(MH "Creatinine/UR")	Search modes - Boolean/Phrase	Interface - EBSCOhost Research Databases Search Screen - Advanced Search Database - CINAHL
S35	MH creatinine AND MH urine	Search modes - Boolean/Phrase	Interface - EBSCOhost Research Databases Search Screen - Advanced Search Database - CINAHL
S36	S26 OR S27 OR S28 OR S29 OR S30 OR S31 OR S32 OR S33 OR S34 OR S35	Search modes - Boolean/Phrase	Interface - EBSCOhost Research Databases Search Screen - Advanced Search Database - CINAHL
S37	S25 OR S36	Search modes - Boolean/Phrase	Interface - EBSCOhost Research Databases Search Screen - Advanced Search Database - CINAHL
S38	"prevalence"	Search modes - Boolean/Phrase	Interface - EBSCOhost Research Databases Search Screen - Advanced Search Database - CINAHL
S39	(MH "Cross Sectional Studies")	Search modes - Boolean/Phrase	Interface - EBSCOhost Research Databases Search Screen - Advanced Search Database - CINAHL
S40	"cross section*"	Search modes - Boolean/Phrase	Interface - EBSCOhost Research Databases Search Screen - Advanced Search Database - CINAHL

© 2021 Cioana M et al. *JAMA Network Open.*

S41	(MH "Incidence")	Search modes - Boolean/Phrase	Interface - EBSCOhost Research Databases Search Screen - Advanced Search Database - CINAHL
S42	"incidence"	Search modes - Boolean/Phrase	Interface - EBSCOhost Research Databases Search Screen - Advanced Search Database - CINAHL
S43	(MH "Epidemiology")	Search modes - Boolean/Phrase	Interface - EBSCOhost Research Databases Search Screen - Advanced Search Database - CINAHL
S44	"epidemiolog*"	Search modes - Boolean/Phrase	Interface - EBSCOhost Research Databases Search Screen - Advanced Search Database - CINAHL
S45	(MH "Epidemiological Research")	Search modes - Boolean/Phrase	Interface - EBSCOhost Research Databases Search Screen - Advanced Search Database - CINAHL
S46	(MH "Prospective Studies") OR (MH "Cross Sectional Studies") OR (MH "Case Control Studies") OR (MH "Correlational Studies")	Search modes - Boolean/Phrase	Interface - EBSCOhost Research Databases Search Screen - Advanced Search Database - CINAHL
S47	"case control" or "cohort" or "prospective" or "retrospective" or "longitudinal" or "correlational"	Search modes - Boolean/Phrase	Interface - EBSCOhost Research Databases Search Screen - Advanced Search Database - CINAHL
S48	S38 OR S39 OR S40 OR S41 OR S42 OR S43 OR S44 OR S45 OR S46 OR S47	Search modes - Boolean/Phrase	Interface - EBSCOhost Research Databases Search Screen - Advanced Search Database - CINAHL
S49	(S21 AND S37 AND S48) NOT (MH "Animals")	Search modes - Boolean/Phrase	Interface - EBSCOhost Research Databases Search Screen - Advanced Search Database - CINAHL

© 2021 Cioana M et al. *JAMA Network Open.*

eTable 4. Search Strategy, Cochrane Library: Cochrane Central Register of Controlled Trials and Cochrane Database of Systematic Reviews

child* OR youth* OR teenage* OR adolescen* OR pediatric* OR preadolescen* OR p?ediatric* OR pe?diatric* in Title Abstract Keyword	AND
NIDDM OR MODY OR t2d OR typ* two NEAR/4 diabet* OR typ?two NEAR/4 diabet* OR typ* 2 NEAR/4 diabet* OR typ* II NEAR/4 diabet* OR typ?2 NEAR/4 diabet* OR typ?II NEAR/4 diabet* OR typ* ii NEAR/4 diabet* OR typ?ii NEAR/4 diabet* OR non insulin NEAR/4 diabet* OR noninsulin NEAR/4 diabet* OR late or adult* NEAR/4 diabet* OR matur* NEAR/4 diabet* OR slow NEAR/4 diabet* OR stabl* NEAR/4 diabet* OR ketoresist* NEAR/4 diabet* OR keto* resist* NEAR/4 diabet* OR keto* prone NEAR/4 diabet* in Title Abstract Keyword	AND
(proteinuria* OR albuminuria* OR hemoglobinuria* OR microalbuminuria* OR "micro albuminuria*" OR macroalbuminuria* OR "macro albuminuria*" OR creatininuria* OR (creatinine and urine*) OR diabet* NEAR nephropath* OR diabet* NEAR glomerulopath* OR diabet* NEAR glomerulosclerosis OR diabet* NEAR nephrosclerosis) OR (hypertens* OR elevat* NEAR/3 diastolic NEAR/1 pressure* OR elevat* NEAR/3 systolic NEAR/1 pressure* OR elevat* NEAR/3 arterial NEAR/1 pressure* OR elevat* NEAR/3 blood NEAR/1 pressure* OR high* NEAR/3 diastolic NEAR/1 pressure* OR high* NEAR/3 systolic NEAR/1 pressure* OR high* NEAR/3 arterial NEAR/1 pressure* OR high* NEAR/3 blood NEAR/1 pressure* OR rais* NEAR/3 diastolic NEAR/1 pressure* OR rais* NEAR/3 systolic NEAR/1 pressure* OR rais* NEAR/3 arterial NEAR/1 pressure* OR rais* NEAR/3 blood NEAR/1 pressure*) in Title Abstract Keyword	
(Word variations have been searched)	

© 2021 Cioana M et al. *JAMA Network Open.*

eTable 5. Search Strategy, Web of Science: Conference Proceedings Citation Index-Science

#1	TI=(child* OR youth* OR teenage* OR adolescen* OR pediatric* OR preadolescen* OR p?ediatric* OR pe?diatric*)
#2	TS=(child* OR youth* OR teenage* OR adolescen* OR pediatric* OR preadolescen* OR p?ediatric* OR pe?diatric*)
#3	#1 OR #2
#4	TI=(NIDDM OR MODY OR t2d OR typ* two NEAR/4 diabet* OR typ?two NEAR/4 diabet* OR typ* 2 NEAR/4 diabet* OR typ* II NEAR/4 diabet* OR typ?2 NEAR/4 diabet* OR typ?II NEAR/4 diabet* OR typ* ii NEAR/4 diabet* OR typ?ii NEAR/4 diabet* OR non insulin NEAR/4 diabet* OR noninsulin NEAR/4 diabet* OR late or adult* NEAR/4 diabet* OR matur* NEAR/4 diabet* OR slow NEAR/4 diabet* OR stabl* NEAR/4 diabet* OR ketoresist* NEAR/4 diabet* OR keto* resist* NEAR/4 diabet* OR keto* prone NEAR/4 diabet*)
#5	TS=(NIDDM OR MODY OR t2d OR typ* two NEAR/4 diabet* OR typ?two NEAR/4 diabet* OR typ* 2 NEAR/4 diabet* OR typ* II NEAR/4 diabet* OR typ?2 NEAR/4 diabet* OR typ?II NEAR/4 diabet* OR typ* ii NEAR/4 diabet* OR typ?ii NEAR/4 diabet* OR non insulin NEAR/4 diabet* OR noninsulin NEAR/4 diabet* OR late or adult* NEAR/4 diabet* OR matur* NEAR/4 diabet* OR slow NEAR/4 diabet* OR stabl* NEAR/4 diabet* OR ketoresist* NEAR/4 diabet* OR keto* resist* NEAR/4 diabet* OR keto* prone NEAR/4 diabet*)
#6	#4 OR #5
#7	#3 AND #6
#8	TI=(hypertens* OR elevat* NEAR/4 diastolic pressure* OR elevat* NEAR/4 systolic pressure* OR elevat* NEAR/4 arterial pressure* OR elevat* NEAR/4 blood pressure* OR high* NEAR/4 diastolic pressure* OR high* NEAR/4 systolic pressure* OR high* NEAR/4 arterial pressure* OR high* NEAR/4 blood pressure* OR rais* NEAR/4 diastolic pressure* OR rais* NEAR/4 systolic pressure* OR rais* NEAR/4 arterial pressure* OR rais* NEAR/4 blood pressure*)
#9	TS=(hypertens* OR elevat* NEAR/4 diastolic pressure* OR elevat* NEAR/4 systolic pressure* OR elevat* NEAR/4 arterial pressure* OR elevat* NEAR/4 blood pressure* OR high* NEAR/4 diastolic pressure* OR high* NEAR/4 systolic pressure* OR high* NEAR/4 arterial pressure* OR high* NEAR/4 blood pressure* OR rais* NEAR/4 diastolic pressure* OR rais* NEAR/4 systolic pressure* OR rais* NEAR/4 arterial pressure* OR rais* NEAR/4 blood pressure*)
#10	TI=(proteinuria* OR albuminuria* OR hemoglobinuria* OR microalbuminuria* OR micro albuminuria* OR macroalbuminuria* OR macro albuminuria* OR creatininuria* OR (creatinine and urine*) OR diabet* NEAR/4 nephropath* OR diabet* NEAR/4 glomerulopath* OR diabet* NEAR/4 glomerulosclerosis OR diabet* NEAR/4 nephrosclerosis)
#11	TS=(proteinuria* OR albuminuria* OR hemoglobinuria* OR microalbuminuria* OR micro albuminuria* OR macroalbuminuria* OR macro albuminuria* OR creatininuria* OR (creatinine and urine*) OR diabet* NEAR/4 nephropath* OR diabet* NEAR/4 glomerulopath* OR diabet* NEAR/4 glomerulosclerosis OR diabet* NEAR/4 nephrosclerosis)
#12	#8 OR #9 OR #10 OR #11
#13	#7 AND #12

© 2021 Cioana M et al. *JAMA Network Open.*

eTable 6. Results of Sensitivity Analysis for Prevalence of Hypertension in Pediatric Type 2 Diabetes Meta-analysis

Studies removed	Pooled prevalence estimate (%, 95% CI)	I^2, χ^2 p-value
Conference abstracts[2–4]	26% (95% CI 20-33)	95%, <0.001
Sample size <50[4–17]	27% (95% CI 20-35)	96%, <0.001
Patients over 18 years old[5–7,12,13,15,17–20]	26% (95% CI 19-34)	96%, <0.001
Different definition[2–4,6,7,9,12,13,16–18,20–24]	34% (95% CI 24-45)	97%, <0.001

© 2021 Cioana M et al. *JAMA Network Open.*

eTable 7. Results of Sensitivity Analysis for Prevalence of Albuminuria in Pediatric Type 2 Diabetes Meta-analysis

Studies removed	Pooled prevalence estimate (%, 95%CI)	I^2, χ^2 p-value
Conference abstracts[4]	23% (95%CI 18-28)	83%, <0.001
Sample size <50[4,8,15,16,25,26]	20% (95%CI 15-25)	87%, <0.001
Patients over 18 years old[15,18,25–29]	17% (95%CI 9-27)	86%, <0.001
Different definition[4,15,16,23,26,27,30,31]	25% (95%CI 20-31)	57%, 0.04

© 2021 Cioana M et al. *JAMA Network Open.*

eTable 8. Results of Sensitivity Analysis for Prevalence of Persistent Albuminuria in Pediatric Type 2 Diabetes Meta-analysis

Studies removed	Pooled prevalence estimate (%, 95%CI)	I^2, χ^2 p-value
Sample size <50[12,14,17,32–34]	28% (95%CI 13-46)	99%, <0.001
Patients over 18 years old[12,17,19,20,33–35]	24% (95%CI 15-35)	92%, <0.001
Different definition[12,14,19,20,31,32,34,35]	17% (95%CI 7-29)	92%, <0.001

© 2021 Cioana M et al. *JAMA Network Open.*

eTable 9. Results of Sensitivity Analysis for Prevalence of Microalbuminuria in Pediatric Type 2 Diabetes Meta-analysis

Studies removed	Pooled prevalence estimate (%, 95%CI)	I^2, χ^2 p-value
Conference abstracts[3,36–38]	21% (95%CI 14-29)	92%, <0.001
Sample size <50[5,8,15,25,26,36,38,39]	14% (95%CI 9-20)	92%, <0.001
Patients over 18 years old[5,15,18,25–27,40]	24% (95%CI 14-36)	86%, <0.001
Different definition[3,5,15,26,27,36,38,40]	19% (95%CI 11-27)	83%, <0.001

© 2021 Cioana M et al. *JAMA Network Open.*

eTable 10. Results of Sensitivity Analysis for Prevalence of Persistent Microalbuminuria in Pediatric Type 2 Diabetes Meta-analysis

Studies removed	Pooled prevalence estimate (%, 95%CI)	I^2, χ^2 p-value
Sample size <50[12,14,19,33,34,39]	35% (95%CI 15-60)	98%, <0.001
Patients over 18 years old[12,19,20,33,34]	23% (95%CI 14-34)	88%, <0.001
Different definition[12,14,20,32,34,41]	24% (95%CI 11-39)	84%, <0.001

© 2021 Cioana M et al. *JAMA Network Open.*

eTable 11. Risk of Bias and OCEBM Level of Evidence of Included Studies

	External Validity Items				Internal Validity Items						Overall Score	Overall Risk of Bias	OCEMB Level of Evidence Rating
Author, year (country)	1	2	3	4	5	6	7	8	9	10			
Amed, 2012 (Canada)[23]	1	0	0	1	1	1	1	0	1	1	7	moderate	3
Amutha, 2012 (India)[18]	0	1	1	1	1	1	1	1	1	1	9	low	1
Aulich, 2019 (Australia)[14]	0	1	0	0	1	1	1	1	1	1	7	moderate	3
Balasanthiran, 2012 (UK)[6]	0	0	1	1	1	1	1	1	1	1	8	moderate	2
Bell, 2009 (USA)[42]	0	1	1	1	1	1	1	1	1	1	9	low	1
Calagua Quipse, 2015 (Peru)[36]	0	0	0	1	1	0	1	1	1	1	6	moderate	3
Candler, 2018 (UK and Republic of Ireland)[30]	1	1	1	1	1	1	1	0	1	1	9	low	1
Copeland, 2011 (USA)[43]	1	1	0	1	1	1	1	1	1	1	9	low	3
Cruz, 2004 (Mexico)[44]	0	0	1	1	1	1	1	1	1	1	8	moderate	2
Curran, 2020 (Australia)[16]	1	1	1	1	1	0	1	1	1	1	9	low	2
Dabelea, 2009 (USA)[45]	0	1	1	1	1	1	1	1	1	1	9	low	1
Dart, 2012 (Canada)[41]	0	1	1	1	1	1	1	1	1	1	9	low	1
Dart, 2014 (Canada)[22]	0	1	1	1	1	1	1	1	1	1	9	low	1
Dart, 2019 (Canada)[31]	0	0	1	1	1	1	1	1	1	1	8	moderate	1
Drutel, 2014 (USA)[2]	0	0	0	1	1	1	1	1	1	1	7	moderate	3
Eppens, 2006 (Australia)[32]	0	1	1	0	1	1	1	1	1	1	8	moderate	2
Eppens, 2006 (Western Pacific)[46]	1	0	1	1	1	1	1	1	1	1	9	low	1
Ettinger, 2005 (USA)[8]	0	0	0	1	1	1	1	1	1	1	7	moderate	3
Farah, 2006 (USA)[33]	0	0	0	1	1	1	1	1	1	0	6	moderate	3
Haynes, 2014 (Australia)[3]	0	1	1	0	1	1	1	1	1	1	8	moderate	1
Holman, 2015 (UK)[47]	1	1	1	0	1	1	1	0	1	1	8	moderate	2
Hotu, 2004 (New Zealand)[25]	0	1	1	1	1	1	1	1	1	1	9	low	2
Jefferies, 2012 (New Zealand)[19]	0	1	1	1	1	1	1	1	1	1	9	low	1
Kim, 2010 (USA)[48]	0	1	1	0	1	1	1	1	1	1	8	moderate	1

© 2021 Cioana M et al. *JAMA Network Open.*

Khalil, 2019 (Egypt)[17]	0	1	1	0	1	1	1	1	1	1	8	moderate	2
Klingensmith, 2016 (USA)[49]	1	1	1	1	1	1	1	0	1	1	9	low	1
Lawrence, 2009 (USA)[50]	0	1	1	1	1	1	1	1	1	1	9	low	1
Le, 2013 (USA)[51]	0	0	1	0	1	1	1	1	1	1	7	moderate	1
Liu, 2009 (API) (USA)[52]	0	1	1	0	1	1	1	1	1	1	8	moderate	2
Maahs, 2007 (USA)[28]	1	1	1	1	1	1	1	1	1	1	10	low	1
Mayer-Davis, 2009 (USA)[53]	0	1	1	1	1	1	1	1	1	1	9	low	1
Nambam, 2017 (USA)[40]	1	1	1	1	1	1	1	0	1	1	9	low	1
Newton, 2015 (New Zealand)[38]	0	1	1	0	1	1	1	1	1	1	8	moderate	2
Osman, 2013 (Sudan)[5]	0	1	1	1	1	1	1	1	1	1	9	low	2
Pelham, 2019 (USA)[54]	0	0	1	1	1	1	1	1	1	1	8	moderate	1
Pérez-Perdomo, 2005 (Puerto Rico)[7]	1	0	1	0	1	1	1	0	1	1	7	moderate	2
Pinhas-Hamiel, 1996 (USA)[24]	0	1	1	1	1	1	1	1	1	1	9	low	1
Reinehr, 2005 (Germany)[11]	0	1	1	0	1	1	1	1	1	1	8	moderate	2
Rodriguez, 2010 (USA)[55]	1	1	1	1	1	1	1	1	1	1	10	low	1
Ruhayel, 2010 (Australia)[26]	0	1	1	0	1	1	1	1	1	1	8	moderate	2
Schmidt, 2012 (Germany and Austria)[27]	1	1	1	0	1	1	1	0	1	1	8	moderate	1
Scott, 1997 (USA)[9]	0	0	0	1	1	1	1	1	1	1	7	moderate	3
Scott, 2004 (New Zealand)[12]	0	1	1	1	1	1	1	0	1	1	8	moderate	2
Scott, 2006 (New Zealand)[20]	1	1	1	0	1	1	1	0	1	1	8	moderate	1
Sellers, 2007 (Canada)[56]	0	1	1	1	1	1	1	1	1	1	9	low	1
Sellers, 2009 (Canada)[29]	0	1	1	1	1	1	1	1	1	1	9	low	1
Sellers, 2016 (Canada)[35]	1	0	1	1	1	1	1	0	1	1	8	moderate	1
Shalitin, 2014 (Israel)[57]	0	0	0	1	1	1	1	1	1	0	6	moderate	3
Shield, 2009 (UK and Republic of Ireland)[58]	1	1	1	1	1	1	1	0	1	1	9	low	1
Son, 2015 (Korea)[39]	0	0	0	1	1	1	1	1	1	1	7	moderate	3
Unnikrishnan, 2008 (India)[13]	1	1	1	1	1	1	1	0	1	1	9	low	2

© 2021 Cioana M et al. *JAMA Network Open.*

Upchurch, 2003 (USA)[59]	0	0	0	0	1	1	1	1	1	1	6	moderate	3
Urakami, 2009 (Japan)	0	0	0	1	1	1	1	1	1	1	7	moderate	3
Wei, 2003 (Taiwan)[60]	1	1	1	1	1	1	1	1	1	1	10	low	1
Yafi, 2019 (USA)[4]	0	0	0	1	1	0	1	1	1	1	6	moderate	3
Yeow, 2019 (Malaysia)[15]	0	1	1	0	1	1	1	1	1	1	8	moderate	2
Yoo, 2004 (Korea)[34]	0	0	0	1	1	1	1	1	1	1	7	moderate	3
Zabeen, 2016 (abstract) (Bangladesh)[37]	0	1	1	1	1	1	1	1	1	1	9	low	1
Zabeen, 2016 (Bangladesh)[61]	0	1	1	1	1	1	1	1	1	1	9	low	1
Zdravkovic, 2004 (Canada)[10]	0	1	1	1	1	1	1	1	1	1	9	low	2

Footnote: 0: no, 1: yes, overall risk of bias: low (score >8), moderate (score 6-8), or high (score ≤5). Items scored: 1) Was the study's target population a close representation of the national population in relation to relevant variables, e.g., age, sex?; 2) Was the sampling frame a true or close representation of the target population?; 3) Was some form of random selection used to select the sample, OR, was a census undertaken?; 4) Was the likelihood of non-response bias minimal?; 5) Were data collected directly from the subjects (as opposed to a proxy)?; 6) Was an acceptable case definition used in the study?; 7) Had the study instrument that measured the parameter of interest (e.g., prevalence of comorbidity) been tested for reliability and validity (if necessary)?; 8) Was the same mode of data collection used for all subjects?; 9) Was the length of the shortest prevalence period for the parameter of interest appropriate?; 10) Were the numerator(s) and denominator(s) for the parameter of interest appropriate?

© 2021 Cioana M et al. *JAMA Network Open.*

eFigure 1. Study Flow Diagram

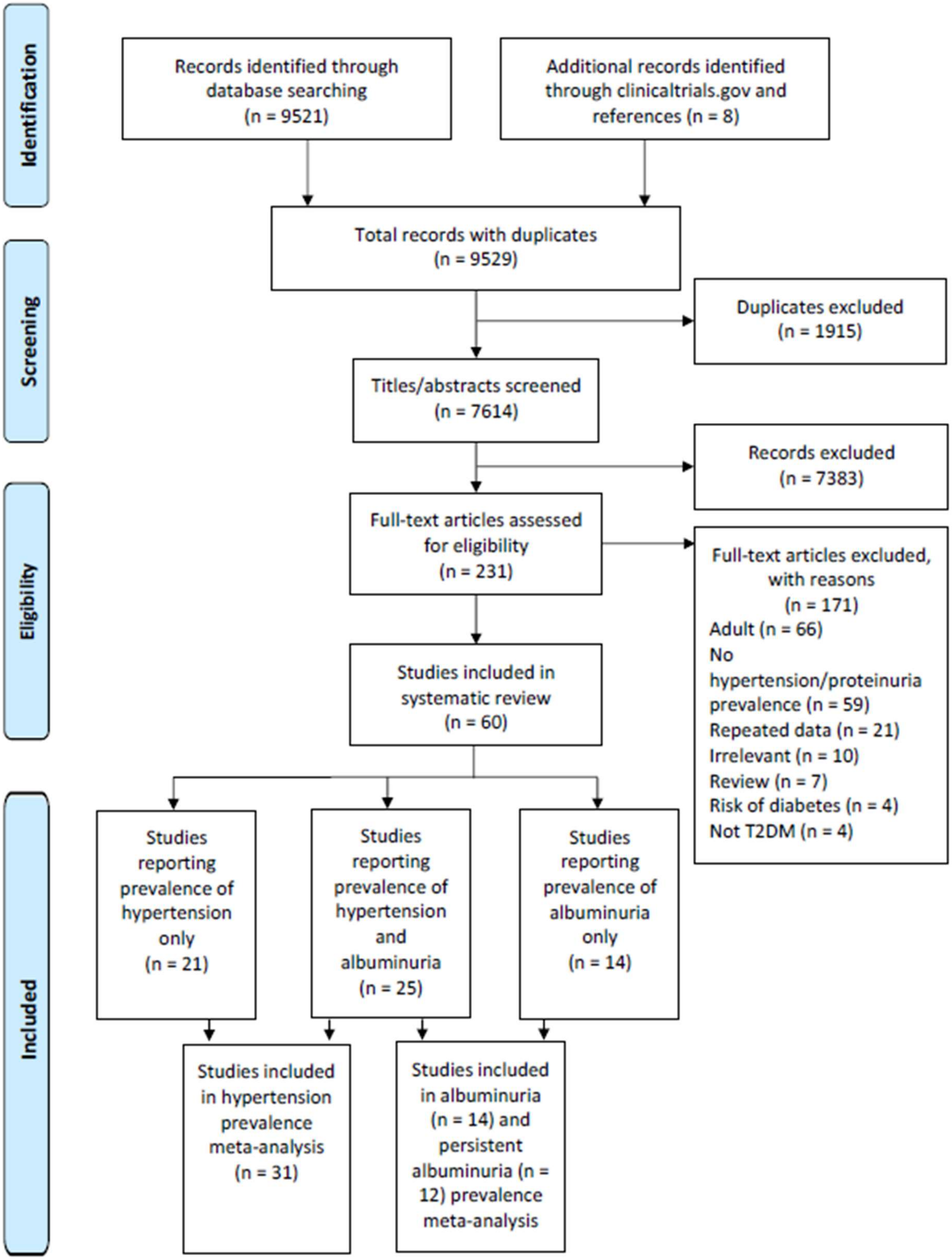

© 2021 Cioana M et al. *JAMA Network Open.*

eFigure 2. Forest Plot Showing Pooled Prevalence of Systolic Hypertension in Pediatric Type 2 Diabetes

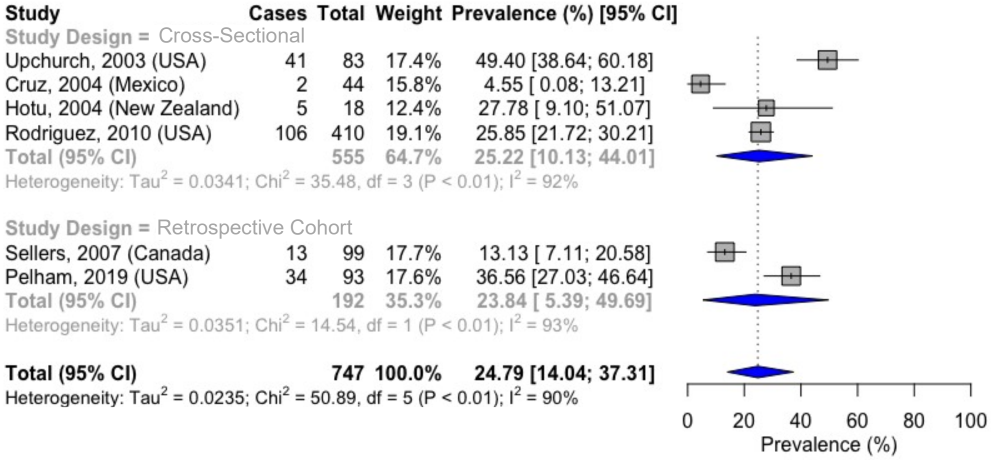

© 2021 Cioana M et al. *JAMA Network Open.*

eFigure 3. Forest Plot Showing Pooled Prevalence of Diastolic Hypertension in Pediatric Type 2 Diabetes

Study	Cases	Total	Weight	Prevalence (%) [95% CI]
**Study Design =** Cross-Sectional				
Upchurch, 2003 (USA)	9	83	18.1%	10.84 [4.93; 18.56]
Cruz, 2004 (Mexico)	5	44	14.5%	11.36 [3.37; 22.70]
Rodriguez, 2010 (USA)	75	410	23.3%	18.29 [14.69; 22.19]
Shalitin, 2014 (Israel)	4	11	6.6%	36.36 [10.00; 67.31]
**Total (95% CI)**		**548**	**62.4%**	**15.06 [9.27; 21.83]**
Heterogeneity: $Tau^2 = 0.0034$; $Chi^2 = 6.12$, df = 3 (P = 0.11); $I^2 = 51\%$				
**Study Design =** Retrospective Cohort				
Sellers, 2007 (Canada)	6	99	18.9%	6.06 [2.06; 11.76]
Pelham, 2019 (USA)	7	93	18.6%	7.53 [2.90; 13.90]
**Total (95% CI)**		**192**	**37.6%**	**6.75 [3.52; 10.85]**
Heterogeneity: $Tau^2 = 0$; $Chi^2 = 0.16$, df = 1 (P = 0.69); $I^2 = 0\%$				
**Total (95% CI)**		**740**	**100.0%**	**11.65 [6.41; 18.04]**
Heterogeneity: $Tau^2 = 0.0077$; $Chi^2 = 19.80$, df = 5 (P < 0.01); $I^2 = 75\%$				

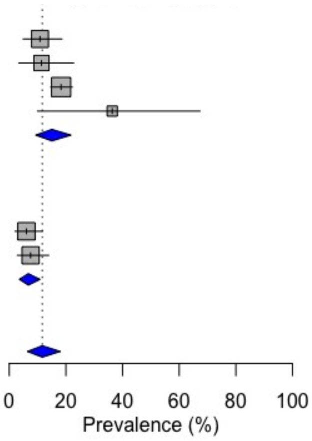

© 2021 Cioana M et al. *JAMA Network Open.*

eFigure 4. Forest Plot Showing Pooled Prevalence of Hypertension in Pediatric Type 2 Diabetes by Sex

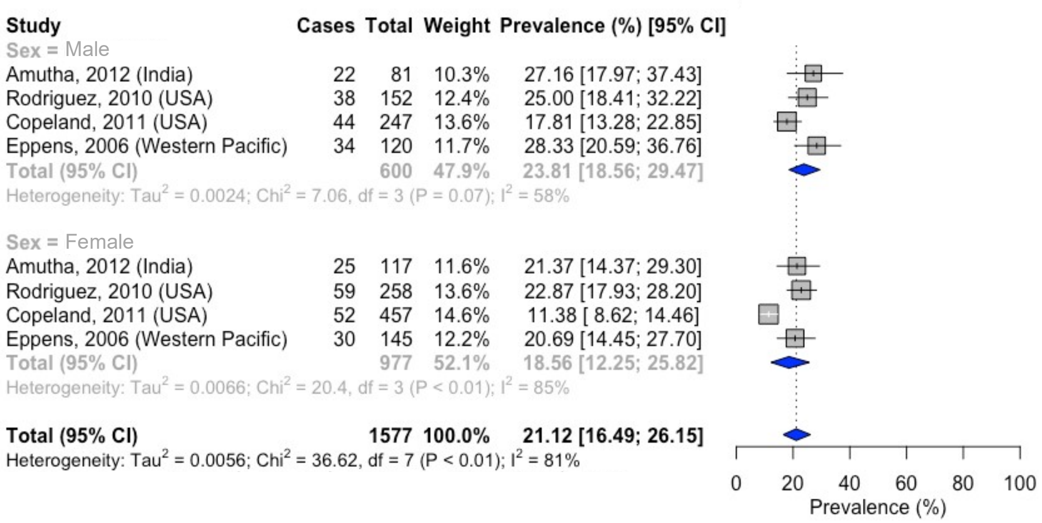

Study	Cases	Total	Weight	Prevalence (%) [95% CI]
**Sex = Male**				
Amutha, 2012 (India)	22	81	10.3%	27.16 [17.97; 37.43]
Rodriguez, 2010 (USA)	38	152	12.4%	25.00 [18.41; 32.22]
Copeland, 2011 (USA)	44	247	13.6%	17.81 [13.28; 22.85]
Eppens, 2006 (Western Pacific)	34	120	11.7%	28.33 [20.59; 36.76]
**Total (95% CI)**		**600**	**47.9%**	**23.81 [18.56; 29.47]**
Heterogeneity: $Tau^2 = 0.0024$; $Chi^2 = 7.06$, df = 3 (P = 0.07); $I^2 = 58\%$				
**Sex = Female**				
Amutha, 2012 (India)	25	117	11.6%	21.37 [14.37; 29.30]
Rodriguez, 2010 (USA)	59	258	13.6%	22.87 [17.93; 28.20]
Copeland, 2011 (USA)	52	457	14.6%	11.38 [8.62; 14.46]
Eppens, 2006 (Western Pacific)	30	145	12.2%	20.69 [14.45; 27.70]
**Total (95% CI)**		**977**	**52.1%**	**18.56 [12.25; 25.82]**
Heterogeneity: $Tau^2 = 0.0066$; $Chi^2 = 20.4$, df = 3 (P < 0.01); $I^2 = 85\%$				
**Total (95% CI)**		**1577**	**100.0%**	**21.12 [16.49; 26.15]**
Heterogeneity: $Tau^2 = 0.0056$; $Chi^2 = 36.62$, df = 7 (P < 0.01); $I^2 = 81\%$				

© 2021 Cioana M et al. *JAMA Network Open.*

eFigure 5. Forest Plot Showing Pooled Odds Ratio of Hypertension in Male vs Female Participants With Pediatric Type 2 Diabetes

Study or Subgroup	Males Events	Males Total	Females Events	Females Total	Weight	Odds Ratio IV, Random, 95% CI
Amutha, 2012	22	81	25	117	15.1%	1.37 [0.71, 2.65]
Copeland, 2011	44	247	52	457	34.5%	1.69 [1.09, 2.61]
Eppens, 2006 (Western Pacific)	34	120	30	145	20.5%	1.52 [0.86, 2.67]
Rodriguez, 2010	38	152	59	258	29.9%	1.12 [0.70, 1.80]
**Total (95% CI)**		**600**		**977**	**100.0%**	**1.42 [1.10, 1.83]**
Total events	138		166			

Heterogeneity: $Tau^2 = 0.00$; $Chi^2 = 1.62$, df = 3 (P = 0.65); $I^2 = 0\%$

Test for overall effect: Z = 2.67 (P = 0.008)

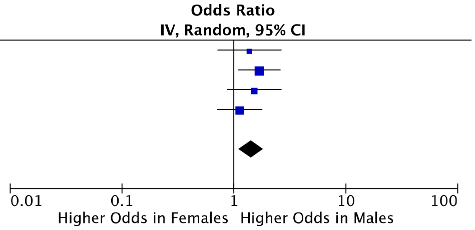

© 2021 Cioana M et al. *JAMA Network Open.*

eFigure 6. Forest Plot Showing Pooled Prevalence of Hypertension Across Different Racial Groups With Pediatric Type 2 Diabetes

Study	Cases	Total	Weight	Prevalence (%) [95% CI]
Racial Group = Caucasian				
Reinehr, 2005 (Germany)	8	16	2.7%	50.00 [25.40; 74.60]
Amed, 2012 (Canada)	16	57	5.2%	28.07 [17.07; 40.53]
Bell, 2009 (USA)	17	106	6.2%	16.04 [9.60; 23.70]
Copeland, 2011 (USA)	23	138	6.5%	16.67 [10.87; 23.39]
Khalil, 2019 (Egypt)	1	13	2.4%	7.69 [0.00; 30.13]
**Total (95% CI)**		**330**	**23.0%**	**20.95 [12.65; 30.57]**
Heterogeneity: Tau^2 = 0.0086; Chi^2 = 11.87, df = 4 (P = 0.02); I^2 = 66%				
Racial Group = African American				
Mayer-Davis, 2009 (USA)	49	212	7.0%	23.11 [17.67; 29.04]
Copeland, 2011 (USA)	34	222	7.0%	15.32 [10.86; 20.37]
**Total (95% CI)**		**434**	**14.0%**	**19.04 [12.01; 27.23]**
Heterogeneity: Tau^2 = 0.0037; Chi^2 = 4.24, df = 1 (P = 0.04); I^2 = 76%				
Racial Group = Hispanic/Latino				
Lawrence, 2009 (USA)	25	120	6.3%	20.83 [13.99; 28.60]
Copeland, 2011 (USA)	31	289	7.2%	10.73 [7.40; 14.58]
**Total (95% CI)**		**409**	**13.6%**	**15.11 [6.56; 26.30]**
Heterogeneity: Tau^2 = 0.0085; Chi^2 = 6.77, df = 1 (P < 0.01); I^2 = 85%				
Racial Group = Asian				
Urakami, 2009 (Japan)	13	112	6.2%	11.61 [6.25; 18.27]
Zabeen, 2016 (Bangladesh)	25	77	5.7%	32.47 [22.41; 43.39]
Unnikrishnan, 2008 (India)	1	36	4.3%	2.78 [0.00; 11.54]
Liu, 2009 (USA)	8	29	3.9%	27.59 [12.60; 45.47]
Amutha, 2012 (India)	47	198	6.9%	23.74 [18.05; 29.93]
**Total (95% CI)**		**452**	**27.0%**	**18.37 [9.49; 29.23]**
Heterogeneity: Tau^2 = 0.0163; Chi^2 = 25.04, df = 4 (P < 0.01); I^2 = 84%				
Racial Group = Indigenous				
Amed, 2012 (Canada)	27	100	6.1%	27.00 [18.71; 36.17]
Dabelea, 2009 (USA)	22	62	5.3%	35.48 [23.98; 47.87]
Copeland, 2011 (USA)	7	43	4.6%	16.28 [6.52; 29.01]
**Total (95% CI)**		**205**	**16.0%**	**26.48 [17.34; 36.74]**
Heterogeneity: Tau^2 = 0.0053; Chi^2 = 4.75, df = 2 (P = 0.09); I^2 = 58%				
Racial Group = Pacific Islander				
Rodriguez, 2010 (USA)	10	37	4.3%	27.03 [13.76; 42.63]
Curran, 2020 (Australia)	3	11	2.1%	27.27 [4.39; 57.92]
**Total (95% CI)**		**48**	**6.5%**	**26.71 [14.54; 40.72]**
Heterogeneity: Tau^2 = 0; Chi^2 = 0.01, df = 1 (P = 0.92); I^2 = 0%				
**Total (95% CI)**		**1878**	**100.0%**	**20.24 [16.29; 24.47]**
Heterogeneity: Tau^2 = 0.0079; Chi^2 = 72.46, df = 18 (P < 0.01); I^2 = 75%				

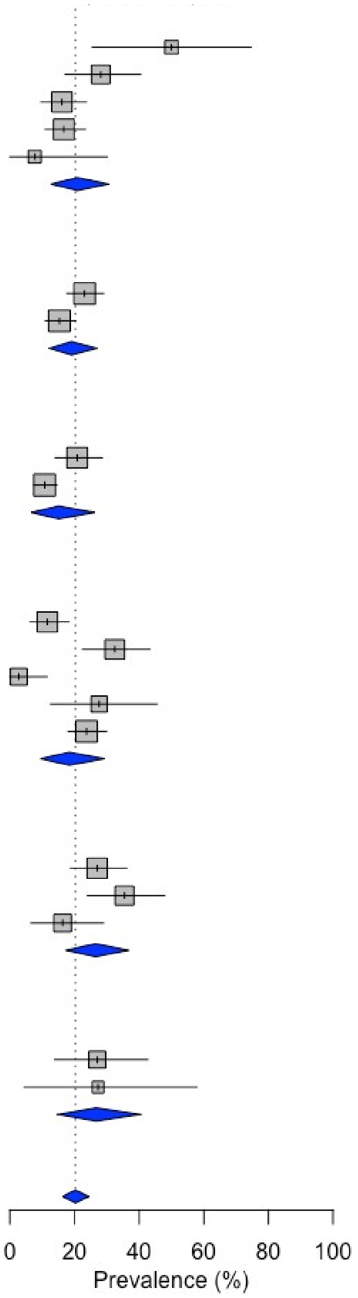

© 2021 Cioana M et al. *JAMA Network Open.*

eFigure 7. Forest Plot Showing Pooled Prevalence of Microalbuminuria in Pediatric Type 2 Diabetes

Study	Cases	Total	Weight	Prevalence (%) [95% CI]
**Study Design =** Cross-Sectional				
Hotu, 2004 (New Zealand)	5	12	3.8%	41.67 [14.90; 70.99]
Ettinger, 2005 (USA)	10	25	5.3%	40.00 [21.46; 60.04]
Eppens, 2006 (Western Pacific)	20	251	8.0%	7.97 [4.91; 11.67]
Kim, 2010 (USA)	19	103	7.4%	18.45 [11.49; 26.57]
Amutha, 2012 (India)	54	368	8.1%	14.67 [11.23; 18.48]
Zabeen, 2016 (abstract) (Bangladesh)	14	144	7.7%	9.72 [5.36; 15.16]
Nambam, 2017 (USA)	36	598	8.3%	6.02 [4.24; 8.08]
**Total (95% CI)**		**1501**	**48.6%**	**13.97 [8.44; 20.52]**
Heterogeneity: $Tau^2 = 0.0097$; $Chi^2 = 49.12$, df = 6 ($P < 0.01$); $I^2 = 88\%$				
**Study Design =** Retrospective Cohort				
Le, 2013 (USA)	11	64	6.9%	17.19 [8.80; 27.52]
Osman, 2013 (Sudan)	7	38	6.1%	18.42 [7.45; 32.54]
Haynes, 2014 (Australia)	11	61	6.8%	18.03 [9.26; 28.79]
Calagua Quipse, 2015 (Peru)	9	20	4.9%	45.00 [23.57; 67.36]
Newton, 2015 (New Zealand)	6	11	3.7%	54.55 [24.24; 83.33]
Son, 2015 (Korea)	8	18	4.7%	44.44 [21.96; 68.07]
Yeow, 2019 (Malaysia)	7	24	5.2%	29.17 [12.42; 49.17]
**Total (95% CI)**		**236**	**38.2%**	**28.00 [18.32; 38.74]**
Heterogeneity: $Tau^2 = 0.0127$; $Chi^2 = 15.87$, df = 6 ($P = 0.01$); $I^2 = 62\%$				
**Study Design =** Prospective Cohort				
Ruhayel, 2010 (Australia)	9	20	4.9%	45.00 [23.57; 67.36]
Schmidt, 2012 (Germany and Austria)	154	684	8.3%	22.51 [19.46; 25.72]
**Total (95% CI)**		**704**	**13.2%**	**30.65 [11.39; 54.05]**
Heterogeneity: $Tau^2 = 0.0232$; $Chi^2 = 4.69$, df = 1 ($P = 0.03$); $I^2 = 79\%$				
**Total (95% CI)**		**2441**	**100.0%**	**21.57 [15.59; 28.16]**
Heterogeneity: $Tau^2 = 0.0166$; $Chi^2 = 148.41$, df = 15 ($P < 0.01$); $I^2 = 90\%$				

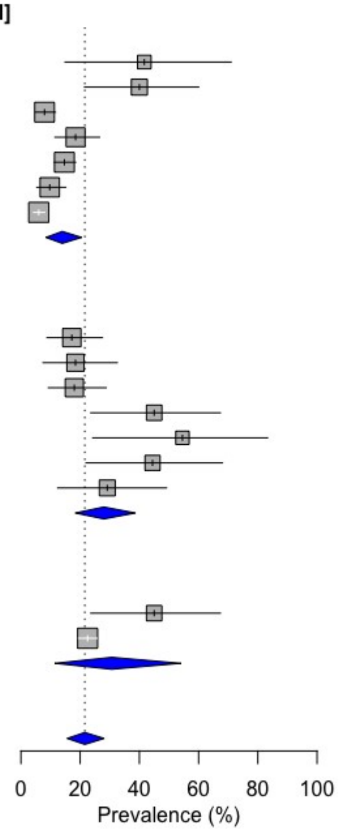

© 2021 Cioana M et al. *JAMA Network Open.*

eFigure 8. Forest Plot Showing Pooled Prevalence of Persistent Microalbuminuria in Pediatric Type 2 Diabetes

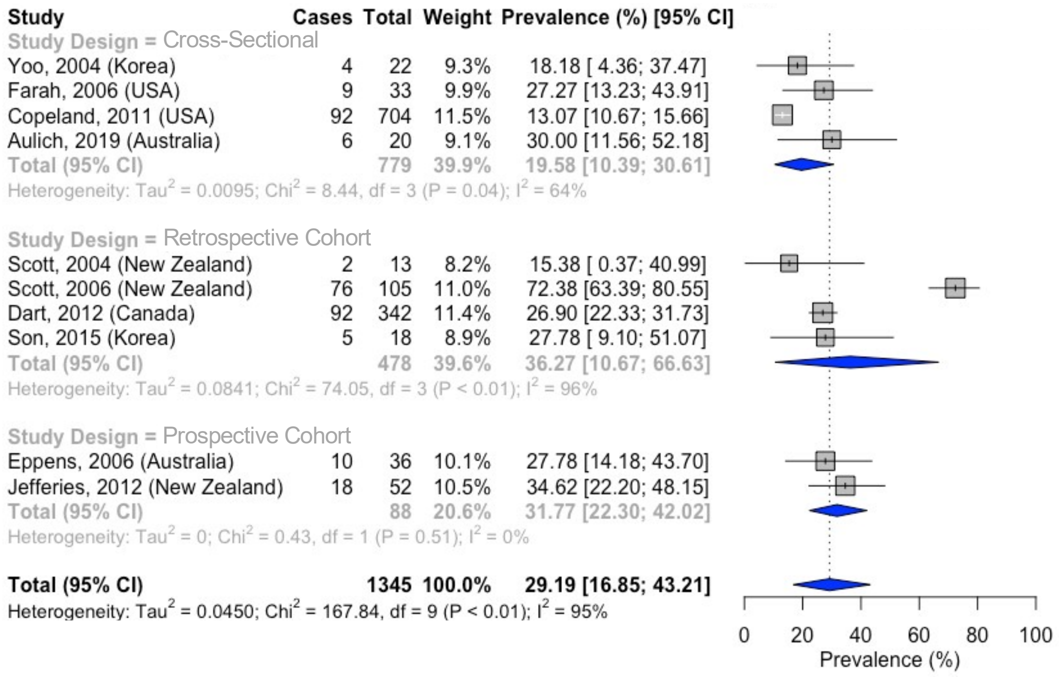

© 2021 Cioana M et al. *JAMA Network Open.*

eFigure 9. Forest Plot Showing Pooled Prevalence of Macroalbuminuria in Pediatric Type 2 Diabetes

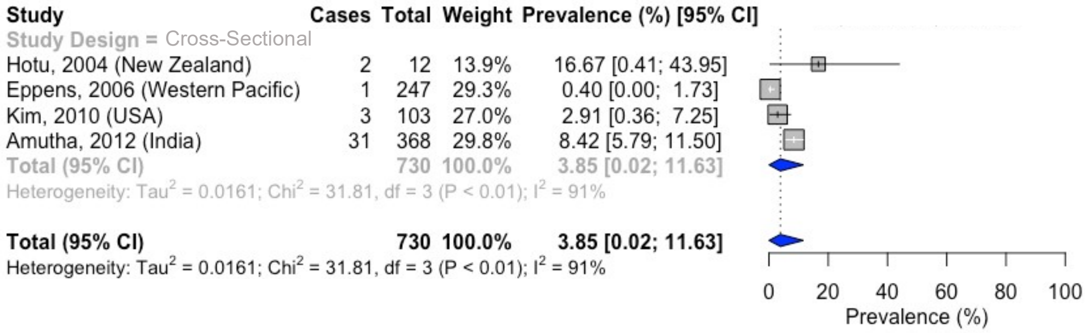

© 2021 Cioana M et al. *JAMA Network Open.*

eFigure 10. Forest Plot Showing Pooled Prevalence of Persistent Albuminuria in Pediatric Type 2 Diabetes by Sex

Study	Cases	Total	Weight	Prevalence (%) [95% CI]
Sex = Male				
Copeland, 2011 (USA)	26	247	26.4%	10.53 [6.98; 14.69]
Dart, 2019 (Canada)	15	62	21.7%	24.19 [14.25; 35.72]
**Total (95% CI)**		**309**	**48.1%**	**16.14 [5.05; 31.65]**
Heterogeneity: $Tau^2 = 0.0148$; $Chi^2 = 6.9$, df = 1 (P < 0.01); $I^2 = 86\%$				
Sex = Female				
Copeland, 2011 (USA)	65	457	27.3%	14.22 [11.16; 17.58]
Dart, 2019 (Canada)	42	125	24.6%	33.60 [25.56; 42.15]
**Total (95% CI)**		**582**	**51.9%**	**22.90 [7.08; 44.22]**
Heterogeneity: $Tau^2 = 0.0256$; $Chi^2 = 21.18$, df = 1 (P < 0.01); $I^2 = 95\%$				
**Total (95% CI)**		**891**	**100.0%**	**19.39 [10.90; 29.56]**
Heterogeneity: $Tau^2 = 0.0126$; $Chi^2 = 31.74$, df = 3 (P < 0.01); $I^2 = 91\%$				

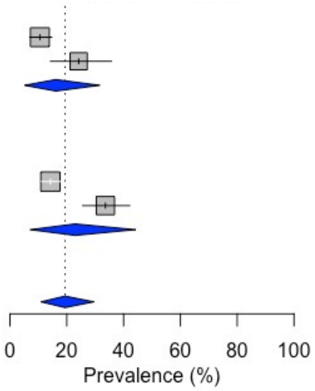

© 2021 Cioana M et al. *JAMA Network Open.*

eFigure 11. Forest Plot Showing Pooled Odds Ratio of Persistent Albuminuria in Male vs Female Participants With Pediatric Type 2 Diabetes

Study or Subgroup	Males Events	Males Total	Females Events	Females Total	Weight	Odds Ratio IV, Random, 95% CI
Dart, 2019	15	62	42	125	33.0%	0.63 [0.32, 1.26]
Copeland, 2011	26	247	65	457	67.0%	0.71 [0.44, 1.15]
**Total (95% CI)**		**309**		**582**	**100.0%**	**0.68 [0.46, 1.01]**
Total events	41		107			

Heterogeneity: $Tau^2 = 0.00$; $Chi^2 = 0.08$, df = 1 (P = 0.78); $I^2 = 0\%$
Test for overall effect: Z = 1.89 (P = 0.06)

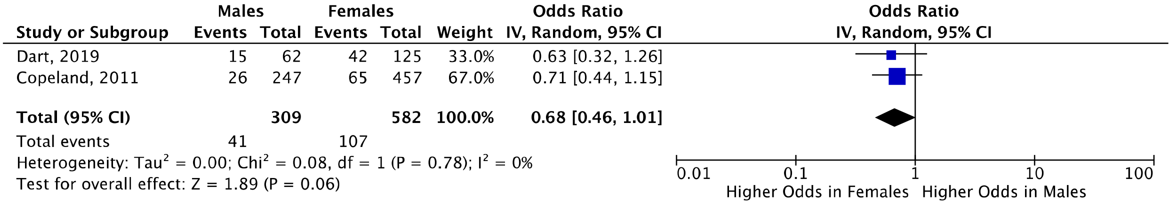

© 2021 Cioana M et al. *JAMA Network Open.*

eFigure 12. Forest Plot Showing Pooled Prevalence of Albuminuria Across Different Racial Groups With Pediatric Type 2 Diabetes

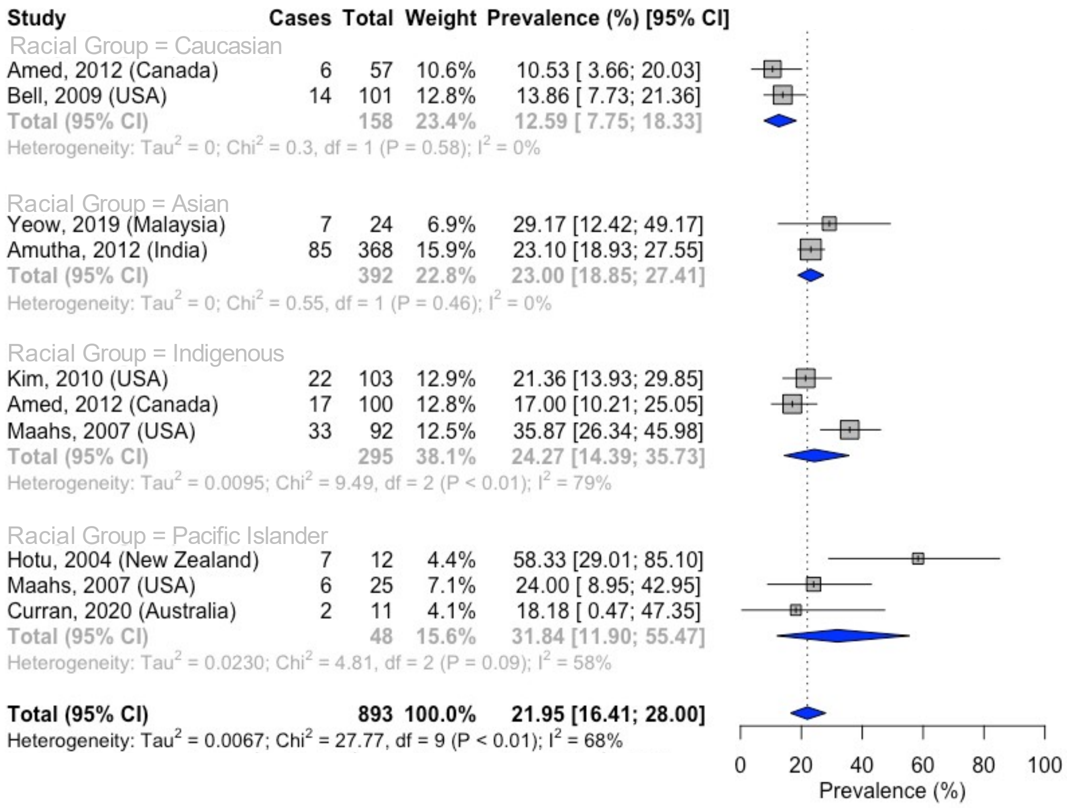

© 2021 Cioana M et al. *JAMA Network Open.*

eFigure 13. Forest Plot Showing Pooled Prevalence of Persistent Albuminuria Across Different Racial Groups With Pediatric Type 2 Diabetes

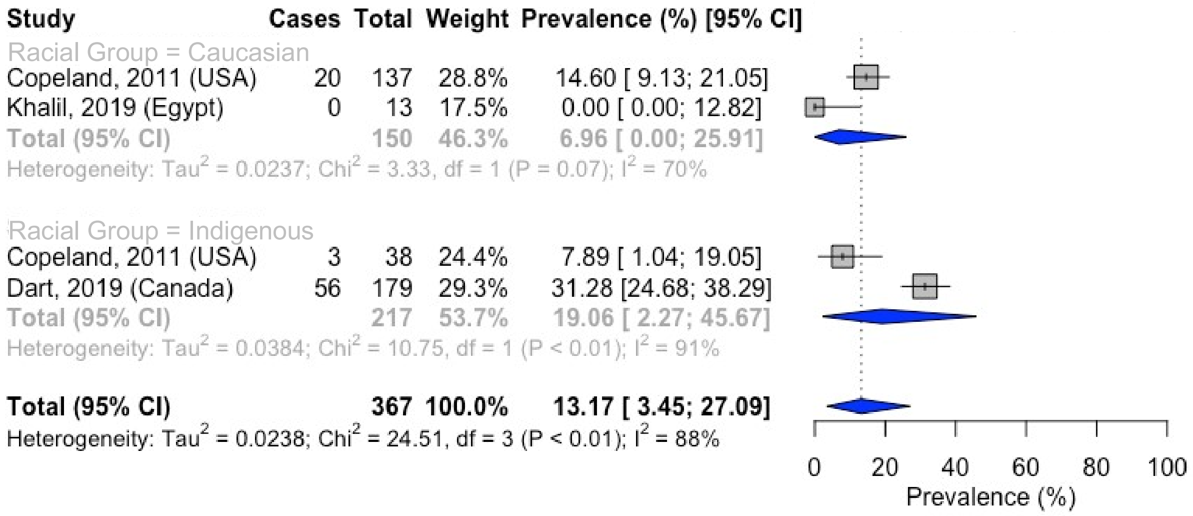

© 2021 Cioana M et al. *JAMA Network Open.*

eFigure 14. Forest Plot Showing Pooled Prevalence of Microalbuminuria in Asian Patients with Pediatric Type 2 Diabetes

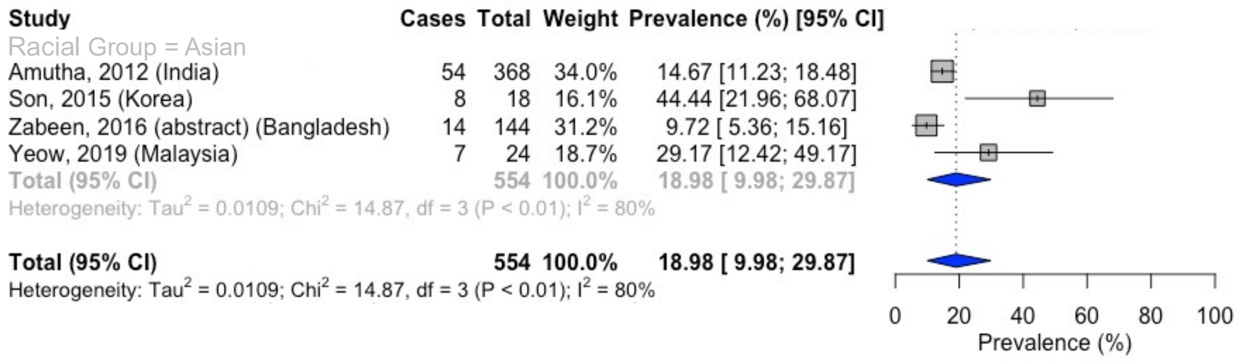

© 2021 Cioana M et al. *JAMA Network Open.*

eFigure 15. Forest Plot Showing Pooled Prevalence of Persistent Microalbuminuria in Asian Patients with Pediatric Type 2 Diabetes

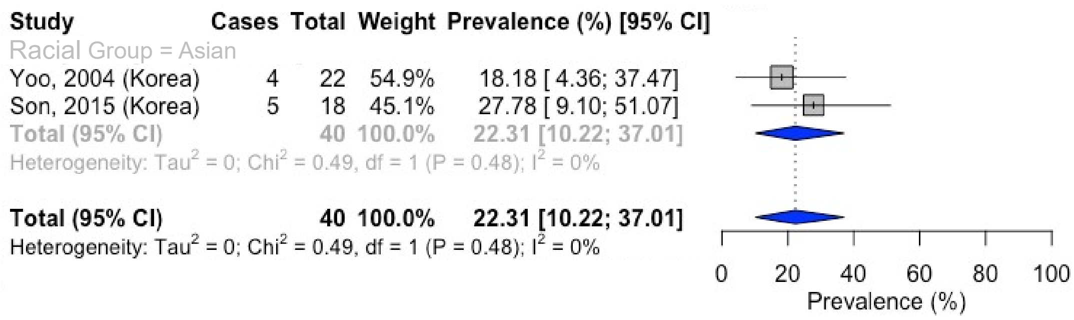

© 2021 Cioana M et al. *JAMA Network Open.*

eFigure 16. Funnel Plot Examining Publication Bias for Pooled Prevalence of Hypertension Outcome

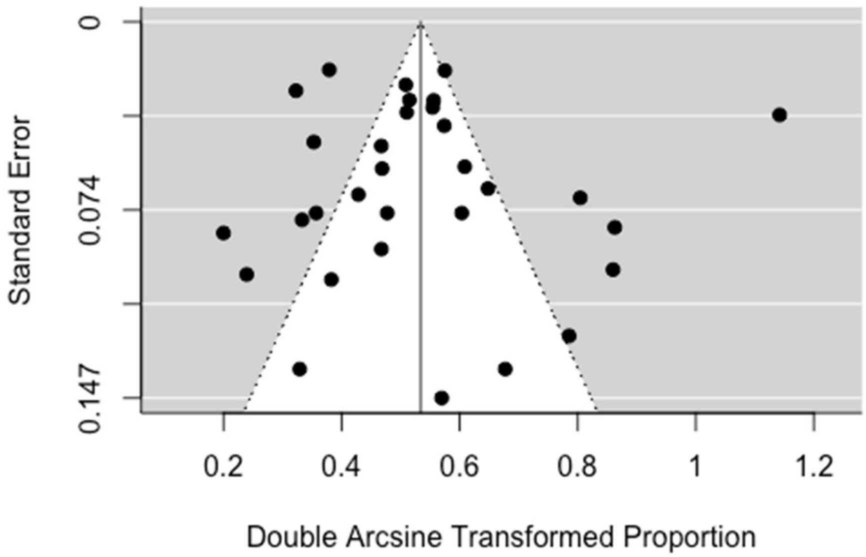

© 2021 Cioana M et al. *JAMA Network Open.*

eFigure 17. Funnel Plot Examining Publication Bias for Pooled Prevalence of Albuminuria Outcome

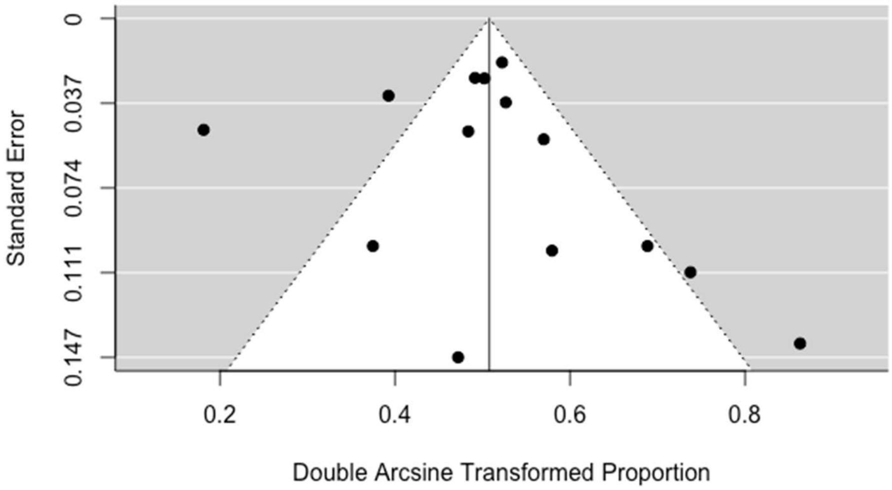

© 2021 Cioana M et al. *JAMA Network Open.*

eFigure 18. Funnel Plot Examining Publication Bias for Pooled Prevalence of Persistent Albuminuria Outcome

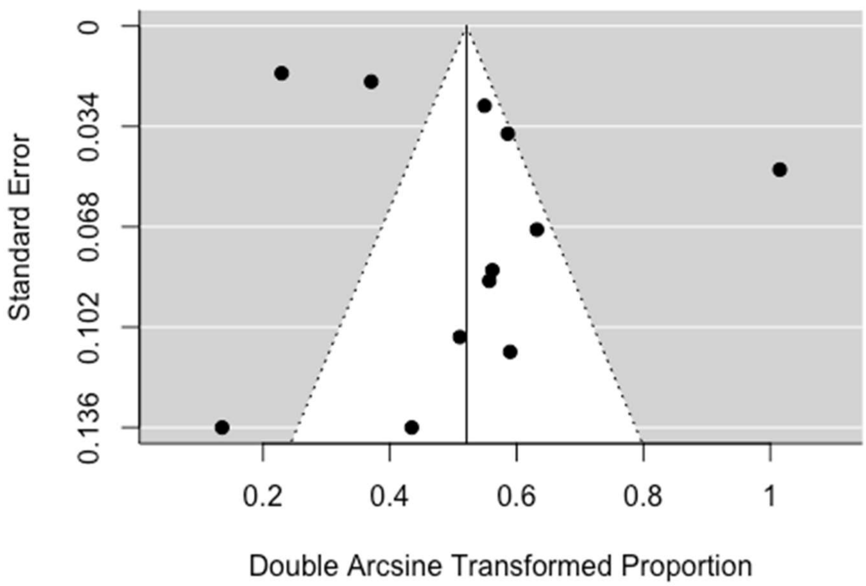

eFigure 19. Funnel Plot Examining Publication Bias for Pooled Prevalence of Microalbuminuria Outcome

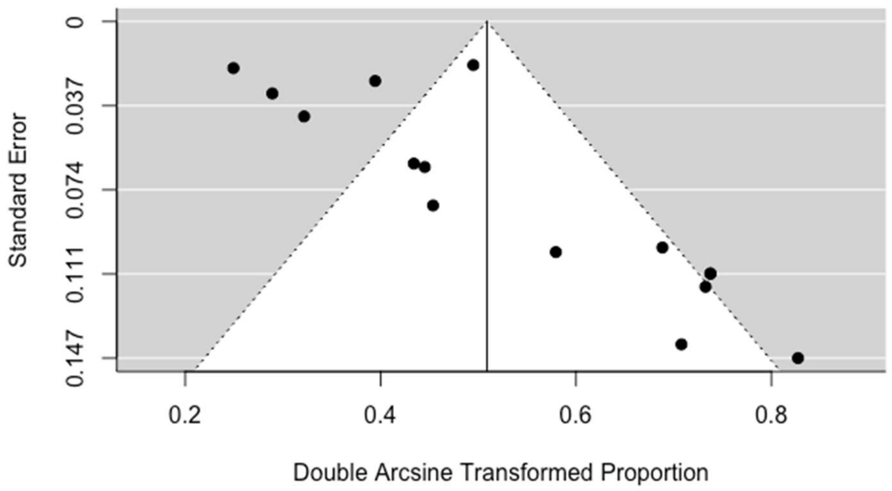

eFigure 20. Funnel Plot Examining Publication Bias for Pooled Prevalence of Persistent Microalbuminuria Outcome

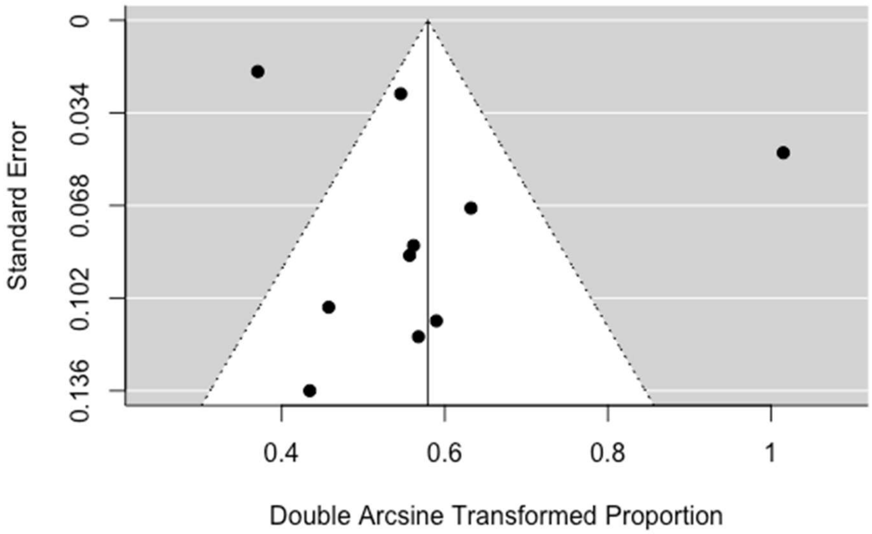

eFigure 21. Distribution of Risk of Bias Sources in the Included Studies

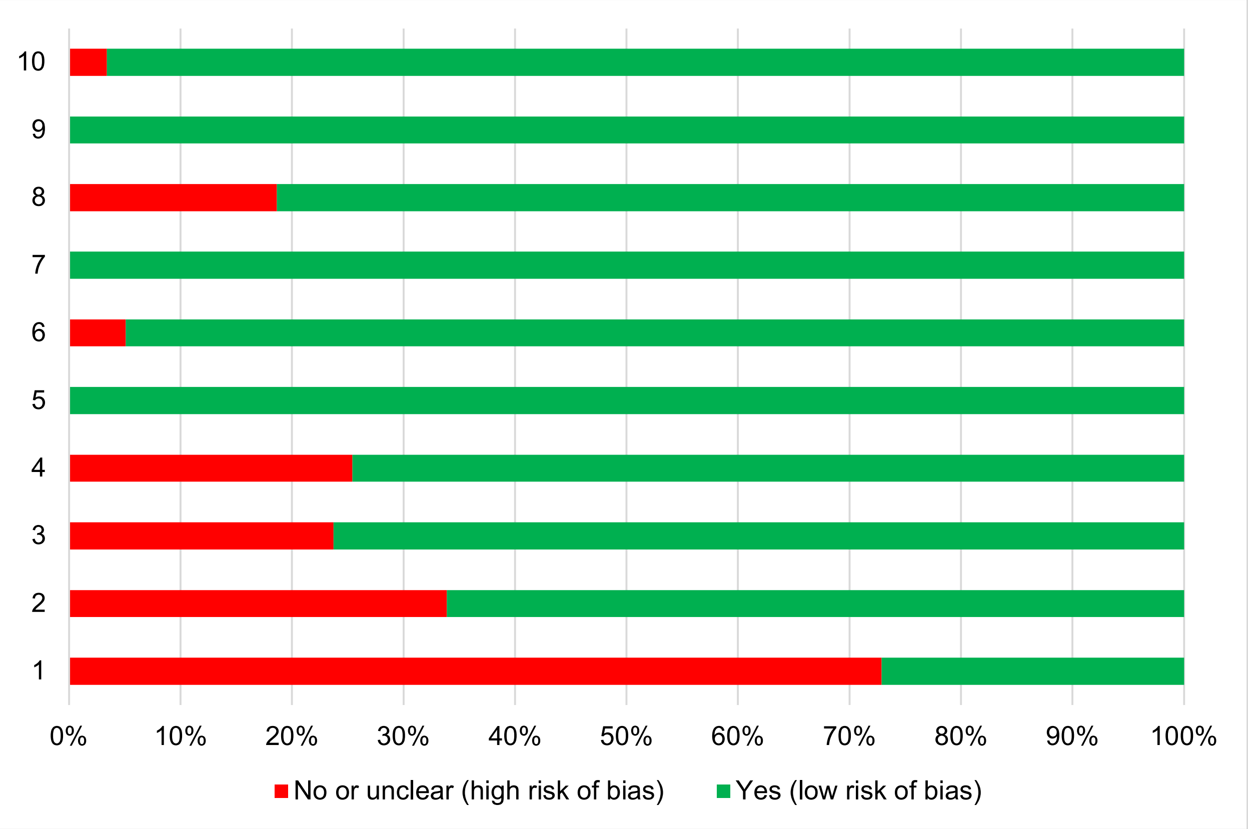

Footnote: Overall risk of bias: low (score >8), moderate (score 6-8), or high (score ≤5). Items scored: 1) Was the study's target population a close representation of the national population in relation to relevant variables, e.g., age, sex?; 2) Was the sampling frame a true or close representation of the target population?; 3) Was some form of random selection used to select the sample, OR, was a census undertaken?; 4) Was the likelihood of non-response bias minimal?; 5) Were data collected directly from the subjects (as opposed to a proxy)?; 6) Was an acceptable case definition used in the study?; 7) Had the study instrument that measured the parameter of interest (e.g., prevalence of comorbidity) been tested for reliability and validity (if necessary)?; 8) Was the same mode of data collection used for all subjects?; 9) Was the length of the shortest prevalence period for the parameter of interest appropriate?; 10) Were the numerator(s) and denominator(s) for the parameter of interest appropriate?

© 2021 Cioana M et al. *JAMA Network Open.*

eAppendix. List of Studies Excluded at the Full-Text Screening Stage

Exclusion Reason: Adult (n = 66)

1. Al-Saeed AH, Constantino MI, Molyneaux L, et al. An Inverse Relationship Between Age of Type 2 Diabetes Onset and Complication Risk and Mortality: The Impact of Youth-Onset Type 2 Diabetes. *Diabetes Care*. 2016;39(5):823-829. doi:10.2337/dc15-0991

2. Baena Díez J, Martínez Martínez J, Alvarez Pérez B, et al. [Cardiovascular risk and the new diagnostic categories for diabetes mellitus proposed by the American Diabetes Association]. *Aten Primaria*. 2001;28(1):31-38. doi:10.1016/s0212-6567(01)78892-0

3. Brandt M, Harder K, Walluscheck KP, Fraund S, Böning A, Cremer J. Coronary artery bypass surgery in diabetic patients. *J Card Surg*. 2004;19(1):36-40. doi:10.1111/j.0886-0440.2004.04007.x

4. Calhoun D, Beals J, Carter EA, et al. Relationship between glycemic control and depression among American Indians in the Strong Heart Study. *J Diabetes Complications*. 2010;24(4):217-222. doi:10.1016/j.jdiacomp.2009.03.005

5. Chen H-S, Wu T-EJ, Jap T-S, et al. Subclinical hypothyroidism is a risk factor for nephropathy and cardiovascular diseases in Type 2 diabetic patients. *Diabet Med*. 2007;24(12):1336-1344. doi:10.1111/j.1464-5491.2007.02270.x

6. Cheng Y-Y, Leu H-B, Chen T-J, et al. Metformin-inclusive therapy reduces the risk of stroke in patients with diabetes: a 4-year follow-up study. *J Stroke Cerebrovasc Dis*. 2014;23(2):e99-e105. doi:10.1016/j.jstrokecerebrovasdis.2013.09.001

7. Chuang LM, Tsai ST, Huang BY, Tai TY, DIABCARE (Taiwan) Study Group. The current state of diabetes management in Taiwan. *Diabetes Res Clin Pract*. 2001;54 Suppl 1:S55-S65. doi:10.1016/s0168-8227(01)00310-2

8. Cisse A, Lopez Sall P, Diop PA, et al. [Frequency of microalbuminuria during diabetes in Dakar, Senegal]. *Dakar Med*. 2003;48(3):237-239. https://www.ncbi.nlm.nih.gov/pubmed/15776639

9. Constantino MI, Molyneaux L, Limacher-Gisler F, et al. Long-term complications and mortality in young-onset diabetes: type 2 diabetes is more hazardous and lethal than type 1 diabetes. *Diabetes Care*. 2013;36(12):3863-3869. doi:10.2337/dc12-2455

10. Coppell K, Williams S, Anderson K, Lamb C, Mann J. Characteristics and cardiovascular risk of new cases of type 2 diabetes in Otago, New Zealand, 1998-2004. *Diabetes Res Clin Pract*. 2008;82(3):396-401. doi:10.1016/j.diabres.2008.09.038

11. Craig KJ, Donovan K, Munnery M, Owens DR, Williams JD, Phillips AO. Identification and management of diabetic nephropathy in the diabetes clinic. *Diabetes Care*. 2003;26(6):1806-1811. doi:10.2337/diacare.26.6.1806

12. Cuellar NG, Ratcliffe SJ. Restless legs syndrome in type 2 diabetes: implications to diabetes educators. *Diabetes Educ*. 2008;34(2):218-234. doi:10.1177/0145721708314180

13. D'Silva LJ, Staecker H, Lin J, et al. Retrospective data suggests that the higher prevalence of benign paroxysmal positional vertigo in individuals with type 2 diabetes is mediated by hypertension. *J Vestib Res*. 2016;25(5-6):233-239. doi:10.3233/VES-150563

14. da Mata AR, Álvares J, Diniz LM, et al. Quality of life of patients with Diabetes Mellitus Types 1 and 2 from a referal health centre in Minas Gerais, Brazil. *Expert Rev Clin Pharmacol*. 2016;9(5):739-746. doi:10.1586/17512433.2016.1152180

© 2021 Cioana M et al. *JAMA Network Open.*

15. Dabelea D, Stafford JM, Mayer-Davis EJ, et al. Association of Type 1 Diabetes vs Type 2 Diabetes Diagnosed During Childhood and Adolescence With Complications During Teenage Years and Young Adulthood. *JAMA*. 2017;317(8):825-835. doi:10.1001/jama.2017.0686

16. Donaghue KC, Fairchild JM, Craig ME, et al. Do all prepubertal years of diabetes duration contribute equally to diabetes complications? *Diabetes Care*. 2003;26(4):1224-1229. doi:10.2337/diacare.26.4.1224

17. Drion I, Kleefstra N, Landman GWD, et al. Plasma COOH-terminal proendothelin-1: a marker of fatal cardiovascular events, all-cause mortality, and new-onset albuminuria in type 2 diabetes? (ZODIAC-29). *Diabetes Care*. 2012;35(11):2354-2358. doi:10.2337/dc11-2526

18. Durruty P, Carpentier C, Krause P, García de los Ríos M. [Evaluation of retinal involvement in type 2 diabetics with microalbuminuria]. *Rev Med Chil*. 2000;128(10):1085-1092. https://www.ncbi.nlm.nih.gov/pubmed/11349506

19. Estacio RO, Dale RA, Schrier R, Krantz MJ. Relation of reduction in urinary albumin excretion to ten-year cardiovascular mortality in patients with type 2 diabetes and systemic hypertension. *Am J Cardiol*. 2012;109(12):1743-1748. doi:10.1016/j.amjcard.2012.02.020

20. Girach A, Vignati L. Diabetic microvascular complications--can the presence of one predict the development of another? *J Diabetes Complications*. 2006;20(4):228-237. doi:10.1016/j.jdiacomp.2006.03.001

21. Hannon TS, Gungor N, Arslanian SA. Type 2 diabetes in children and adolescents: a review for the primary care provider. *Pediatr Ann*. 2006;35(12):880-887. doi:10.3928/0090-4481-20061201-02

22. Heisler M, Smith DM, Hayward RA, Krein SL, Kerr EA. Racial disparities in diabetes care processes, outcomes, and treatment intensity. *Med Care*. 2003;41(11):1221-1232. doi:10.1097/01.MLR.0000093421.64618.9C

23. Hernández-Ávila M, Gutiérrez JP, Reynoso-Noverón N. [Diabetes mellitus in Mexico. Status of the epidemic]. *Salud Publica Mex*. 2013;55 Suppl 2:S129-S136. https://www.ncbi.nlm.nih.gov/pubmed/24626688

24. Ionescu-Tîrgovişte C, Paterache E, Cheţa D, Farcaşiu E, Serafinceanu C, Mincu I. Epidemiology of diabetes in Bucharest. *Diabet Med*. 1994;11(4):413-417. doi:10.1111/j.1464-5491.1994.tb00296.x

25. Jin K-K, Chen L, Pan J-Y, Li J-M, Wang Y, Wang F-Y. Acupressure therapy inhibits the development of diabetic complications in Chinese patients with type 2 diabetes. *J Altern Complement Med*. 2009;15(9):1027-1032. doi:10.1089/acm.2008.0608

26. Lascar N, Brown J, Pattison H, Barnett AH, Bailey CJ, Bellary S. Type 2 diabetes in adolescents and young adults. *Lancet Diabetes Endocrinol*. 2018;6(1):69-80. doi:10.1016/S2213-8587(17)30186-9

27. Maracy MR, Feizi A, Bagherynejad M. The prevalence and correlated determinants of hypertension and type 2 diabetes: A large community-based study in Isfahan, Iran. *Pak J Med Sci*. 2012;28(2):247-252.

28. McGrath NM, Parker GN, Dawson P. Early presentation of type 2 diabetes mellitus in young New Zealand Maori. *Diabetes Res Clin Pract*. 1999;43(3):205-209. doi:10.1016/s0168-8227(99)00003-0

29. Moreira TMM, Gomes EB, dos Santos JC. [Cardiovascular risk factors in young adults with arterial hypertension and/or diabetes mellitus]. *Rev Gaucha Enferm*. 2010;31(4):662-669. doi:10.1590/s1983-14472010000400008

30. Moss SE, Klein R, Klein BE. Ten-year incidence of visual loss in a diabetic population. *Ophthalmology*. 1994;101(6):1061-1070. doi:10.1016/s0161-6420(94)31217-6

31. Nelson RG, Knowler WC, Pettitt DJ, Hanson RL, Bennett PH. Incidence and determinants of elevated urinary albumin excretion in Pima Indians with NIDDM. *Diabetes Care*. 1995;18(2):182-187. doi:10.2337/diacare.18.2.182

© 2021 Cioana M et al. *JAMA Network Open.*

32. Nelson RG, Pettitt DJ, Baird HR, et al. Pre-diabetic blood pressure predicts urinary albumin excretion after the onset of type 2 (non-insulin-dependent) diabetes mellitus in Pima Indians. *Diabetologia*. 1993;36(10):998-1001. doi:10.1007/BF02374490

33. Pavkov ME, Mason CC, Bennett PH, Curtis JM, Knowler WC, Nelson RG. Change in the distribution of albuminuria according to estimated glomerular filtration rate in Pima Indians with type 2 diabetes. *Diabetes Care*. 2009;32(10):1845-1850. doi:10.2337/dc08-2325

34. Penno G, Solini A, Bonora E, et al. HbA1c variability as an independent correlate of nephropathy, but not retinopathy, in patients with type 2 diabetes: the Renal Insufficiency And Cardiovascular Events (RIACE) Italian multicenter study. *Diabetes Care*. 2013;36(8):2301-2310. doi:10.2337/dc12-2264

35. Rahman S, Nawaz R, Khan GJ, Aamir AH. Frequency of diabetic retinopathy in hypertensive diabetic patients in a tertiary care hospital of Peshawar, Pakistan. *J Ayub Med Coll Abbottabad*. 2011;23(2):133-135. https://www.ncbi.nlm.nih.gov/pubmed/24800364

36. Ramaiya KL, Swai AB, McLarty DG, Alberti KG. Impaired glucose tolerance and diabetes mellitus in Hindu Indian immigrants in Dar es Salaam. *Diabet Med*. 1991;8(8):738-744. doi:10.1111/j.1464-5491.1991.tb01693.x

37. Sayeed MA, Hussain MZ, Banu A, Rumi MA, Azad Khan AK. Prevalence of diabetes in a suburban population of Bangladesh. *Diabetes Res Clin Pract*. 1997;34(3):149-155. doi:10.1016/s0168-8227(96)01337-x

38. Schiel R, Müller UA, Beltschikow W, Stein G. Trends in the management of arterial hypertension in patients with type 1 and insulin-treated type 2 diabetes mellitus over a period of 10 years (1989/1990-1994/1995). Results of the JEVIN trial. *J Diabetes Complications*. 2006;20(5):273-279. doi:10.1016/j.jdiacomp.2005.07.011

39. Seyum B, Mebrahtu G, Usman A, et al. Profile of patients with diabetes in Eritrea: results of first phase registry analyses. *Acta Diabetol*. 2010;47(1):23-27. doi:10.1007/s00592-009-0093-8

40. Shivananda Nayak B, Duncan H, Lalloo S, et al. Correlation of microalbumin and sialic acid with anthropometric variables in type 2 diabetic patients with and without nephropathy. *Vasc Health Risk Manag*. 2008;4(1):243-247. doi:10.2147/vhrm.2008.04.01.243

41. So W-Y, Raboca J, Sobrepena L, et al. Comprehensive risk assessments of diabetic patients from seven Asian countries: The Joint Asia Diabetes Evaluation (JADE) program. *J Diabetes*. 2011;3(2):109-118. doi:10.1111/j.1753-0407.2011.00115.x

42. Sterling SA, Jones AE, Cox RD. Longitudinal Trends in the Prevalence of Diabetes Mellitus in an Urban Emergency Department. *South Med J*. 2016;109(4):222-227. doi:10.14423/SMJ.0000000000000447

43. Svensson M, Sundkvist G, Arnqvist HJ, et al. Signs of nephropathy may occur early in young adults with diabetes despite modern diabetes management: results from the nationwide population-based Diabetes Incidence Study in Sweden (DISS). *Diabetes Care*. 2003;26(10):2903-2909. doi:10.2337/diacare.26.10.2903

44. Tseng C-H, Chong C-K, Tseng C-P, Shau W-Y, Tai T-Y. Hypertension is the most important component of metabolic syndrome in the association with ischemic heart disease in Taiwanese type 2 diabetic patients. *Circ J*. 2008;72(9):1419-1424. doi:10.1253/circj.cj-08-0009

45. Yardley JE, MacMillan F, Hay J, et al. The blood pressure response to exercise in youth with impaired glucose tolerance and type 2 diabetes. *Pediatr Exerc Sci*. 2015;27(1):120-127. doi:10.1123/pes.2014-0062

46. Yokoyama H, Okudaira M, Otani T, et al. Higher incidence of diabetic nephropathy in type 2 than in type 1 diabetes in early-onset diabetes in Japan. *Kidney Int*. 2000;58(1):302-311. doi:10.1046/j.1523-1755.2000.00166.x

47. Public Health Foundation of India. A Multi-site, Individually Randomized, Controlled Translation Trial of Integrated and Comprehensive Care Strategies to Reduce Cardiovascular Disease (CVD) Risk Among 1,120

© 2021 Cioana M et al. *JAMA Network Open.*

Type 2 Diabetes Mellitus(T2DM) Patients in South Asia. ClinicalTrials.gov. Published 2010. Accessed February 11, 2021. https://clinicaltrials.gov/ct2/show/NCT01212328

48. Nordsjaellands Hospital. Impact of CPAP Treatment on Arterial Stiffness in Patients With T2DM and Newly Diagnosed Obstructive Sleep Apnoea. ClinicalTrials.gov. Published 2015. Accessed February 11, 2021. https://clinicaltrials.gov/ct2/show/NCT02482584

49. Afkhami-Ardekani M, Modarresi M, Amirchaghmaghi E. Prevalence of microalbuminuria and its risk factors in type 2 diabetic patients. *Indian J Nephrol*. 2008;18(3):112-117. doi:10.4103/0971-4065.43690

50. Al Rawahi AH, Lee P, Al Anqoudi ZAM, et al. Cardiovascular Disease Incidence and Risk Factor Patterns among Omanis with Type 2 Diabetes: A Retrospective Cohort Study. *Oman Med J*. 2017;32(2):106-114. doi:10.5001/omj.2017.20

51. Bardini G, Innocenti M, Rotella CM, Giannini S, Mannucci E. Variability of triglyceride levels and incidence of microalbuminuria in type 2 diabetes. *J Clin Lipidol*. 2016;10(1):109-115. doi:10.1016/j.jacl.2015.10.001

52. Ke C, Sohal P, Qian H, Quan H, Khan NA. Diabetes in the young: a population-based study of South Asian, Chinese and White people. *Diabet Med*. 2015;32(4):487-496. doi:10.1111/dme.12657

53. Vides H, Nilsson PM, Sarapuu V, Podar T, Isacsson A, Scherstén BF. Diabetes and social conditions in Estonia. A population-based study. *Eur J Public Health*. 2001;11(1):60-64. doi:10.1093/eurpub/11.1.60

54. Das G, Taylor PN, Abusahmin H, et al. Relationship between serum thyrotropin and urine albumin excretion in euthyroid subjects with diabetes. *Ann Clin Biochem*. 2019;56(1):155-162. doi:10.1177/0004563218797979

55. Fan H, Wang J, Gu X. Association between social determinants and the presence of essential hypertension in type 2 diabetes mellitus patients. *Aust J Prim Health*. 2019;25(2):146-151. doi:10.1071/PY18091

56. Anandh U, Madhumita V, Manjunath S, Garg N, Sathyanarayana S. MON-295 DIABETIC KIDNEY DISEASE (DKD) IN INDIA: LESSONS LEARNT FROM A PROSPECTIVE FOLLOW UP STUDY OF DIABETES MELLITUS(DM) PATIENTS OVER 30 YEARS. *Kidney Int Rep*. 2019;4(7):S420. doi:10.1016/j.ekir.2019.05.1104

57. Araneta MR. Engaging the ASEAN Diaspora: Type 2 Diabetes Prevalence, Pathophysiology, and Unique Risk Factors among Filipino Migrants in the United States. *J ASEAN Fed Endocr Soc*. 2019;34(2):126-133. doi:10.15605/jafes.034.02.02

58. Hossain ME, Uddin S, Khan A, Moni MA. A Framework to Understand the Progression of Cardiovascular Disease for Type 2 Diabetes Mellitus Patients Using a Network Approach. *Int J Environ Res Public Health*. 2020;17(2). doi:10.3390/ijerph17020596

59. Mannam M, Nalluri L, Pinnika D, et al. A cross-sectional observational study on drug utilisation pattern, prevalence and risk factors for the development of diabetic nephropathy among type 2 diabetic patients in a south indian tertiary care hospital. *Int J Res Pharm Sci*. 2020;11(1):93-108. doi:10.26452/ijrps.v11i1.1791

60. Mkuu RS, Gilreath TD, Wekullo C, Reyes GA, Harvey IS. Social determinants of hypertension and type-2 diabetes in Kenya: A latent class analysis of a nationally representative sample. *PLoS One*. 2019;14(8):e0221257. doi:10.1371/journal.pone.0221257

61. Murayama H, Toda M, Tsumiyama I, et al. Relationship of patient background with macro- and microvascular complications: a 2-year post-marketing surveillance of vildagliptin in nearly 20,000 Japanese diabetic patients. *Expert Opin Pharmacother*. 2019;20(8):1037-1047. doi:10.1080/14656566.2019.1585802

62. Wang J, Zhao L, Zhang J, et al. Clinicopathologic features and prognosis of type 2 diabetes mellitus and diabetic nephropathy in different age groups: more attention to younger patients. *Endocr Pract*. 2020;26(1):51-57. doi:10.4158/EP-2019-0238

© 2021 Cioana M et al. *JAMA Network Open.*

63. Amin RF, El Bendary AS, Ezzat SE, Mohamed WS. Serum Ferritin level, microalbuminuria and non-alcoholic fatty liver disease in type 2 diabetic patients. *Diabetes Metab Syndr*. 2019;13(3):2226-2229. doi:10.1016/j.dsx.2019.05.030

64. Koye DN, Magliano DJ, Reid CM, et al. Trends in Incidence of ESKD in People With Type 1 and Type 2 Diabetes in Australia, 2002-2013. *Am J Kidney Dis*. 2019;73(3):300-308. doi:10.1053/j.ajkd.2018.10.005

65. Dailey GE 3rd, Mohideen P, Fiedorek FT. Lipid effects of glyburide/metformin tablets in patients with type 2 diabetes mellitus with poor glycemic control and dyslipidemia in an open-label extension study. *Clin Ther*. 2002;24(9):1426-1438. doi:10.1016/s0149-2918(02)80046-7

66. Grant RW, Meigs JB. Prevalence and treatment of low HDL cholesterol among primary care patients with type 2 diabetes: an unmet challenge for cardiovascular risk reduction. *Diabetes Care*. 2007;30(3):479-484. doi:10.2337/dc06-1961

Exclusion Reason: No Hypertension/Albuminuria Prevalence (n = 59)

1. Abusrewil SS. Epidemiology of childhood diabetes in western part of Libya (1989-2013). *Pediatr Diabetes*. 2013;14(S18):117.

2. Alberti G, Zimmet P, Shaw J, et al. Type 2 diabetes in the young: the evolving epidemic: the international diabetes federation consensus workshop. *Diabetes Care*. 2004;27(7):1798-1811. doi:10.2337/diacare.27.7.1798

3. Amed S, Dean HJ, Panagiotopoulos C, et al. Type 2 diabetes, medication-induced diabetes, and monogenic diabetes in Canadian children: a prospective national surveillance study. *Diabetes Care*. 2010;33(4):786-791. doi:10.2337/dc09-1013

4. Bacha F, Gidding SS, Caprio S, Weinstock R, Lynch J, Hirst K. High prevalence and rapid increase of cardiovascular disease risk factors in youth with type 2 diabetes: The today study group. *Circulation*. 2013;127(12 (Meeting Abstracts)).

5. Bacha F, Gidding SS, Pyle L, et al. Relationship of Cardiac Structure and Function to Cardiorespiratory Fitness and Lean Body Mass in Adolescents and Young Adults with Type 2 Diabetes. *J Pediatr*. 2016;177:159-166.e1. doi:10.1016/j.jpeds.2016.06.048

6. Barnes TL, Crandell JL, Bell RA, Mayer-Davis EJ, Dabelea D, Liese AD. Change in DASH diet score and cardiovascular risk factors in youth with type 1 and type 2 diabetes mellitus: The SEARCH for Diabetes in Youth Study. *Nutr Diabetes*. 2013;3:e91. doi:10.1038/nutd.2013.32

7. Bhatia V, Arya V, Dabadghao P, et al. Etiology and outcome of childhood and adolescent diabetes mellitus in North India. *J Pediatr Endocrinol Metab*. 2004;17(7):993-999. doi:10.1515/jpem.2004.17.7.993

8. Black MH, Anderson A, Bell RA, et al. Prevalence of asthma and its association with glycemic control among youth with diabetes. *Pediatrics*. 2011;128(4):e839-e847. doi:10.1542/peds.2010-3636

9. Coddington DA, Hisnanick JJ. Clinical characteristics of non-insulin-dependent diabetes mellitus among southwestern American Indian youths. *J Health Popul Nutr*. 2001;19(1):12-17. https://www.ncbi.nlm.nih.gov/pubmed/11394178

10. Colvin CW, Ashraf AP, Griffin RL, et al. Diabetes Status and Race are Associated with Cardiovascular Risk Markers in Obese Adolescents. *Endocr Pract*. 2015;21(2):165-173. doi:10.4158/EP14087.OR

11. Craig ME, Femia G, Broyda V, Lloyd M, Howard NJ. Type 2 diabetes in Indigenous and non-Indigenous children and adolescents in New South Wales. *Med J Aust*. 2007;186(10):497-499. https://www.ncbi.nlm.nih.gov/pubmed/17516894

12. Dart AB, Sellers EA, Dean HJ. Kidney disease and youth onset type 2 diabetes: considerations for the general practitioner. *Int J Pediatr*. 2012;2012:237360. doi:10.1155/2012/237360

13. de Boer IH, Rue TC, Hall YN, Heagerty PJ, Weiss NS, Himmelfarb J. Temporal trends in the prevalence of diabetic kidney disease in the United States. *JAMA*. 2011;305(24):2532-2539. doi:10.1001/jama.2011.861

14. Dean HJ, Mundy RL, Moffatt M. Non-insulin-dependent diabetes mellitus in Indian children in Manitoba. *CMAJ*. 1992;147(1):52-57. https://www.ncbi.nlm.nih.gov/pubmed/1393888

15. Ehtisham S, Hattersley AT, Dunger DB, Barrett TG, British Society for Paediatric Endocrinology and Diabetes Clinical Trials Group. First UK survey of paediatric type 2 diabetes and MODY. *Arch Dis Child*. 2004;89(6):526-529. doi:10.1136/adc.2003.027821

16. Fourtner SH, Weinzimer SA, Levitt Katz LE. Hyperglycemic hyperosmolar non-ketotic syndrome in children with type 2 diabetes*. *Pediatr Diabetes*. 2005;6(3):129-135. doi:10.1111/j.1399-543X.2005.00113.x

17. Fu J-F, Liang L, Gong C-X, et al. Status and trends of diabetes in Chinese children: analysis of data from 14 medical centers. *World J Pediatr*. 2013;9(2):127-134. doi:10.1007/s12519-013-0414-4

18. Glaser NS. Non-insulin-dependent diabetes mellitus in childhood and adolescence. *Pediatr Clin North Am*. 1997;44(2):307-337. doi:10.1016/s0031-3955(05)70479-x

19. Glaser NS, Jones KL. Non-insulin dependent diabetes mellitus in Mexican-American children. *West J Med*. 1998;168(1):11-16. https://www.ncbi.nlm.nih.gov/pubmed/9448482

20. Grinstein G, Muzumdar R, Aponte L, Vuguin P, Saenger P, DiMartino-Nardi J. Presentation and 5-year follow-up of type 2 diabetes mellitus in African-American and Caribbean-Hispanic adolescents. *Horm Res*. 2003;60(3):121-126. doi:10.1159/000072523

21. Gungor N, Thompson T, Sutton-Tyrrell K, Janosky J, Arslanian S. Early signs of cardiovascular disease in youth with obesity and type 2 diabetes. *Diabetes Care*. 2005;28(5):1219-1221. doi:10.2337/diacare.28.5.1219

22. Hayes R, Shrewsbury V, Chan A, Cowell C, Garnett S. Type 2 diabetes in children and adolescence: A retrospective review. *Obes Res Clin Pract*. 2012;6(S1):73. doi:10.1016/j.orcp.2012.08.150

23. Kitagawa T, Owada M, Urakami T, Tajima N. Epidemiology of type 1 (insulin-dependent) and type 2 (non-insulin-dependent) diabetes mellitus in Japanese children. *Diabetes Res Clin Pract*. 1994;24 Suppl:S7-S13. doi:10.1016/0168-8227(94)90221-6

24. Kotb NA, Gaber R, Salama M, Nagy HM, Elhendy A. Clinical and biochemical predictors of increased carotid intima-media thickness in overweight and obese adolescents with type 2 diabetes. *Diab Vasc Dis Res*. 2012;9(1):35-41. doi:10.1177/1479164111421804

25. Larkin ME, Walders-Abramson N, Hirst K, et al. Effects of comorbid conditions on health-related quality of life in youth with Type 2 diabetes: the TODAY clinical trial. *Diabetes Manag* . 2015;5(6):431-439. doi:10.2217/dmt.15.35

26. Lim PK, Sreedharan AV, Hui YC, et al. Audit on annual nephropathy screening in children with diabetes. *Pediatr Diabetes*. 2017;18(S25):116. doi:10.1111/pedi.12589

27. Liu LL, Lawrence JM, Davis C, et al. Prevalence of overweight and obesity in youth with diabetes in USA: the SEARCH for Diabetes in Youth study. *Pediatr Diabetes*. 2010;11(1):4-11. doi:10.1111/j.1399-5448.2009.00519.x

28. Mayer-Davis EJ, Ma B, Lawson A, et al. Cardiovascular disease risk factors in youth with type 1 and type 2 diabetes: implications of a factor analysis of clustering. *Metab Syndr Relat Disord*. 2009;7(2):89-95. doi:10.1089/met.2008.0046

29. McMahon SK, Haynes A, Ratnam N, et al. Increase in type 2 diabetes in children and adolescents in Western Australia. *Med J Aust*. 2004;180(9):459-461. https://www.ncbi.nlm.nih.gov/pubmed/15115424

30. Nandkeoliar MK, Dharmalingam M, Marcus SR. Diabetes mellitus in Asian Indian children and adolescents. *J Pediatr Endocrinol Metab*. 2007;20(10):1109-1114. doi:10.1515/jpem.2007.20.10.1109

31. Prendergast C, Gidding SS. Cardiovascular risk in children and adolescents with type 2 diabetes mellitus. *Curr Diab Rep*. 2014;14(2):454. doi:10.1007/s11892-013-0454-0

32. Prestel C, Paulo R, Shatat I, Bowlby DA, Lewis K. Improved detection of elevated blood pressure in children with type 2 diabetes mellitus after implementation of electronic medical record. *Endocr Rev*. 2015;36(S2).

33. Ramachandran A, Snehalatha C, Satyavani K, Sivasankari S, Vijay V. Type 2 diabetes in Asian-Indian urban children. *Diabetes Care*. 2003;26(4):1022-1025. doi:10.2337/diacare.26.4.1022

34. Ramirez JP, Bernui I, Gonzalez JS, et al. Obesity, insulin resistance and type 2 diabetes mellitus in female adolescents, Lima - Peru. *Ann Nutr Metab*. 2017;71(S2):775-776. doi:10.1159/000480486

35. Reinehr T, Schober E, Roth CL, Wiegand S, Holl R, DPV-Wiss Study Group. Type 2 diabetes in children and adolescents in a 2-year follow-up: insufficient adherence to diabetes centers. *Horm Res*. 2008;69(2):107-113. doi:10.1159/000111814

36. Schober E, Waldhoer T, Rami B, Hofer S, Austrian Diabetes Incidence Study Group. Incidence and time trend of type 1 and type 2 diabetes in Austrian children 1999-2007. *J Pediatr*. 2009;155(2):190-193.e1. doi:10.1016/j.jpeds.2009.03.010

37. Shiga K, Kikuchi N. Children with type 2 diabetes mellitus are at greater risk of macrovascular complications. *Pediatr Int*. 2009;51(4):563-567. doi:10.1111/j.1442-200X.2009.02836.x

38. Shikha D, Singla M, Walia R, Potter N, Mercado A, Winer N. Vascular compliance in lean, obese, and diabetic children and adolescents: a cross-sectional study in a minority population. *Cardiorenal Med*. 2014;4(3-4):161-167. doi:10.1159/000365937

39. Shikha D, Singla M, Walia R, et al. Ambulatory Blood Pressure Monitoring in Lean, Obese and Diabetic Children and Adolescents. *Cardiorenal Med*. 2015;5(3):183-190. doi:10.1159/000381629

40. Sillars BA, Davis WA, Kamber N, Davis TME. The epidemiology and characteristics of type 2 diabetes in urban, community-based young people. *Intern Med J*. 2010;40(12):850-854. doi:10.1111/j.1445-5994.2010.02372.x

41. Sugihara S, Sasaki N, Kohno H, et al. Survey of current medical treatments for childhood-onset type 2 diabetes mellitus in Japan. *Clin Pediatr Endocrinol*. 2005;14(2):65-75. doi:10.1297/cpe.14.65

42. Tezier A, Houdon L, Burlot K, Lang M, Pigeon Kerchiche P, Bismuth E. High proportion of type 2 diabetes among newly diagnosed children and adolescents in La Reunion Island, a French oversea territory. *Pediatr Diabetes*. 2016;17(S24):80. doi:10.1111/pedi.12451

43. Urbina EM, Kimball TR, McCoy CE, Khoury PR, Daniels SR, Dolan LM. Youth with obesity and obesity-related type 2 diabetes mellitus demonstrate abnormalities in carotid structure and function. *Circulation*. 2009;119(22):2913-2919. doi:10.1161/CIRCULATIONAHA.108.830380

44. Virant FS. Prevalence of asthma and its association with glycemic control among youth with diabetes. *Pediatrics*. 2012;130(S1):S25-S26. doi:10.1542/peds.2012-2183NN

45. Warsy AS, el-Hazmi MA. Diabetes mellitus, hypertension and obesity--common multifactorial disorders in Saudis. *East Mediterr Health J*. 1999;5(6):1236-1242. https://www.ncbi.nlm.nih.gov/pubmed/11924118

© 2021 Cioana M et al. *JAMA Network Open.*

46. Whalley GA, Gusso S, Hofman P, et al. Structural and functional cardiac abnormalities in adolescent girls with poorly controlled type 2 diabetes. *Diabetes Care*. 2009;32(5):883-888. doi:10.2337/dc08-2005

47. Wittmeier KDM, Wicklow BA, MacIntosh AC, et al. Hepatic steatosis and low cardiorespiratory fitness in youth with type 2 diabetes. *Obesity* . 2012;20(5):1034-1040. doi:10.1038/oby.2011.379

48. Mottl AK, Divers J, Dabelea D, et al. The dose-response effect of insulin sensitivity on albuminuria in children according to diabetes type. *Pediatr Nephrol*. 2016;31(6):933-940. doi:10.1007/s00467-015-3276-2

49. Guven A, Demir EG. Cardiovascular Risk and Long Term Follow-up of Turkish Children with Type 2 Diabetes: Single Center Experience. ESPE Abstracts. Published 2016. Accessed February 11, 2021. https://abstracts.eurospe.org/hrp/0086/hrp0086p2-p304

50. Campbell-Stokes PL, Taylor BJ, New Zealand Children's Diabetes Working Group. Prospective incidence study of diabetes mellitus in New Zealand children aged 0 to 14 years. *Diabetologia*. 2005;48(4):643-648. doi:10.1007/s00125-005-1697-3

51. Shah AS, El Ghormli L, Gidding SS, et al. Prevalence of arterial stiffness in adolescents with type 2 diabetes in the TODAY cohort: Relationships to glycemic control and other risk factors. *J Diabetes Complications*. 2018;32(8):740-745. doi:10.1016/j.jdiacomp.2018.05.013

52. Likitmaskul S, Wacharasindhu S, Rawdaree P, et al. Thailand diabetes registry project: type of diabetes, glycemic control and prevalence of microvascular complications in children and adolescents with diabetes. *J Med Assoc Thai*. 2006;89 Suppl 1:S10-S16. https://www.ncbi.nlm.nih.gov/pubmed/17715829

53. Bjornstad P, Hughan K, Kelsey MM, et al. Effect of Surgical Versus Medical Therapy on Diabetic Kidney Disease Over 5 Years in Severely Obese Adolescents With Type 2 Diabetes. *Diabetes Care*. 2020;43(1):187-195. doi:10.2337/dc19-0708

54. Divers J, Mayer-Davis EJ, Lawrence JM, et al. Trends in Incidence of Type 1 and Type 2 Diabetes Among Youths - Selected Counties and Indian Reservations, United States, 2002-2015. *MMWR Morb Mortal Wkly Rep*. 2020;69(6):161-165. doi:10.15585/mmwr.mm6906a3

55. Draffin S, Nashatker K, Lewis K, Paulo R, Bowlby D. Obesity in early childhood diabetes. *J Investig Med*. 2013;61(2):397. doi:10.231/JIM.0b013e3182820c55

56. Fortmeier-Saucier L, Savrin C, Heinzer M, Hudak C. BMI and lipid levels in Mexican American children diagnosed with type 2 diabetes. *Worldviews Evid Based Nurs*. 2008;5(3):142-147. doi:10.1111/j.1741-6787.2008.00122.x

57. Kershnar AK, Daniels SR, Imperatore G, et al. Lipid abnormalities are prevalent in youth with type 1 and type 2 diabetes: the SEARCH for Diabetes in Youth Study. *J Pediatr*. 2006;149(3):314-319. doi:10.1016/j.jpeds.2006.04.065

58. Lawrence JM, Liese AD, Liu L, et al. Weight-loss practices and weight-related issues among youth with type 1 or type 2 diabetes. *Diabetes Care*. 2008;31(12):2251-2257. doi:10.2337/dc08-0719

59. Likitmaskul S, Kiattisathavee P, Chaichanwatanakul K, Punnakanta L, Angsusingha K, Tuchinda C. Increasing prevalence of type 2 diabetes mellitus in Thai children and adolescents associated with increasing prevalence of obesity. *J Pediatr Endocrinol Metab*. 2003;16(1):71-77. doi:10.1515/jpem.2003.16.1.71

Exclusion Reason: Repeated Data (n = 21)

1. Amutha A, Anjana RM, Venkatesan U, et al. Incidence of complications in young-onset diabetes: Comparing type 2 with type 1 (the young diab study). *Diabetes Res Clin Pract*. 2017;123:1-8. doi:10.1016/j.diabres.2016.11.006

© 2021 Cioana M et al. *JAMA Network Open.*

2. Awa WL, Boehm BO, Rosinger S, et al. HLA-typing, clinical, and immunological characterization of youth with type 2 diabetes mellitus phenotype from the German/Austrian DPV database. *Pediatr Diabetes*. 2013;14(8):562-574. doi:10.1111/pedi.12043

3. Candler TP, Mahmoud O, Lynn RM, Majbar AA, Barrett TG, Shield JPH. Continuing rise of Type 2 diabetes incidence in children and young people in the UK. *Diabet Med*. 2018;35(6):737-744. doi:10.1111/dme.13609

4. Colvin CW, Harmon CM, Griffin RL, et al. Diabetes status and ethnicity affect cardiovascular risk factors in obese children. *Endocr Rev*. 2013;34(3S1).

5. TODAY Study Group. Rapid rise in hypertension and nephropathy in youth with type 2 diabetes: the TODAY clinical trial. *Diabetes Care*. 2013;36(6):1735-1741. doi:10.2337/dc12-2420

6. Haynes A, Smith GJ, Jones TW, Davis EA. Distinct clinical characteristics of pediatric patients diagnosed with type 1 and type 2 diabetes in a contemporary population-based cohort in Western Australia (1999-2015). *Pediatr Diabetes*. 2016;17(S24):94. doi:10.1111/pedi.12451

7. Ievers-Landis CE, Walders-Abramson N, Amodei N, et al. Longitudinal Correlates of Health Risk Behaviors in Children and Adolescents with Type 2 Diabetes. *J Pediatr*. 2015;166(5):1258-1264.e3. doi:10.1016/j.jpeds.2015.01.019

8. Ievers-Landis CE, Yasuda P, Walders-Abramson N, et al. Longitudinal correlates of youth health risk behaviors among children and adolescents with type 2 diabetes in the TODAY study. *Diabetes*. 2013;62(S1):A344. doi:10.2337/db13-859-1394

9. Kim G, Divers J, Fitzgerald N, et al. Prevalence of CVD risk factors over time among youth with type 1 and type 2 diabetes. *Diabetes*. 2014;63(S1):A160. doi:10.2337/db14-389-664

10. Klingensmith GJ, Lanzinger S, Tamborlane WV, et al. Characteristics and treatment of adolescents with type 2 diabetes (T2D): Comparison of U.S. Pediatric diabetes consortium (PDC) and Germany/Austria/Luxemburg pediatric diabetes prospective follow-up (DPV) cohorts. *Diabetes*. 2017;66(S1):A368.

11. Li H-Y, Wei J-N, Sung F-C, Chuang L-M. Higher rate of obesity and hypertension in adolescents with type 2 diabetes than in those with type 1 diabetes. *Diabetes Care*. 2006;29(10):2326. doi:10.2337/dc06-1265

12. Rodriguez BL, Fujimoto WY, Mayer-Davis EJ, et al. Prevalence of cardiovascular disease risk factors in U.S. children and adolescents with diabetes: the SEARCH for diabetes in youth study. *Diabetes Care*. 2006;29(8):1891-1896. doi:10.2337/dc06-0310

13. Schober E, Rami B, Grabert M, et al. Phenotypical aspects of maturity-onset diabetes of the young (MODY diabetes) in comparison with Type 2 diabetes mellitus (T2DM) in children and adolescents: experience from a large multicentre database. *Diabet Med*. 2009;26(5):466-473. doi:10.1111/j.1464-5491.2009.02720.x

14. Tryggestad JB, Willi SM. Complications and comorbidities of T2DM in adolescents: findings from the TODAY clinical trial. *J Diabetes Complications*. 2015;29(2):307-312. doi:10.1016/j.jdiacomp.2014.10.009

15. West NA, Hamman RF, Mayer-Davis EJ, et al. Cardiovascular risk factors among youth with and without type 2 diabetes: differences and possible mechanisms. *Diabetes Care*. 2009;32(1):175-180. doi:10.2337/dc08-1442

16. White NH, Pyle L, Tamborlane WV, Geffner ME, Guandalini C. Clinical characteristics and co-morbidities in a large cohort of youth with type 2 diabetes mellitus (T2DM) screened for the treatment options for type 2 diabetes in adolescents and youth (TODAY) study. *Diabetes*. 2009;58(S1A).

17. Klingensmith GJ, Lanzinger S, Tamborlane WV, et al. Adolescent type 2 diabetes: Comparing the Pediatric Diabetes Consortium and Germany/Austria/Luxemburg Pediatric Diabetes Prospective registries. *Pediatr Diabetes*. 2018;19(7):1156-1163. doi:10.1111/pedi.12712

© 2021 Cioana M et al. *JAMA Network Open.*

18. Van Name M, Tamborlane WV, Cheng P, et al. Therapeutic inertia: Underdiagnosed and untreated hypertension and dyslipidemia in the pediatric diabetes consortium (PDC) type 2 diabetes (T2D) registry. *Diabetes*. 2016;65(S1):A334. doi:10.2337/db16-861-1374

19. Kim G, Divers J, Fino NF, et al. Trends in prevalence of cardiovascular risk factors from 2002 to 2012 among youth early in the course of type 1 and type 2 diabetes. The SEARCH for Diabetes in Youth Study. *Pediatr Diabetes*. 2019;20(6):693-701. doi:10.1111/pedi.12846

20. Bjornstad P, Laffel L, Lynch J, et al. Elevated Serum Uric Acid Is Associated With Greater Risk for Hypertension and Diabetic Kidney Diseases in Obese Adolescents With Type 2 Diabetes: An Observational Analysis From the Treatment Options for Type 2 Diabetes in Adolescents and Youth (TODAY) Study. *Diabetes Care*. 2019;42(6):1120-1128. doi:10.2337/dc18-2147

21. Awa WL, Fach E, Krakow D, et al. Type 2 diabetes from pediatric to geriatric age: analysis of gender and obesity among 120,183 patients from the German/Austrian DPV database. *Eur J Endocrinol*. 2012;167(2):245-254. doi:10.1530/EJE-12-0143

Exclusion Reason: Irrelevant (n = 10)

1. Anderson B, Levitt Katz L, McKay S, et al. Study medication adherence and outcomes in the TODAY cohort of youth with type 2 diabetes (T2D). *Pediatr Diabetes*. 2014;15(S19):22. doi:10.1111/pedi.12194_1

2. Bjornstad P, Maahs DM, Cherney DZ, et al. Insulin sensitivity is an important determinant of renal health in adolescents with type 2 diabetes. *Diabetes Care*. 2014;37(11):3033-3039. doi:10.2337/dc14-1331

3. Clark PA. Type 2 diabetes in youth. *J S C Med Assoc*. 2009;105(2):51-54. https://www.ncbi.nlm.nih.gov/pubmed/19480126

4. Gidding SS, Lima J, Pyle L, et al. Relationship of LV Mass, LA Diameter, and LV Geometry to Cardiovascular Risk Factors in the Today Cohort of Adolescents with Type 2 Diabetes (T2D): Abstract 13132. *Circulation*. 2012;126(21S1).

5. Günther ALB, Liese AD, Bell RA, et al. Association between the dietary approaches to hypertension diet and hypertension in youth with diabetes mellitus. *Hypertension*. 2009;53(1):6-12. doi:10.1161/HYPERTENSIONAHA.108.116665

6. Katz SL, MacLean JE, Hoey L, et al. Insulin Resistance and Hypertension in Obese Youth With Sleep-Disordered Breathing Treated With Positive Airway Pressure: A Prospective Multicenter Study. *J Clin Sleep Med*. 2017;13(9):1039-1047. doi:10.5664/jcsm.6718

7. Kehler DS, Stammers AN, Susser SE, et al. Cardiovascular complications of type 2 diabetes in youth. *Biochem Cell Biol*. 2015;93(5):496-510. doi:10.1139/bcb-2014-0118

8. Lawrence JM. Updates from SEARCH for diabetes in youth study: Quality of life in youth with type 1 diabetes and type 2 diabetes. *Pediatr Diabetes*. 2011;12(S15):7. doi:10.1111/j.1399-5448.2011.01816.x

9. Spurr S, Bally J, Bullin C, Allan D, McNair E. The prevalence of undiagnosed Prediabetes/type 2 diabetes, prehypertension/hypertension and obesity among ethnic groups of adolescents in Western Canada. *BMC Pediatr*. 2020;20(1):31. doi:10.1186/s12887-020-1924-6

10. Alaqeel AA. Pediatric diabetes in Saudi Arabia: Challenges and potential solutions. A review article. *Int J Pediatr Adolesc Med*. 2019;6(4):125-130. doi:10.1016/j.ijpam.2019.05.008

Exclusion Reason: Review (n = 7)

1. Bogdanović R. Diabetic nephropathy in children and adolescents. *Pediatr Nephrol*. 2008;23(4):507-525. doi:10.1007/s00467-007-0583-2

2. Fagot-Campagna A, Pettitt DJ, Engelgau MM, et al. Type 2 diabetes among North American children and adolescents: an epidemiologic review and a public health perspective. *J Pediatr*. 2000;136(5):664-672. doi:10.1067/mpd.2000.105141

3. Pinhas-Hamiel O, Zeitler P. Acute and chronic complications of type 2 diabetes mellitus in children and adolescents. *Lancet*. 2007;369(9575):1823-1831. doi:10.1016/S0140-6736(07)60821-6

4. Zeitler PS. Type 2 diabetes in children: recognition and complications. *Ped Health*. 2010;4(2):123-127. doi:10.2217/phe.10.10

5. Titmuss A, Davis EA, Brown A, Maple-Brown LJ. Emerging diabetes and metabolic conditions among Aboriginal and Torres Strait Islander young people. *Med J Aust*. 2019;210(3):111-113.e1. doi:10.5694/mja2.13002

6. Graves LE, Donaghue KC. Management of diabetes complications in youth. *Ther Adv Endocrinol Metab*. 2019;10:2042018819863226. doi:10.1177/2042018819863226

7. Clifton P. What have nutritional trials taught us in diabetes in children and adolescents? *Pediatr Diabetes*. 2015;16(S21):18. doi:10.1111/pedi.12307

Exclusion Reason: Risk of Diabetes (n = 4)

1. Bhatia V, IAP National Task Force for Childhood Prevention of Adult Diseases. IAP National Task Force for Childhood Prevention of Adult Diseases: insulin resistance and Type 2 diabetes mellitus in childhood. *Indian Pediatr*. 2004;41(5):443-457. https://www.ncbi.nlm.nih.gov/pubmed/15181295

2. Twig G, Reichman B, Afek A, et al. Severe obesity and cardio-metabolic comorbidities: a nationwide study of 2.8 million adolescents. *Int J Obes* . 2019;43(7):1391-1399. doi:10.1038/s41366-018-0213-z

3. Young TK, Dean HJ, Flett B, Wood-Steiman P. Childhood obesity in a population at high risk for type 2 diabetes. *J Pediatr*. 2000;136(3):365-369. doi:10.1067/mpd.2000.103504

4. Fukui M, Tanaka M, Toda H, et al. Risk factors for development of diabetes mellitus, hypertension and dyslipidemia. *Diabetes Res Clin Pract*. 2011;94(1):e15-e18. doi:10.1016/j.diabres.2011.07.006

Exclusion Reason: Not T2DM (n = 4)

1. Diabetes Control and Complications Trial Research Group. Effect of intensive diabetes treatment on the development and progression of long-term complications in adolescents with insulin-dependent diabetes mellitus: Diabetes Control and Complications Trial. *J Pediatr*. 1994;125(2):177-188. doi:10.1016/s0022-3476(94)70190-3

2. Holl RW, Lang G, Grabert M, Teller W, Heinze E. LATE COMPLICATIONS OF DIABETES-MELLITUS - DOES PREVENTION START IN CHILDHOOD. *Monatsschr Kinderheilkd*. 1995;143(3S1):S12-S25.

3. Krishna Prasad H, White NH. Microalbuminuria (MA) in youth with childhood-onset diabetes at a large university-based clinic. *Pediatr Diabetes*. 2013;14(S18):62.

© 2021 Cioana M et al. *JAMA Network Open.*

4. Hamilton L, Wilson DP, O'Reilly CM, Gonzalez J, de la Torre A. The Prevalence of Dyslipidemia and Other Cardiovascular Risk Factors in Youth with Diabetes. *Curr Pediatr Rev*. 2017;13(4):277-280. doi:10.2174/1573396314666180221163924

© 2021 Cioana M et al. *JAMA Network Open.*

eReferences.

1. Stroup DF, Berlin JA, Morton SC, et al. Meta-analysis of Observational Studies in Epidemiology: A Proposal for Reporting. *JAMA*. 2000;283(15):2008-2012. doi:10.1001/jama.283.15.2008

2. Drutel R, Paulo R. Prevalence of Hypertension Among Children with Diabetes Mellitus. *Hypertension*. 2014;64(Suppl 1):A660. doi:10.1161/hyp.64.suppl_1.660

3. Haynes A, Kalic R, Curran J, et al. Type 2 diabetes and associated complications in Western Australian children: a population-based study (1990-2012). *Diabetologia*. 2014;57(Suppl 1):S510-S510.

4. Yafi M. The prevalence of microalbuminuria in children with type 2 diabetes mellitus (T2DM). *Arch Dis Child*. 2019;104(Suppl 3):A250.

5. Osman HAM, Elsadek N, Abdullah MA. Type 2 diabetes in Sudanese children and adolescents. *Sudan J Paediatr*. 2013;13(2):17-23.

6. Balasanthiran A, O'Shea T, Moodambail A, et al. Type 2 diabetes in children and young adults in East London: an alarmingly high prevalence. *Pract Diabetes*. 2012;29(5):193-198a. doi:10.1002/pdi.1689

7. Pérez-Perdomo R, Pérez-Cardona CM, Allende-Vigo M, Rivera-Rodríguez MI, Rodríguez-Lugo LA. Type 2 diabetes mellitus among youth in Puerto Rico, 2003. *P R Health Sci J*. 2005;24(2):111-117.

8. Ettinger LM, Freeman K, DiMartino-Nardi JR, Flynn JT. Microalbuminuria and Abnormal Ambulatory Blood Pressure in Adolescents With Type 2 Diabetes Mellitus. *J Pediatr*. 2005;147(1):67-73. doi:10.1016/j.jpeds.2005.02.003

9. Scott CR, Smith JM, Cradock MM, Pihoker C. Characteristics of Youth-onset Noninsulin-dependent Diabetes Mellitus and Insulin-dependent Diabetes Mellitus at Diagnosis. *Pediatrics*. 1997;100(1):84-91. doi:10.1542/peds.100.1.84

10. Zdravkovic V, Daneman D, Hamilton J. Presentation and course of Type 2 diabetes in youth in a large multi-ethnic city. *Diabet Med*. 2004;21(10):1144-1148. doi:10.1111/j.1464-5491.2004.01297.x

11. Reinehr T, Andler W, Kapellen T, et al. Clinical Characteristics of Type 2 Diabetes Mellitus in Overweight European Caucasian Adolescents. *Exp Clin Endocrinol Diabetes*. 2005;113(03):167-170. doi:10.1055/s-2005-837522

12. Scott A, Whitcombe S, Bouchier D, Dunn P. Diabetes in children and young adults in Waikato Province, New Zealand: outcomes of care. *NZMJ*. 2004;117(1207).

13. Unnikrishnan A. G., Bhatia Eesh, Bhatia Vijayalakshmi, et al. Type 1 Diabetes versus Type 2 Diabetes with Onset in Persons Younger than 20 Years of Age. *Ann N Y Acad Sci*. 2008;1150(1):239-244. doi:10.1196/annals.1447.056

14. Aulich J, Cho YH, Januszewski AS, et al. Associations between circulating inflammatory markers, diabetes type and complications in youth. *Pediatr Diabetes*. 2019;20(8):1118-1127. doi:10.1111/pedi.12913

15. Yeow TP, Aun ES-Y, Hor CP, Lim SL, Khaw CH, Aziz NA. Challenges in the classification and management of Asian youth-onset diabetes mellitus- lessons learned from a single centre study. *PLoS ONE*. 2019;14(1). doi:10.1371/journal.pone.0211210

16. Curran JA, Haynes A, Davis EA. Clinical characteristics of Western Australian children diagnosed with type 2 diabetes before 10 years of age. *Med J Aust*. 2020;212(2):95-95.e1. doi:10.5694/mja2.50451

© 2021 Cioana M et al. *JAMA Network Open.*

17. Khalil SA, Megallaa MH, Rohoma KH, et al. Prevalence of Chronic Diabetic Complications in Newly Diagnosed versus Known Type 2 Diabetic Subjects in a Sample of Alexandria Population, Egypt. *Curr Diabetes Rev*. 2019;15(1):74-83. doi:10.2174/1573399814666180125100917

18. Amutha A, Datta M, Unnikrishnan R, Anjana RM, Mohan V. Clinical profile and complications of childhood- and adolescent-onset type 2 diabetes seen at a diabetes center in south India. *Diabetes Technol Ther*. 2012;14(6):497-504. doi:10.1089/dia.2011.0283

19. Jefferies C, Carter P, Reed PW, et al. The incidence, clinical features, and treatment of type 2 diabetes in children <15 yr in a population-based cohort from Auckland, New Zealand, 1995–2007. *Pediatr Diabetes*. 2012;13(4):294-300. doi:10.1111/j.1399-5448.2012.00851.x

20. Scott A, Toomath R, Bouchier D, et al. First national audit of the outcomes of care in young people with diabetes in New Zealand: high prevalence of nephropathy in Maori and Pacific Islanders. *N Z Med J*. 2006;119(1235):U2015.

21. Urakami T, Suzuki J, Yoshida A, et al. Prevalence of components of the metabolic syndrome in schoolchildren with newly diagnosed type 2 diabetes mellitus. *Pediatr Diabetes*. 2009;10(8):508-512. doi:10.1111/j.1399-5448.2009.00533.x

22. Dart AB, Martens PJ, Rigatto C, Brownell MD, Dean HJ, Sellers EA. Earlier Onset of Complications in Youth With Type 2 Diabetes. *Diabetes Care*. 2014;37(2):436-443. doi:10.2337/dc13-0954

23. Amed S, Hamilton JK, Sellers EAC, et al. Differing clinical features in Aboriginal vs. non-Aboriginal children presenting with type 2 diabetes. *Pediatr Diabetes*. 2012;13(6):470-475. doi:10.1111/j.1399-5448.2012.00859.x

24. Pinhas-Hamiel O, Dolan LM, Daniels SR, Standiford D, Khoury PR, Zeitler P. Increased incidence of non-insulin-dependent diabetes mellitus among adolescents. *J Pediatr*. 1996;128(5):608-615. doi:10.1016/S0022-3476(96)80124-7

25. Hotu S, Carter B, Watson PD, Cutfield WS, Cundy T. Increasing prevalence of type 2 diabetes in adolescents. *J Paediatr Child Health*. 2004;40(4):201-204. doi:10.1111/j.1440-1754.2004.00337.x

26. Ruhayel SD, James RA, Ehtisham S, Cameron FJ, Werther GA, Sabin MA. An observational study of type 2 diabetes within a large Australian tertiary hospital pediatric diabetes service. *Pediatr Diabetes*. 2010;11(8):544-551. doi:10.1111/j.1399-5448.2010.00647.x

27. Schmidt F, Kapellen TM, Wiegand S, et al. Diabetes Mellitus in Children and Adolescents with Genetic Syndromes. *Exp Clin Endocrinol Diabetes*. 2012;120(10):579-585. doi:10.1055/s-0032-1306330

28. Maahs DM, Snively BM, Bell RA, et al. Higher Prevalence of Elevated Albumin Excretion in Youth With Type 2 Than Type 1 Diabetes: The SEARCH for Diabetes in Youth Study. *Diabetes Care*. 2007;30(10):2593-2598. doi:10.2337/dc07-0450

29. Sellers EAC, Blydt-Hansen TD, Dean HJ, Gibson IW, Birk PE, Ogborn M. Macroalbuminuria and Renal Pathology in First Nation Youth With Type 2 Diabetes. *Diabetes Care*. 2009;32(5):786-790. doi:10.2337/dc08-1828

30. Candler TP, Mahmoud O, Lynn RM, Majbar AA, Barrett TG, Shield JPH. Continuing rise of Type 2 diabetes incidence in children and young people in the UK. *Diabet Med*. 2018;35(6):737-744. doi:10.1111/dme.13609

31. Dart AB, Wicklow B, Blydt-Hansen TD, et al. A Holistic Approach to Risk for Early Kidney Injury in Indigenous Youth With Type 2 Diabetes: A Proof of Concept Paper From the iCARE Cohort. *Can J Kidney Health Dis*. 2019;6. doi:10.1177/2054358119838836

© 2021 Cioana M et al. *JAMA Network Open.*

32. Eppens MC, Craig ME, Cusumano J, et al. Prevalence of diabetes complications in adolescents with type 2 compared with type 1 diabetes. *Diabetes Care*. 2006;29(6):1300-1306. doi:10.2337/dc05-2470

33. Farah SE, Wals KT, Friedman IB, Pisacano MA, DiMartino-Nardi J. Prevalence of Retinopathy and Microalbuminuria in Pediatric Type 2 Diabetes Mellitus. *J Pediatr Endocrinol Metab*. 2006;19(7). doi:10.1515/JPEM.2006.19.7.937

34. Yoo E-G, Choi IK, Kim DH. Prevalence of microalbuminuria in young patients with type 1 and type 2 diabetes mellitus. *J Pediatr Endocrinol Metab JPEM*. 2004;17(10):1423-1427.

35. Sellers EAC, Hadjiyannakis S, Amed S, et al. Persistent Albuminuria in Children with Type 2 Diabetes: A Canadian Paediatric Surveillance Program Study. *J Pediatr*. 2016;168:112-117. doi:10.1016/j.jpeds.2015.09.042

36. Calagua Quispe M, Del Aguila Villar C, Nuñez Almache O, et al. Clinical Features and Course of Pediatric Patients with Type 1 and Type 2 Diabetes Mellitus. *Horm Res Paediatr*. 2015;84(Suppl 2):39.

37. Zabeen B, Nahar J, Tayyeb S, Nhar N, Azad K. Type 2 diabetes in Bangladeshi children and adolescents - an emerging problem. *Pediatr Diabetes*. 2016;17(Suppl 24):80-S81.

38. Newton K, Stanley J, Wiltshire E. Audit of type 2 diabetes in youth in Wellington, New Zealand 2001–2013. *Pediatr Diabetes*. 2015;16(Suppl 21):50-150. doi:10.1111/pedi.12309

39. Son MK, Yoo HY, Kwak BO, et al. Regression and progression of microalbuminuria in adolescents with childhood onset diabetes mellitus. *Ann Pediatr Endocrinol Metab*. 2015;20(1):13-20. doi:10.6065/apem.2015.20.1.13

40. Nambam B, Silverstein J, Cheng P, et al. A cross-sectional view of the current state of treatment of youth with type 2 diabetes in the USA: enrollment data from the Pediatric Diabetes Consortium Type 2 Diabetes Registry. *Pediatr Diabetes*. 2017;18(3):222-229. doi:10.1111/pedi.12377

41. Dart AB, Sellers EA, Martens PJ, Rigatto C, Brownell MD, Dean HJ. High Burden of Kidney Disease in Youth-Onset Type 2 Diabetes. *Diabetes Care*. 2012;35(6):1265-1271. doi:10.2337/dc11-2312

42. Bell RA, Mayer-Davis EJ, Beyer JW, et al. Diabetes in Non-Hispanic White Youth. *Diabetes Care*. 2009;32(Suppl 2):S102-S111. doi:10.2337/dc09-S202

43. Copeland KC, Zeitler P, Geffner M, et al. Characteristics of adolescents and youth with recent-onset type 2 diabetes: the TODAY cohort at baseline. *J Clin Endocrinol Metab*. 2011;96(1):159-167. doi:10.1210/jc.2010-1642

44. Cruz M, Torres M, Aguilar-Herrera B, et al. Type 2 Diabetes Mellitus in Children - An Increasing Health Problem in Mexico. *J Pediatr Endocrinol Metab*. 2004;17(2). doi:10.1515/JPEM.2004.17.2.183

45. Dabelea D, DeGroat J, Sorrelman C, et al. Diabetes in Navajo Youth. *Diabetes Care*. 2009;32(Suppl 2):S141-S147. doi:10.2337/dc09-S206

46. Eppens MC, Craig ME, Jones TW, et al. Type 2 diabetes in youth from the Western Pacific region: glycaemic control, diabetes care and complications. *Curr Med Res Opin*. 2006;22(5):1013-1020. doi:10.1185/030079906X104795

47. Holman N, Campbell F, Sattar N, Warner J. Microvascular disease among young people with diabetes. *Diabet Med*. 2015;32(Supplement 1):16. doi:10.1111/dme.12665_8

© 2021 Cioana M et al. *JAMA Network Open.*

48. Kim NH, Pavkov ME, Knowler WC, et al. Predictive value of albuminuria in American Indian youth with or without type 2 diabetes. *Pediatrics*. 2010;125(4):e844-e851. doi:10.1542/peds.2009-1230

49. Klingensmith GJ, Connor CG, Ruedy KJ, et al. Presentation of youth with type 2 diabetes in the Pediatric Diabetes Consortium. *Pediatr Diabetes*. 2016;17(4):266-273. doi:10.1111/pedi.12281

50. Lawrence JM, Mayer-Davis EJ, Reynolds K, et al. Diabetes in Hispanic American Youth. *Diabetes Care*. 2009;32(Suppl 2):S123-S132. doi:10.2337/dc09-S204

51. Le PT, Huisingh CE, Ashraf AP. Glycemic control and diabetic dyslipidemia in adolescents with type 2 diabetes. *Endocr Pract Off J Am Coll Endocrinol Am Assoc Clin Endocrinol*. 2013;19(6):972-979. doi:10.4158/EP13016.OR

52. Liu LL, Yi JP, Beyer J, et al. Type 1 and Type 2 Diabetes in Asian and Pacific Islander U.S. Youth. *Diabetes Care*. 2009;32(Suppl 2):S133-S140. doi:10.2337/dc09-S205

53. Mayer-Davis EJ, Beyer J, Bell RA, et al. Diabetes in African American youth: prevalence, incidence, and clinical characteristics: the SEARCH for Diabetes in Youth Study. *Diabetes Care*. 2009;32 Suppl 2:S112-122. doi:10.2337/dc09-S203

54. Pelham JH, Hanks L, Aslibekyan S, Dowla S, Ashraf AP. Higher hemoglobin A1C and atherogenic lipoprotein profiles in children and adolescents with type 2 diabetes mellitus. *J Clin Transl Endocrinol*. 2018;15:30-34. doi:10.1016/j.jcte.2018.11.006

55. Rodriguez BL, Dabelea D, Liese AD, et al. Prevalence and Correlates of Elevated Blood Pressure in Youth with Diabetes Mellitus: The Search for Diabetes in Youth Study. *J Pediatr*. 2010;157(2):245-251.e1. doi:10.1016/j.jpeds.2010.02.021

56. Sellers EA, Yung G, Dean HJ. Dyslipidemia and other cardiovascular risk factors in a Canadian First Nation pediatric population with type 2 diabetes mellitus. *Pediatr Diabetes*. 2007;8(6):384-390. doi:10.1111/j.1399-5448.2007.00284.x

57. Shalitin S, Tauman R, Meyerovitch J, Sivan Y. Are frequency and severity of sleep-disordered breathing in obese children and youth with and without type 2 diabetes mellitus different? *Acta Diabetol*. 2014;51(5):757-764. doi:10.1007/s00592-014-0583-1

58. Shield JPH, Lynn R, Wan KC, Haines L, Barrett TG. Management and 1 year outcome for UK children with type 2 diabetes. *Arch Dis Child*. 2009;94(3):206-209. doi:10.1136/adc.2008.143313

59. Upchurch SL, Brosnan CA, Meininger JC, et al. Characteristics of 98 Children and Adolescents Diagnosed With Type 2 Diabetes by Their Health Care Provider at Initial Presentation. *Diabetes Care*. 2003;26(7):2209-2209. doi:10.2337/diacare.26.7.2209

60. Wei J-N, Sung F-C, Lin C-C, Lin R-S, Chiang C-C, Chuang L-M. National Surveillance for Type 2 Diabetes Mellitus in Taiwanese Children. *JAMA*. 2003;290(10):1345-1350. doi:10.1001/jama.290.10.1345

61. Zabeen B, Nahar J, Tayyeb S, Mohsin F, Nahar N, Azad K. Characteristics of children and adolescents at onset of type 2 diabetes in a Tertiary Hospital in Bangladesh. *Indian J Endocrinol Metab*. 2016;20(5):638-642. doi:10.4103/2230-8210.190544

© 2021 Cioana M et al. *JAMA Network Open.*
